# Microglia regulate nucleus accumbens synaptic development and circuit function underlying threat avoidance behaviors in mice

**DOI:** 10.1038/s41467-026-77487-z

**Published:** 2026-09-26

**Authors:** Michael W. Gongwer, Fanny Etienne, Eric N. Moca, Sara V. Blagburn-Blanco, Megan S. Chappell, Keionna A. Newton, Daniel T. Gray, Jack P. Riley, Chloe R. Christensen, Russell N. Ahmed, Alexander S. Enos, Melody Haratian, Alex Qi, Rocio Rojo, Scott A. Wilke, Clare Pridans, Laura A. DeNardo, Lindsay M. De Biase

**Affiliations:** 1 Department of Physiology, University of California, Los Angeles, CA USA; 2 Neuroscience Interdepartmental Program, University of California, Los Angeles, CA USA; 3 UCLA Medical Scientist Training Program, University of California, Los Angeles, CA USA; 4 Department of Psychiatry, University of California, Los Angeles, CA USA; 5 Institution for Regeneration and Repair, University of Edinburgh, Edinburgh, UK; 6 Tecnologico de Monterrey, Escuela de Medicina y Ciencias de la Salud, Monterrey, Mexico

**Keywords:** Microglia, Synaptic development

## Abstract

While microglia are well known for pruning synapses during neurodevelopment, their role in synapse formation remains understudied. Here we investigate how microglial absence impacts synaptic development in the nucleus accumbens (NAc), a region critical for emotional regulation and motivated behaviors. Using genetically modified mice lacking microglia (*Csf1r*^ΔFIRE/ΔFIRE^), we find blunted excitatory synapse formation in the NAc, particularly during the second and third postnatal weeks. Tissue-level NAc proteomics in the third postnatal week reveals changes in proteins involved in synapse structure, trans-synaptic signaling, and pre-synaptic function. Although electrophysiological changes largely resolve by adulthood, microglia-lacking mice exhibit persistent changes in threat avoidance behavior and underlying NAc neuronal activity. Transient depletion of NAc microglia during the second postnatal week similarly disrupts synapse formation and adult threat avoidance. Together, these findings identify a role for microglia in shaping NAc synaptic development and in establishing functional circuits that support adult behavioral function.

## Abstract (web summary)

Microglial synapse removal has been heavily studied. Here, the authors reveal roles for microglia in synapse formation by showing that absence of nucleus accumbens microglia during development reduces synapse number and alters avoidance behaviors.

## Introduction

The development of neural circuits relies on coordinated molecular and cellular interactions between neurons and glial cells. Key examples of these interactions include microglial regulation of axon guidance, regulation of programmed cell death, and regulation of synapse number via synaptic pruning^1–6^. A few studies have shown that microglia can also promote synapse formation and synapse maturation^7–9^, but these microglial contributions to synaptic development are much less studied. In addition, much of what we know about microglia in the developing brain comes from studies of the cortex and primary sensory circuits during restricted developmental time periods. This leaves substantial gaps in our understanding of microglial contributions to neural development.

The limbic system, including the nucleus accumbens (NAc), is important for emotional regulation and motivated behaviors^10^. The NAc mediates both reward approach and threat avoidance behaviors, and NAc dysfunction is implicated in a variety of psychiatric disorders that arise early in life^11,12^. However, our understanding of developmental trajectories of the NAc, and how they relate to subsequent behaviors, is lacking. Microglia have emerged as key regulators of synaptic development and are highly sensitive to environmental challenges that increase risk for psychiatric disorders. Given this, it is critical to understand how microglia shape synaptic development within limbic centers.

Maturation of neuronal populations and synaptic connections throughout the brain occurs in a highly coordinated, *region-specific* fashion^13,14^. We recently showed that microglial development is region-specific, with microglial density and morphology following distinct developmental trajectories in different regions of mesolimbic dopamine circuitry^15^. In the NAc, microglia displayed a robust overproduction during the second and third postnatal weeks, peaking at nearly double the density observed in the adult brain. In contrast, the ventral tegmental area (VTA) only exhibited a mild increase in microglial density during this period, suggesting a unique role for microglia in the developing NAc. The time course of NAc microglial overproduction did not align with programmed cell death or established periods of synaptic pruning, which occur earlier or later in development, respectively^15,16^. Studies in other striatal subregions indicate that the second and third postnatal weeks represent a period of pronounced synaptogenesis and refinement of synaptic kinetics^17,18^, suggesting that microglial overproduction during this time window may be related to their support of these processes within the NAc.

In this study, we used patch clamp electrophysiology, confocal microscopy, proteomics, and fiber photometry during behavioral assays in mice that possess or lack microglia to define potential roles for microglia in NAc synaptic development. We mapped the time course of synapse formation within the NAc and showed that the lack of microglia perturbs synapse formation during key developmental windows. We leveraged tissue proteomics to reveal how absence of microglia changes the landscape of synaptic proteins in the developing NAc. Although synaptic changes were less pronounced in adults, mice lacking microglia had altered activity in the NAc during a threat avoidance behavioral assay. Transient depletion of NAc microglia reproduced synaptic deficits as well as some behavioral effects of lifelong microglial absence. Together these studies reveal how microglia regulate NAc developmental trajectories and adult behavioral repertoires and add to our understanding of region-specific roles for microglia during brain development.

## Results

### Absence of microglia blunts NAc excitatory synaptogenesis

**Fig. 1:**
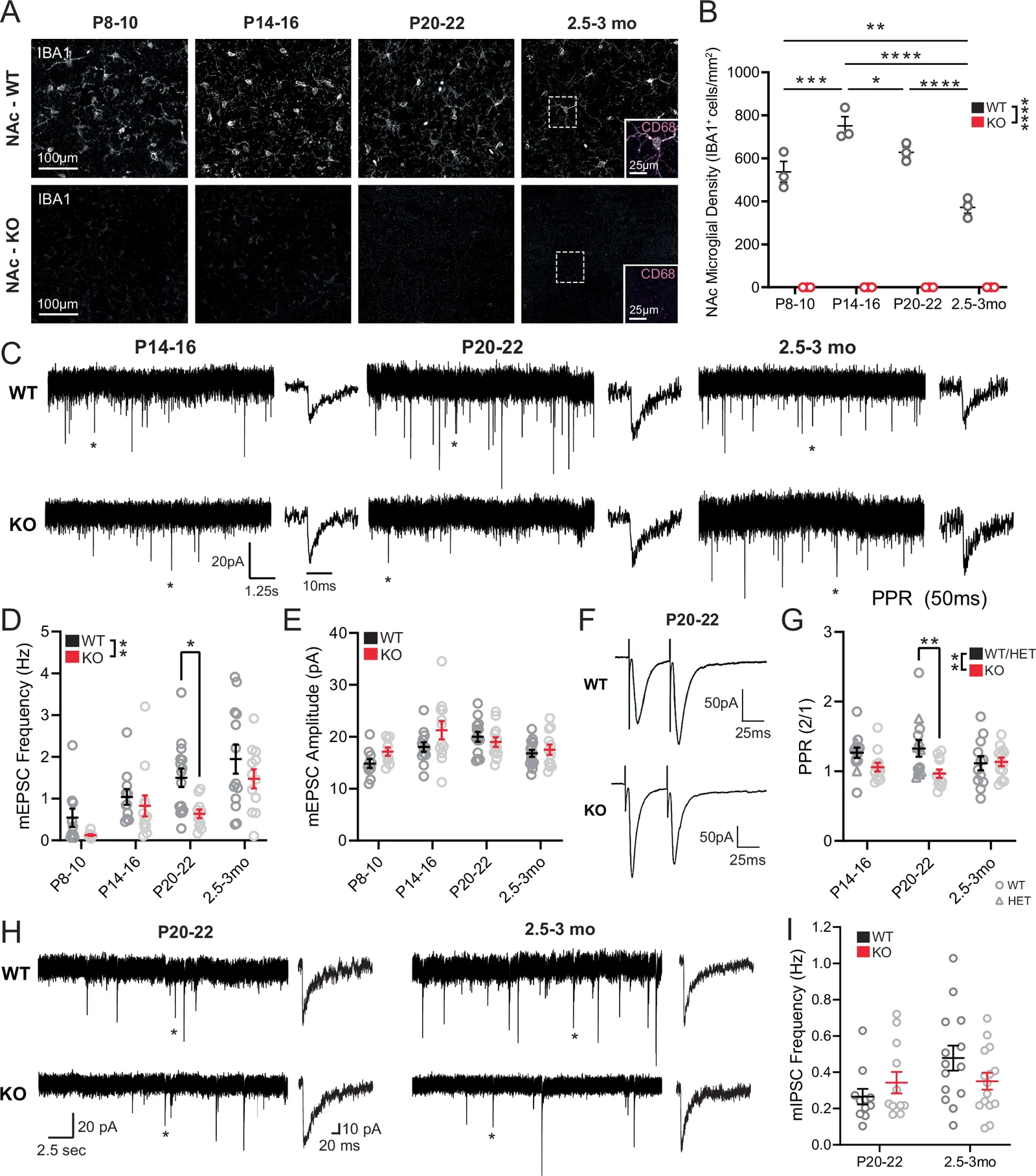
Absence of microglia blunts NAc excitatory synaptogenesis. **A** Representative images of NAc microglia in wild type (WT) and knockout (KO) mice at P8-10, P14-16, P20-22 and 2.5-3 months old. Microglia (IBA1) are represented in gray. Zoom panels represent the area with a dashed outline and also display CD68, a marker of microglial lysosomes, in magenta. Scale bar: 100 µm. **B** NAc microglial density across postnatal development in WT and KO mice. F_age_(3,16) = 18.47 *P* = 1.9 × 10^-5^, F_genotype_(1,16) = 947.0 *P* = 1×10^-14^, F_age x genotype_(3,16) = 18.47 *P* = 1.9 × 10^-5^. Two-way RM ANOVA with Šídák’s multiple comparisons test. *n* = 3 mice for each age and genotype. **C** Representative mEPSC traces in both WT and KO mice at P14-16, P20-22 and 2.5–3mo. Asterisks represent the single event shown for each trace. Cells were recorded while voltage clamping at −70 mV in the presence of 100 µM picrotoxin and 1 µM TTX. **D** mEPSC frequency in NAc of WT and KO mice across postnatal development. F_age_(3,85) = 11.01 *P* = 3.5 ×10^-6^, F_genotype_(1,85) = 8.693 *P* = 0.0041 F_age x ge__notype_(3,85) = 0.7064 *P* = 0.5508. Two-way ANOVA with Šídák’s multiple comparisons test. P8-10: WT *n* = 10 cells from 4 mice, KO *n* = 9 cells from 3 mice; P14-16: WT *n* = 11 cells from 6 mice, KO *n* = 11 cells from 5 mice; P20-22: WT *n* = 15 cells from 7 mice, KO *n* = 12 cells from 6 mice; 2.5–3mo: WT *n* = 13 cells from 7 mice, KO *n* = 12 cells from 6 mice. **E** mEPSC amplitude in NAc of WT and KO mice across postnatal development. F_age_(3,86) = 5.197 *P* = 0.0017, F_genotype_(1,86) = 3.064 *P* = 0.0836, F_age x genotype_(3,85) = 1.609 *P* = 0.1932. P8-10: WT *n* = 10 cells from 4, KO *n* = 9 cells from 3 mice; P14-16: WT *n* = 12 cells from 6 mice, KO *n* = 12 cells from 5mice; P20-22: WT *n* = 13 cells from 7 mice, KO *n* = 12 cells from 6 mice; 2.5-3mo: WT *n* = 14 cells from 7 mice, KO *n* = 12 cells from 6 mice. **F** Representative traces of evoked paired-pulse ratio (PPR) recordings with a 50 ms interstimulus interval in NAc knockout and control mice at P20-22. Cells were recorded while voltage clamping at −70 mV in the presence of 100 µM picrotoxin. **G** PPR for 50 ms interstimulus intervals in NAc of KO and control mice at P14-16, P20-22, and 2.5-3mo. F_Age_(2,69) = 0.1065, *P* = 0.8991; F_genotype_(1,69) = 7.215, *P* = 0.0090; F_age x genotype_(2,69) = 2.669, *P* = 0.0765. Two-way ANOVA with Šídák’s multiple comparisons test. P14-16: WT/HET *n* = 13 cells from 4 mice, KO *n* = 12 cells from 3 mice; P20-22: WT/HET *n* = 13 cells from 4 mice, KO *n* = 12 cells from 4 mice; 2.5-3mo: WT *n* = 12 cells from 4 mice, KO *n* = 13 cells from 4 mice. **H** Representative traces of mIPSC events in both WT and KO at P20-22 and 2.5-3mo. Asterisks represent a single event shown on the right of each trace. Cells were recorded using high-chloride internal solution while voltage clamping at −70mV in the presence of 5 µM CNQX and 1 µM TTX. **I** mIPSC frequency in NAc of WT and KO mice at P20-22 and 2.5-3mo. F_age_(1,51) = 4.452 *P* = 0.0398; F_genotype_(1,51) = 0.2903, *P* = 0.5924; F_age x genotype_(1,51) = 3.327, *P* = 0.0740. Two-way ANOVA with Šídák’s multiple comparisons test. P20-22: WT *n* = 11 cells from 5 mice, KO n = 12 cells from 5 mice; 2.5-3mo: WT *n* = 14 cells from 6 mice, KO n = 15 cells from 6 mice. *P < 0.05, **P < 0.01, ***P < 0.001, ****P < 0.0001. Error bars represent mean ± SEM. Data points shown as triangles indicate heterozygous (Csf1r^ΔFIRE/WT^) mice. Black symbols, WT mice. Red symbols, KO mice. n represents biological replicates. Source data are provided as a Source Data file on the Figshare repository.

We used a recently developed mouse line completely lacking microglia to investigate the impact of these cells on NAc synapse development. These mice were generated via deletion of fms-intronic regulatory enhancer (FIRE), a tissue-specific enhancer for colony stimulating factor 1 receptor (CSF1R), which microglia require for survival^19^. Immunostaining for microglia (Iba1/CD68) confirmed that control (*Csf1r*^+/+^, WT and *Csf1r*^ΔFIRE/+^, HET) mice from this line exhibited prominent increases in NAc microglial density in the second and third postnatal weeks followed by a decrease by 3 months of age (Fig. 1A, B and Fig. S1), consistent with our previous findings^15^. In contrast, in the NAc of *Csf1r*^ΔFIRE/ΔFIRE^ (KO) mice, no Iba1+ or CD68+ cells were observed throughout early postnatal development or adulthood (Fig. 1A, B).

To probe whether absence of microglia impacts excitatory synapse development in the NAc, we performed whole-cell patch clamp recordings from NAc medium spiny neurons in WT and KO mice at postnatal day (P)8-10, P14-16, P20-22, and 2.5-3 months (mo) of age (Fig. 1C). These time points span the infantile to early juvenile period and young adulthood and capture periods when we observe prominent overproduction of microglia^15^. In WT mice, we observed a steady increase in mEPSC frequency across developmental time points but no changes in mEPSC amplitude (Fig. 1D, E). Mice lacking microglia exhibited significantly lower mEPSC frequency across these timepoints, with this effect being most pronounced at P20-22. By adulthood, mEPSC frequencies in KO mice had increased, and did not differ significantly from WT levels. We also analyzed mEPSC kinetics at ages where these events were sufficiently abundant to allow rigorous fitting of average traces (Fig. S2A). This revealed a significantly faster mEPSC rise time in KO mice at P20-22 that remained significant in adulthood (Fig. S2B), suggesting that there may be differences in AMPA receptor subunit composition at individual excitatory synapses of KO mice. No differences in mEPSC decay were observed at any age (Fig. S2C).

A change in mEPSC frequency is typically driven by a change in either synapse number or synaptic release probability. To investigate potential changes in synaptic release probability, we performed paired-pulse electrical stimulation experiments in NAc at P14-16, P20-22, and 2.5-3 mo (Fig. 1F and S2D). No significant changes in paired-pulse ratio (PPR) were observed across ages in WT and HET control mice (Fig. 1G). However, PPR was significantly lower in KO mice compared to WT and HET controls at P20-22 (Figs. 1 and S2E), suggesting that excitatory glutamatergic inputs to MSNs in KO mice have *enhanced* synaptic release probability. Therefore, reduced mEPSC frequency at this age is more likely to be driven by a reduction in synapse number. The elevated release probability in KO mice may represent a compensatory mechanism engaged in response to reduced excitatory synaptic input.

In the barrel cortex, microglia shape AMPA receptor insertion and abundance relative to NMDA receptors during postnatal development^20^. To further examine whether the absence of microglia affected the complement of AMPA and NMDA receptors at NAc glutamatergic synapses, we performed electrical stimulation and examined the AMPA/NMDA ratio by voltage clamping neurons at either -70 mV or +40 mV in the presence of picrotoxin, a GABA receptor antagonist (Fig. S2F). The AMPA/NMDA ratios remained consistent across ages and genotypes (Fig. S2G), suggesting that microglia do not significantly impact the relative abundance of these receptors at NAc glutamatergic synapses and further highlighting region-specific roles for microglia in supporting neural development.

Although microglial impact on synaptic function has largely been studied at glutamatergic excitatory synapses, microglia have also been shown to interact with inhibitory synapses in several brain regions^6,7,21^. To determine if the effects we observed were specific to excitatory synapses, we also recorded miniature inhibitory postsynaptic currents (mIPSCs) in P20-22 and adult mice (Fig. 1H), targeting the developmental time point when the largest impact on glutamatergic synapses was detected. No significant changes in mIPSC frequency or amplitude were observed across age or genotype (Figs. 1I, S3A). We also observed no significant differences in mIPSC rise time or decay constant between WT and KO mice (Fig. S3B-D). Altogether, these findings suggest that the impact of microglial absence on NAc synaptic development is more pronounced at glutamatergic compared to GABAergic synapses.

**Fig. 2:**
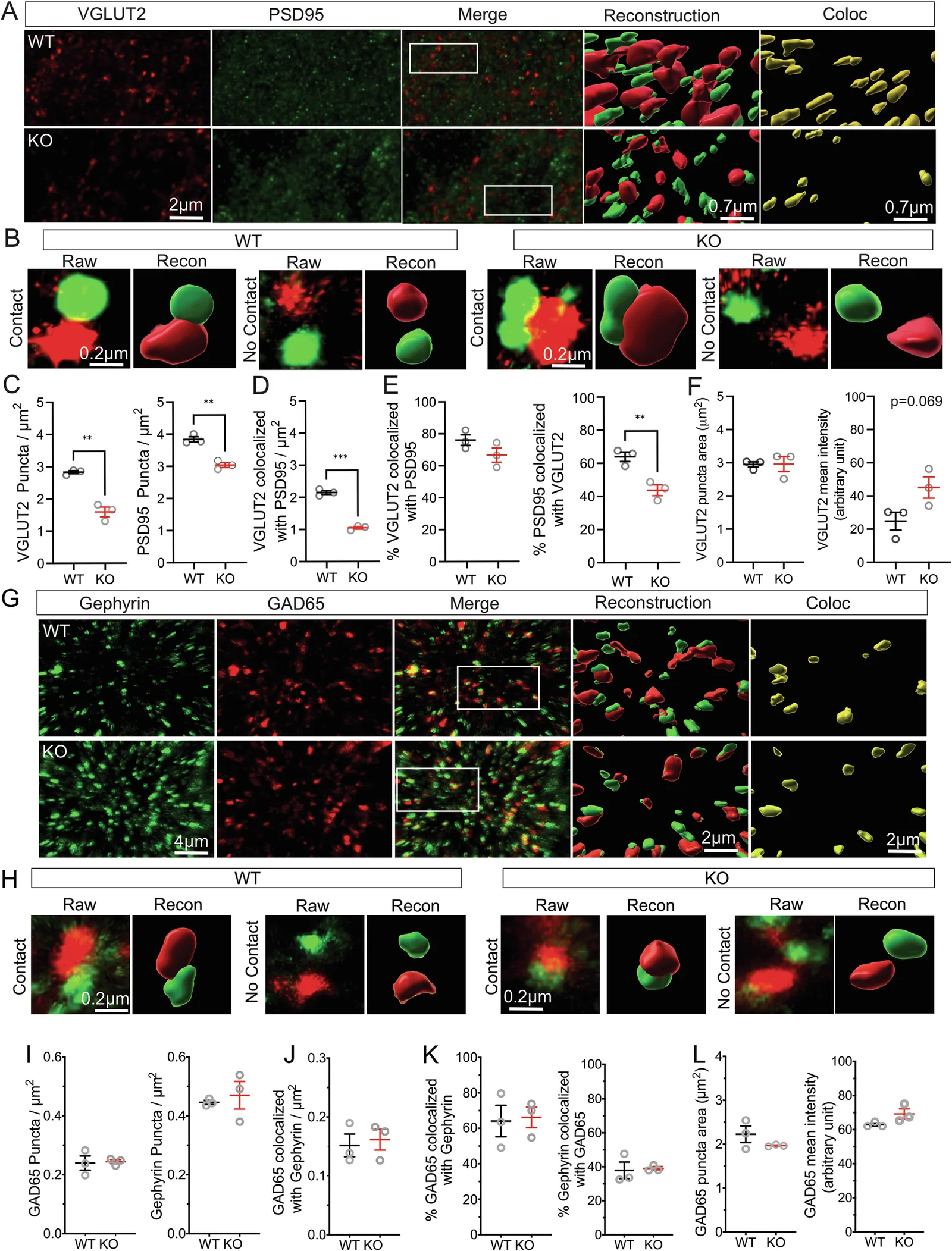
Absence of microglia blunts formation of excitatory synapses. **A** Representative images of VGLUT2, a presynaptic glutamatergic marker, and PSD95, a postsynaptic marker, in the NAc of P22 WT and KO mice. Regions highlighted by *white rectangles* are shown at higher magnification for PSD95 and VGLUT2 puncta 3D reconstructions. **B** Examples of PSD95 and VGLUT2 puncta 3D reconstructions from a P22 WT and KO mice, showing both co-localized puncta and nearby puncta that were not considered to be colocalized. **C** Quantification in the NAc of WT and KO mice at P22 of VGLUT2 and PSD95 puncta. VGLUT2: *P* = 0.0012, PSD95: *P* = 0.0017. **D** Quantification of the density of colocalized PSD95 and VGLUT2 puncta. VGLUT2: *P* = 0.0002. **E** Percentage of PSD95 and VGLUT2 puncta that were colocalized with one another. VGLUT2: *P* = 0.1624, PSD95 *P* = 0.0111. **F** Quantification of VGLUT2 puncta area and VGLUT2 puncta mean fluorescence intensity. Area: *P* = 0.9608, Intensity: *P* = 0.069, *n.s*.. **G** Representative images of GAD65, a presynaptic GABAergic marker, and Gephyrin, a postsynaptic marker, in the NAc of P22 WT and KO mice. Regions highlighted by *white rectangles* are shown at higher magnification for GAD65 and Gephyrin puncta 3D reconstructions. **H** Examples of GAD65 and Gephyrin puncta 3D reconstructions from a P22 WT and KO mouse, showing both co-localized puncta and nearby puncta that were not considered to be colocalized. **I** Quantification in the NAc of WT and KO mice at P22 of GAD65 and Gephyrin puncta. **J** Density of colocalized GAD65 and Gephyrin puncta. **K** Percentage of GAD65 and Gephyrin puncta that were colocalized with one another. **L** GAD65 puncta area and Gephyrin puncta mean fluorescence intensity. C–F and I–L: Unpaired, two-tailed *t*-test. WT: *n* = 3 mice, KO: *n* = 3 mice. **P < 0.01, ***P < 0.001, ****P < 0.0001. Error bars represent mean ± SEM. Black symbols, WT mice. Red symbols, KO mice. n represents biological replicates. Source data are provided as a Source Data file on the Figshare repository.

For an independent means to probe the impact of microglial absence on NAc synaptogenesis, we used immunohistological approaches and confocal imaging to quantify synapse number. We perfused WT and KO mice at P20-22, when the most significant reduction in mEPSC frequency was observed, and performed immunohistological staining for VGLUT2 (pre-synaptic vesicular glutamate transporter found at excitatory inputs from subcortical nuclei^22–24^) and PSD95 (key postsynaptic scaffold protein^25^)(Fig. 2A, B). High-resolution confocal microscopy and 3D reconstruction of VGLUT2+ and PSD95+ puncta revealed that KO mice had reduced densities of both synaptic markers (Fig. 2C). Density of co-localized VGLUT2 + /PSD95+ puncta was also reduced in KO mice (Fig. 2D), as was the percentage of PSD95+ puncta that colocalized with VGLUT2+ puncta, and vice versa (Fig. 2E). Approximately 40-60% of PSD95+ puncta were apposed to VGLUT2+ puncta, in line with prior reports showing comparable densities of VGLUT1+ and VGLUT2+ axon terminals in the rodent NAc^26^. While there were no significant changes in the area of individual VGLUT2+ puncta, we observed a trend toward increased staining intensity of these puncta in KO mice (Fig. 2F). We also observed a diffuse, dendritic-like PSD95 staining pattern in KO samples (Fig. S4), suggesting that PSD95 distribution may be altered in KO mice. Importantly, the diffuse nature of this staining pattern does not impact our ability to identify PSD95 puncta and quantify their abundance and colocalization with VGLUT2. Similar analysis of GAD65 and Gephyrin, pre- and postsynaptic markers of inhibitory synapses, respectively, revealed no significant changes in inhibitory synapse density, puncta colocalization, puncta area, or of GAD65 mean intensity between WT and KO mice (Fig. 2H–L). Together, these histological analyses provide further evidence that developmental NAc glutamatergic synaptogenesis is blunted in the absence of microglia.

### NAc astrocytes show increased GFAP expression but intact morphology in the absence of microglia

Microglia and astrocytes can regulate each others’ properties^27–30^, and astrocytes can promote synapse formation and maturation^5,31–33^. To explore whether the absence of microglia affects NAc synaptic function indirectly via an impact on astrocytes, we first immunostained for S100β, a calcium-binding protein expressed in astrocytes, and glial fibrillary acidic protein (GFAP), a marker of astrocyte reactivity (Fig. S5A). In other brain regions, S100β and GFAP levels increase across postnatal development^34–36^. In line with this, the density of S100β+ cells in the NAc increased across the first three postnatal weeks in both WT/Het control mice as well as KO mice (Fig. S5B). However, density of S100β+ cells was significantly higher in KO mice compared to WT/Het control mice at P20-22. In contrast to what has been reported in other brain regions, we observed a developmental *decrease* in the density of GFAP+ astrocytes in the NAc of WT/Het control mice (Fig. S5C). This decrease was less prominent in KO mice, leading to a significantly higher density of GFAP^+^ astrocytes in KO compared to control mice at both P20-22 and 2.5-3 mo.

To further assess the status of NAc astrocytes in the absence of microglia, we used a recently developed viral strategy which enables sparse labeling of astrocytes while simultaneously revealing the full architecture of their highly vellate processes^37^. We carried out minimally invasive retro-orbital injections of these astrocyte-specific viruses at P12 and collected tissue at P22 for confocal microscopy and analysis of NAc astrocyte morphology. Astrocytes were reconstructed using 3D Morph^38^. Territory (convex hull) as well as structure of major branches was compared across genotypes (Fig. S5D). No significant changes in cell territory, number of primary process branchpoints, number of primary process endpoints, or average primary process branch length between control and KO mice were observed (Fig. S5E), suggesting that NAc astrocyte morphology is preserved in mice lacking microglia. These morphological analyses align with our electrophysiological data showing that mEPSC and mIPSC decay constants were unaltered in KO mice (Fig. S2C, 3D); synapse-ensheathing astrocyte processes and their neurotransmitter re-uptake transporters contribute to decay kinetics of synaptic currents^39–41^ and any pronounced perturbation of astrocyte morphology would be expected to prolong decay kinetics. All together, these histological and electrophysiological analyses suggest that microglial absence minimally impacts astrocyte structure and synapse ensheathment, but does alter expression of proteins associated with reactivity, such as GFAP.

### NAc tissue proteomics highlights impact of microglial absence on synaptic function

**Fig. 3:**
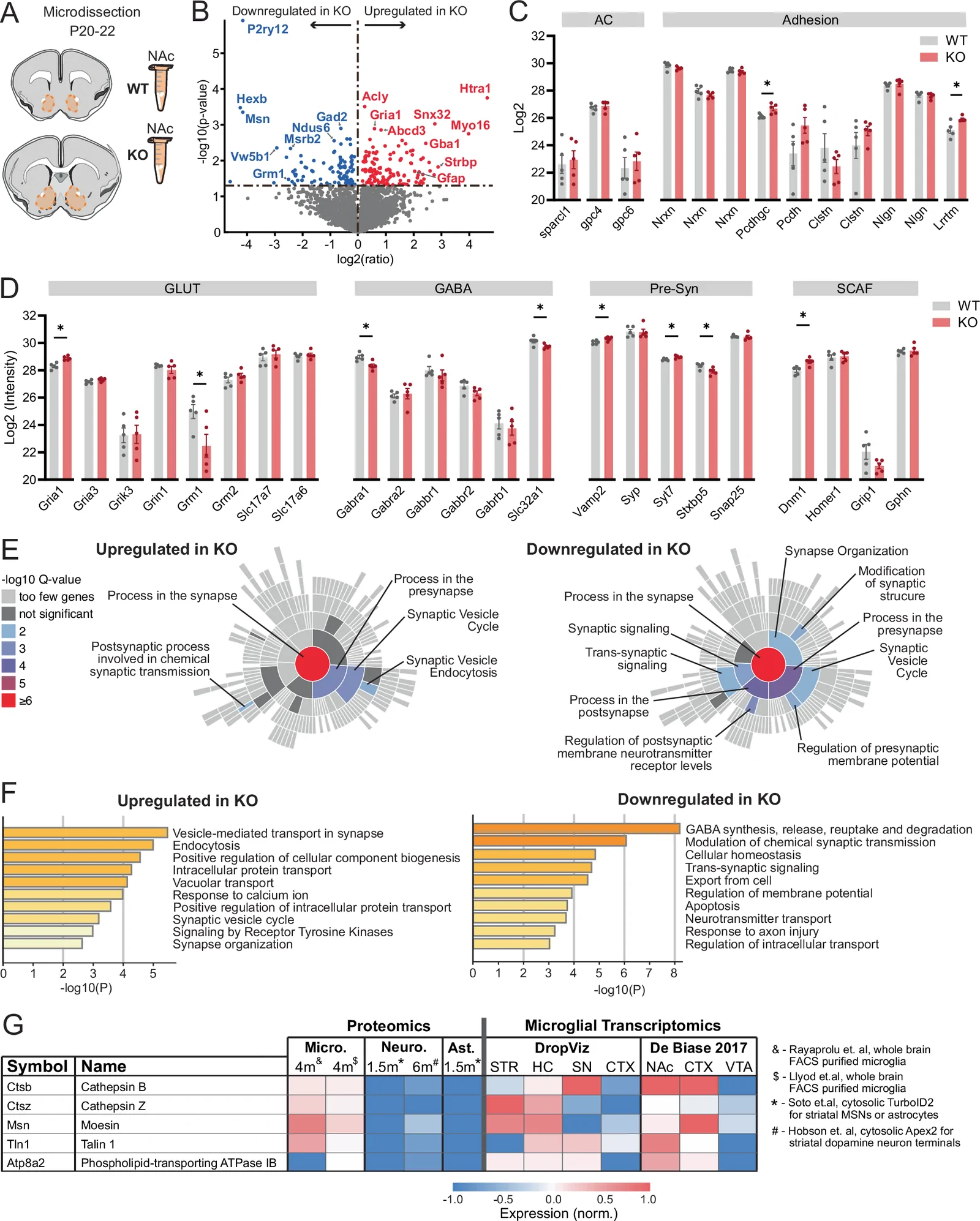
Microglia shape the developing NAc proteome. **A** Schematic representing NAc microdissection (orange) from acute brain slices at P20-22 in WT and KO mice. **B** Volcano plot showing up- and down-regulated proteins in NAc of KO mice compared to WT (*P* values represent results of two-tailed unpaired *t*-test). **C** Expression levels of proteins known to be involved in promoting synapse formation and maturation. AC: astrocyte synaptogenic proteins. (Two-tailed unpaired *t*-test, WT: *n* = 5 mice; KO: *n* = 5 mice). **D** Expression levels of subunits for major glutamate receptors (GLUT), subunits for GABA receptors (GABA), key components of pre-synaptic vesicle release machinery (Pre-Syn) and key scaffold proteins for post-synaptic neurotransmitter receptors (SCAF) (Two-tailed unpaired *t*-test, WT: *n* = 5 mice; KO: *n* = 5 mice). **E** SynGo synaptic protein enrichment analysis of up- and down-regulated proteins in KO mice compared to WT controls. **F** Metascape pathway analysis of up- and downregulated proteins in KO mice compared to WT controls, showing top pathways relevant for molecular, cellular, or circuit-level information. Complete list of pathways is available in Figure S6 and Supplementary Data 2. **G** Table representing potential gene candidates extracted from published proteomic and RNA sequencing datasets. Error bars represent mean ± SEM. *P < 0.05. Grey bars, WT mice. Red bars, KO mice. n represents biological replicates. Source data are provided as a Source Data file on the Figshare repository.

Although astrocyte morphology appears intact in KO mice, microglial absence could perturb astrocyte secreted molecules that promote synapse formation and/or cell adhesion proteins that play key roles in synaptogenesis, thus reducing the overall number of synapses that are formed. To explore this possibility, we performed whole-tissue LC-MS/MS proteomic analysis on microdissected NAc from WT and KO mice at P20-22 (Fig. 3A), when reduced mEPSC frequency is most prominent in KO mice and reduced excitatory synapse density was observed via histology. We detected more than 3,700 proteins, 229 ( ~ 10%) of which differed significantly across genotype (Fig. S6A and Supplementary Data 1).

Differentially expressed proteins included, as expected, prominently downregulated microglia-specific (P2ry12, Hexb) and microglial-enriched (Ctsb, Ctsz, Npl, CyfiP1) proteins^42^ in tissue from KO mice (Fig. 3B). Moreover, proteins that are highly expressed by microglia in microglial-specific proteomic datasets (Myh9, Msn, Cap1, Ywhae, Lmnb1, Aldoa, Mdh1)^43,44^ were also significantly downregulated in tissue from KO mice (Supplementary Data 1), despite microglia only comprising roughly 5% of cells in the NAc. Proteins significantly upregulated in NAc tissue from KO mice included GFAP, supporting histological observations that this astrocyte marker is elevated in the absence of microglia. However, astrocyte-derived synaptogenic cues that were detected (Sparcl1, Gpc4, Gpc6) did not differ significantly across genotypes (Fig. 3C). Well-characterized astrocyte proteins that shape glutamate clearance and potassium buffering at the synapse, including Slc1a2 (GLT1, glutamate transporter), Slc1a3 (GLAST, glutamate transporter), and Kcnj10 (KIR4.1, potassium channel), were also unaltered in KO mice, supporting electrophysiological observations that mEPSC and mIPSC decay kinetics did not differ across genotypes. For a more unbiased evaluation of astrocyte-relevant proteins in our dataset, we also examined proteins identified by recent proteomic studies that profiled cytosolic proteins of striatal astrocytes, as well as proteins enriched in their synapse-ensheathing processes^45^. The majority (>90%) of these proteins did not differ in abundance between KO and WT mice in our NAc tissue proteomics dataset (Supplementary Data 1, and Fig. S5B,C)^45^. Together with preserved astrocyte morphology (Fig. S5) and mEPSC/mIPSC kinetics (Figures S2,S3), these data suggest that astrocyte structure and capacity to regulate basal synapse function in the NAc are largely intact in the absence of microglia. Nonetheless, it will be important for future studies to examine astrocyte synapse ensheathment at the ultrastructural level, to use cell-specific (rather than tissue level) proteomic approaches to examine astrocytes in KO mice, and to probe synaptic responses during high frequency stimulation and plasticity induction protocols.

Families of adhesion molecules, such as neurexins, neuroligins, and protocadherins also play key roles in promoting synapse formation/maturation^46–48^. However, detected proteins within these families also did not differ significantly across genotype (Fig. 3C). These observations suggest that the reduced number of functional excitatory synapses observed in KO mice via electrophysiology cannot be explained by insufficient abundance of astrocytic and adhesive proteins that promote synaptogenesis. Indeed, adhesion molecules Pcdhgc5 and Lrrtm3 were significantly *up*regulated in KO mice along with Snx32, a trafficking protein that regulates neurite outgrowth^49^, and Myo16, a myosin that regulates actin cytoskeleton reorganization^50^ (Fig. 3B). Upregulation of these proteins may represent compensatory responses engaged by reduced synapse number.

A reduced number of functional excitatory synapses could, theoretically, also be driven by insufficient availability of essential protein components (i.e. the “building blocks”) necessary to assemble functional synapses. To determine if abundance of such proteins was reduced by microglial absence, we examined glutamate and GABA receptor subunits, key postsynaptic scaffold proteins, and central components of pre-synaptic vesicle fusion machinery. Abundance of the majority of detected proteins in these categories did not differ across genotypes (Fig. 3D). Proteins in these categories that *did* differ between genotypes were evenly split between proteins that were up-regulated in KO mice (4 proteins) and those that were down-regulated in KO mice (4 proteins). Altogether, these targeted analyses suggest that reduced formation of glutamatergic synapses in KO mice is not due to insufficient protein-level synthesis of glutamate receptor subunits, scaffolding proteins needed to anchor glutamate receptors at the plasma membrane, or machinery needed to mediate release of glutamate containing synaptic vesicles. However, synapses consist of more than 1500 proteins, and their formation, maturation, and function is determined by much more than neurotransmitter receptors, key individual scaffold proteins, and core vesicle release components.

To gain more comprehensive insight into the status of synapse-relevant proteins, we also interrogated differentially expressed proteins from our tissue proteomic dataset with SynGo^51^ (Fig. 3E), which probes for enrichment of 1,100+ proteins that impact synapse function. This analysis indicated that microglial absence altered numerous synapse-relevant proteins, including proteins with roles in synapse structure and both pre- and post-synaptic functioning. Moreover, general pathway analysis of differentially expressed proteins using Metascape^52^ revealed alterations in multiple pathways associated with synaptic function among both up- and down-regulated proteins, including synaptic vesicle recycling, neurotransmitter transport, and synapse organization (Fig. 3F, S6E-F, and Supplementary Data 2). “GABA synthesis, release, reuptake, and degradation” was a top hit on Metascape analysis of downregulated proteins, suggesting that, although GABAergic synapse number was normal via electrophysiological and histological experiments in KO mice (Figs. 1, 2), these synapses may exhibit functional deficits under conditions of heightened activity. Altogether, SynGo and Metascape analyses support the idea that, despite minimal changes in the abundance of neurotransmitter receptors, key scaffolding proteins, and core synaptic vesicle release proteins, synapses are a prominent tissue compartment impacted by microglial absence in the developing NAc.

The fact that astrocytic and adhesive synaptogenic molecules were not significantly decreased by microglial absence, raises the possibility that microglia directly promote NAc synapse formation or stabilization via cell surface or secreted molecules. To explore this possibility, we screened our proteomics dataset for proteins that were significantly downregulated in the absence of microglia and used functional annotation to further select proteins that are secreted or expressed on plasma membranes. Finally, we leveraged publicly available RNAseq^42,53^ and proteomic datasets^43–45,54^ to determine whether members of this list were expressed at the protein level by microglia, whether expression is enriched in microglia compared to other CNS cells, and how microglial expression of these molecules in striatum / NAc compares to that in other brain regions. This analysis pipeline identified multiple candidate molecules that microglia could use to promote excitatory synapse formation at this age (Fig. 3G) and that warrant follow-up examination in future studies. Identified candidate molecules included secreted degradative enzymes Cathepsin B and Cathepsin Z, that could degrade extracellular matrix to permit synapse formation^55–57^, as well as Moesin, which regulates immunological synapse formation^58^, among other candidates.

### Absence of microglia perturbs avoidance behavior

**Fig. 4:**
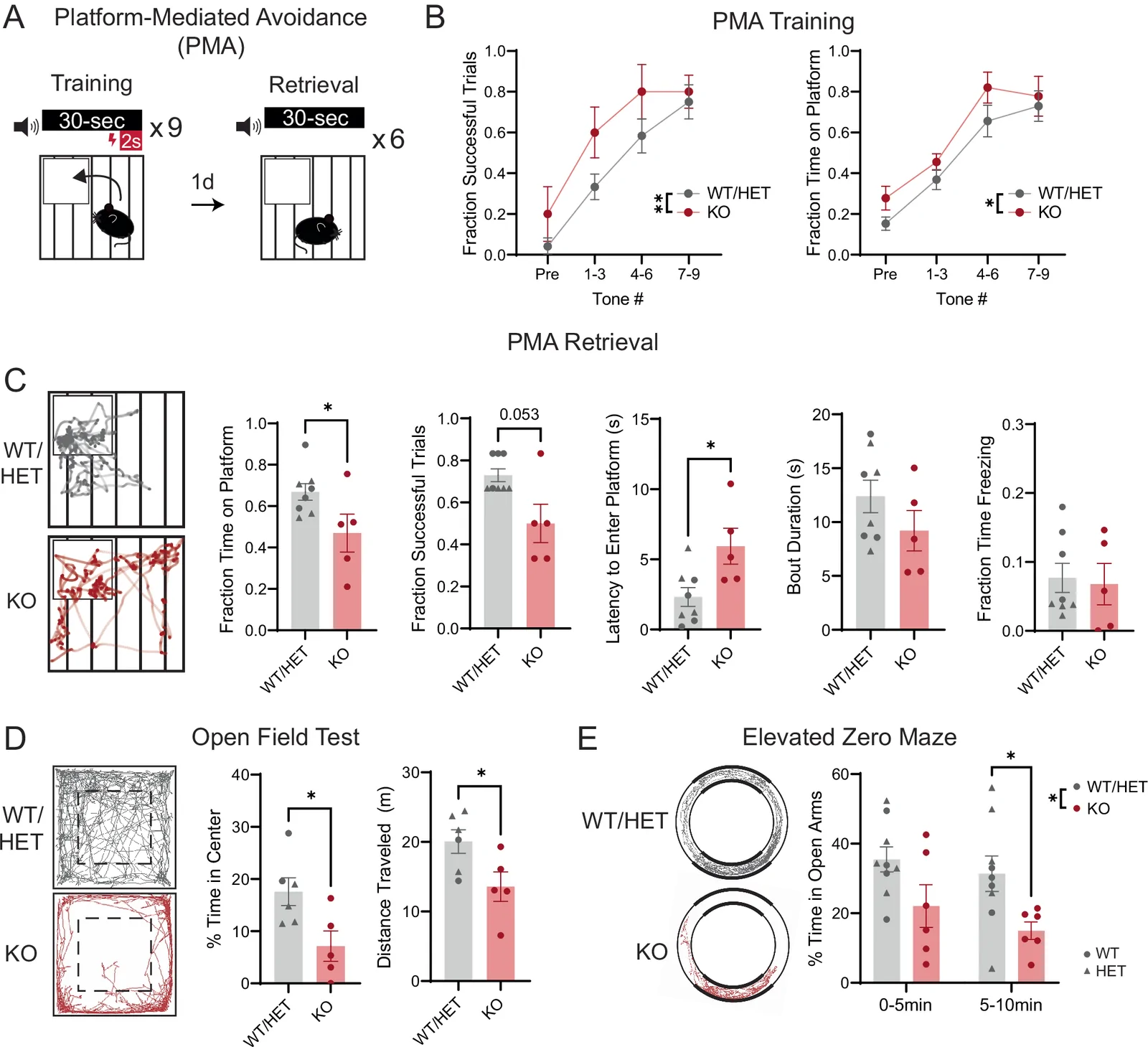
Absence of microglia disrupts learned and innate avoidance behavior in adulthood. **A** Schematic of behavioral paradigm. Mice received 9 tone-shock pairings on training day followed by 6 tones without shock on retrieval day. **B** PMA Training: Left, number of successful trials (i.e. successful avoidance of a foot shock) across training. F_Tone #_(3,33) = 18.01, 4.2 x 10^-7^; F_Genotype_(1,11) = 12.48, *P* = 0.0047; F_Tone # x Genotype_(3,33) = 0.4567, *P* = 0.7143, Two-way RM ANOVA. Right, fraction of time spent on the safety platform during tone periods across training. F_Tone #_(3,33) = 29.40, *P* = 1.9 × 10^-9^; F_Genotype_(1,11) = 4.905, *P* = 0.0488; F_Tone # x Genotype_(3,33) = 0.2694, *P* = 0.8470, Two-way RM ANOVA. WT/HET: *n* = 8 mice, KO: *n* = 5 mice. **C** Left, trajectory plots indicating the path of representative WT and KO mice during tone periods of PMA retrieval. Right, time on platform, successful trials, latency to enter the platform, platform bout duration, and freezing behavior during tone periods of PMA retrieval. Successful trials: two-tailed Mann-Whitney U test (*P* = 0.0528); Others: two-tailed unpaired student’s *t-*test (time on platform: *P* = 0.0437, latency: *P* = 0.0187, bout duration: *P* = 0.2140, freezing: *P* = 0.8028). WT/HET: *n* = 8 mice, KO: *n* = 5 mice. **D** Behavior of KO and control mice in the open field test, including % time in the center of the arena and total distance traveled. Two-tailed unpaired student’s *t*-test (time in center: *P* = 0.0265, distance traveled: *P* = 0.0386). WT/HET: *n* = 6 mice, KO: *n* = 5 mice. **E** Behavior of KO and control mice in the elevated zero maze. Two-way RM ANOVA with Sidak’s multiple comparisons test. F_Time_(1,13) = 1.952, *P* = 0.1857; F_Genotype_(1,13) = 7.848, *P* = 0.0150; F_Time x Genotype_(1,13) = 0.1400, *P* = 0.7143. WT/HET: *n* = 9 mice, KO: *n* = 6 mice. *P < 0.05, **P < 0.01. Error bars represent mean ± SEM. Data points shown as triangles indicate heterozygous (Csf1r^ΔFIRE/WT^) mice. Grey symbols, WT mice. Red symbols, KO mice. n represents biological replicates. Source data are provided as a Source Data file on the Figshare repository..

Although electrophysiological measures of NAc synapse number were largely normalized by adulthood in mice lacking microglia, these changes could, nonetheless, produce lasting effects on behaviors that engage NAc circuits, such as threat avoidance behaviors^59–61^. To test this, we trained adult KO and WT/HET control mice in platform-mediated avoidance (PMA)^59,62^ (Fig. 4A). In PMA, mice learn to associate a tone with a foot shock and that they can avoid impending shocks by escaping onto a safety platform. Both KO and control mice learned to avoid nearly all shocks by the end of the training session, but KO mice learned at a faster rate (Fig. 4B). During a retrieval test one day later, tones were presented without shocks to examine memory-guided threat avoidance behavior (Fig. 4C). When the tone played, control mice entered the safety platform with short latency and remained there during the final 2 seconds of the tone (when the shock would have occurred) on the majority of trials (Fig. 4C). Surprisingly, KO mice displayed decreased levels of avoidance, marked by a longer latency to enter the safety platform, decreased time on the platform during tone periods, and fewer successful trials (Fig. 4C). We did not observe any differences in freezing behavior (Fig. 4C), suggesting that differences in avoidance behavior did not arise from differences in the fearful association between the tone and the shock.

To determine if differences in PMA behavior were driven by differences in anxiety-like behaviors, we compared control and KO mouse behaviors in the open field test (OFT) and the elevated zero maze (EZM). Mice innately avoid the center of an open field arena and the open arms of the EZM, as these are associated with higher levels of potential threat exposure. KO mice spent significantly less time in the center and travelled less distance in the OFT, and also spent significantly less time in the open arms of the EZM (Fig. 4D,E). Hence, KO mice exhibit prominent anxiety-like phenotypes, suggesting that the decreased avoidance during PMA retrieval is driven by differences in learning and memory and not by lower levels of innate avoidance. We also compared sensitivity to the foot shock stimulus across control and KO mice. KO mice generally had a higher shock threshold to elicit behavioral responses (Fig. S7A), but a scurry response was observed in all mice well below 0.13 mA, the shock intensity used for PMA, suggesting that differences in shock sensitivity do not explain the behavioral differences we observed in PMA.

### Absence of microglia alters NAc activity during PMA

**Fig. 5:**
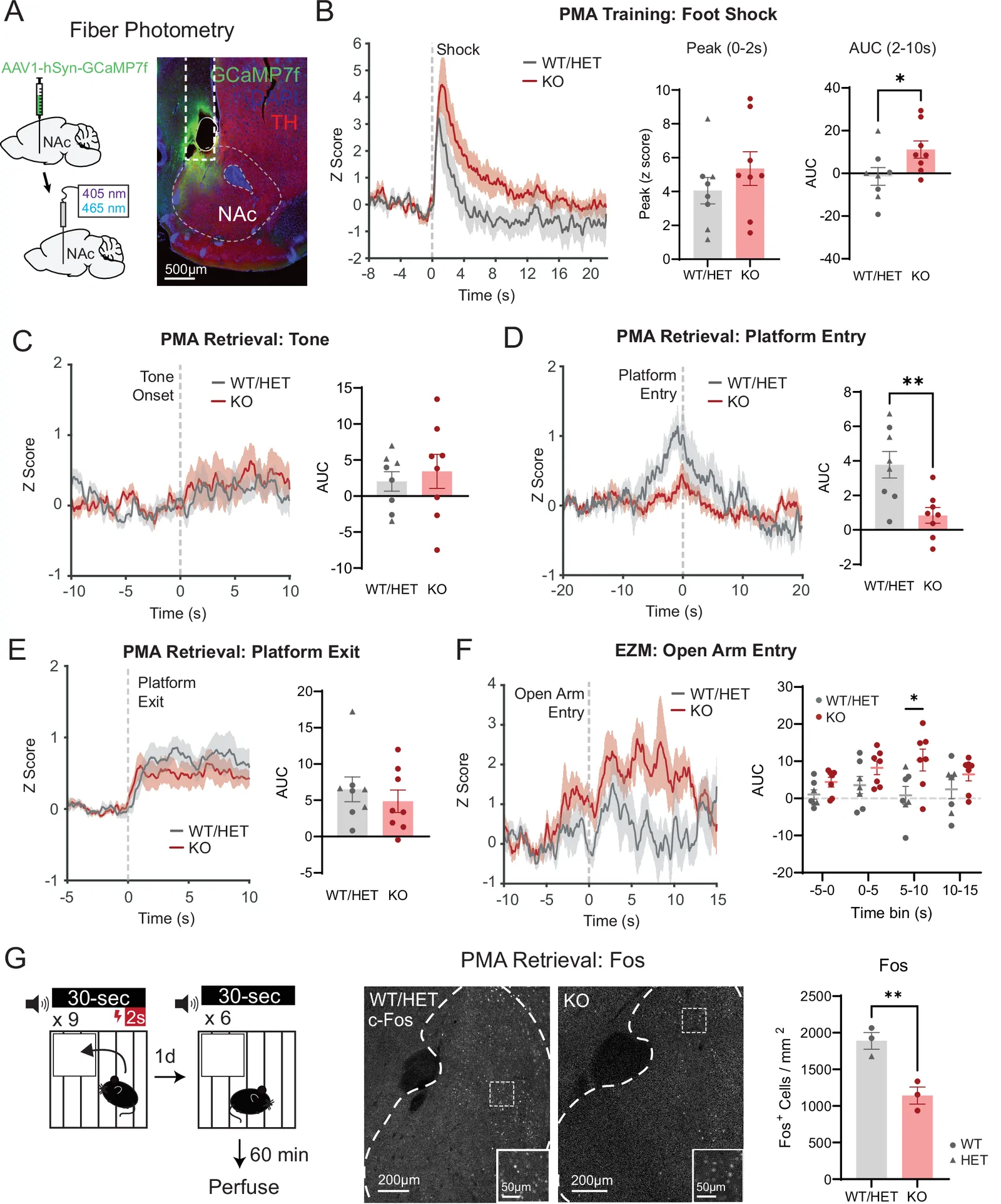
Absence of microglia alters in vivo function of the adult NAc during learned avoidance. **A** Injection strategy (left) and representative histological image (right) demonstrating viral expression and fiber placement in NAc. Scale bar 500 µm. **B** Left, average traces representing foot-shock elicited NAc activity during PMA training in knockout and control mice. Error shading represents mean ± SEM by animal. Middle, peak shock response during the first two seconds following shock onset. Right, area under the curve (AUC) following the shock period. Two-tailed unpaired student’s *t*-test (peak: *P* = 0.3192, AUC: *P* = 0.0476). WT/HET: *n* = 8 mice, KO: *n* = 8 mice. **C** NAc activity at tone onset during the retrieval session (AUC: 0 to 10 s). Two-tailed unpaired student’s t-test (*P* = 0.6192). WT/HET: *n* = 8 mice, KO: *n* = 8 mice. **D** NAc activity surrounding platform entry during the retrieval session (AUC: -5 to 0 seconds). Two-tailed unpaired student’s *t*-test (*P* = 0.0052). WT/HET: *n* = 8 mice, KO: *n* = 8 mice. **E** NAc activity following platform exit during the retrieval session (AUC: 0 to 10 s). Two-tailed unpaired student’s *t*-test (*P* = 0.4906). WT/HET: *n* = 8 mice, KO: *n* = 8 mice. **F** NAc activity surrounding open arm entries in the elevated zero maze. Two-way RM ANOVA with Sidak’s multiple comparisons test. F_Time_(3,36) = 3.330, *P* = 0.0302; F_Genotype_(1,12) = 4.047, *P* = 0.0673; F_Time x Genotype_(3,36) = 3.202, *P* = 0.0346. WT/HET: *n* = 7 mice, KO: *n* = 7 mice. **G** c-Fos^+^ neuron density in NAc following PMA in knockout and control mice. Two-tailed unpaired student’s *t-*test (*P* = 0.0099). WT/HET: *n* = 3 mice, KO: *n* = 3 mice. Scale bars 200 µm. *P < 0.05, **P < 0.01. Error bars represent mean ± SEM. Data points shown as triangles indicate heterozygous (Csf1r^ΔFIRE/WT^) mice. Grey symbols, WT mice. Red symbols, KO mice. *n* represents biological replicates. Source data are provided as a Source Data file on the Figshare repository..

KO mice lack microglia throughout the CNS and microglial absence could impact neuronal function in multiple brain regions involved in successful PMA learning and memory. To probe whether changes in threat avoidance behavior specifically involve changes in NAc neuronal activity in KO mice, we used fiber photometry to record fluorescence activity from a virally-delivered, genetically encoded calcium sensor (AAV-syn-jGCaMP7f-WPRE) expressed in NAc neurons while mice performed PMA (Fig. 5A). During training, foot shocks induced a brief, sharp increase in NAc activity in WT/HET control mice that rapidly decayed below the pre-shock baseline (Fig. 5B). KO mice exhibited a similar peak compared to control mice, but NAc activity remained elevated well after the shock, resulting in a significantly higher area under the curve (AUC) for the 20 s post-shock period (Fig. 5B). During the retrieval test, we observed no notable fluctuations in NAc activity at the onset of the tone in both control and KO mice, consistent with our previous reports^63^ (Fig. 5C). During platform entries however, we observed an increase in NAc activity in control mice that was absent in KO mice (Fig. 5D). During platform exits, control and KO mice exhibited similar increases in activity (Fig. 5E). We also recorded NAc activity during the EZM, and we observed increased activity in KO mice following entry into the open arms (Fig. 5F), consistent with the increased anxiety-like behavior we had observed in this assay (Fig. 4J). As an independent means of probing NAc activity levels in relation to behavior, we perfused mice 90 minutes after completion of PMA retrieval and immunostained for Fos, a marker of recently active neurons. KO mice had significantly fewer Fos+ NAc neurons compared to control mice (Fig. 5G), further demonstrating that the observed behavioral effects during PMA retrieval are associated with decreased NAc activity.

### Transient developmental depletion of NAc microglia alters adult behaviors

**Fig. 6:**
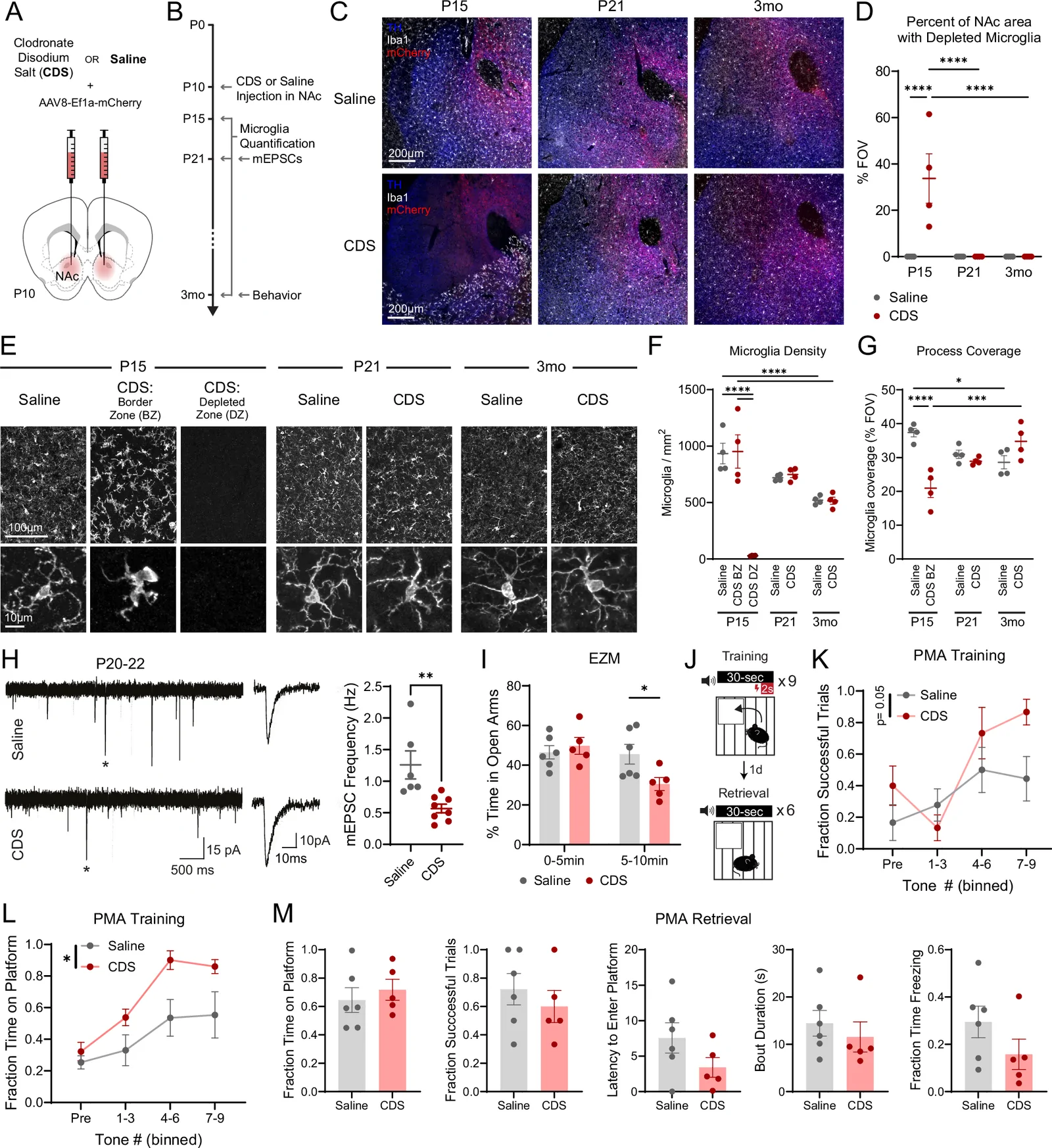
Depletion of NAc microglia during the second postnatal week results in lasting effects on avoidance behavior. **A** Injection strategy. **B** Experiment timeline. **C** Representative images of Iba+ NAc microglia in saline or CDS-infused mice aged postnatal day (P)15, P21, or adult (3 months). **D** Quantification of percent NAc area with depleted microglia. Two way ANOVA with Tukey’s multiple comparisons test. F_age_(2,18) = 10.04, *P* = 0.0012, F_CDS_(1,18) = 10.04, *P* = 0.0053, F_age x CDS_(2,18) = 10.04, *P* = 0.0012. Saline: *n* = 4 mice per age group, CDS: *n* = 4 mice per age group. **E** Representative images of Iba+ NAc microglia in depleted and border zones at P15, P21, and adult (3 mo) stages. **F** Quantification of microglia density in saline and CDS groups, with separate groups for depleted and border zones in CDS group. Two way ANOVA with Sidak’s multiple comparisons test. F_age_(2,23) = 21.34, *P* = 5.7 × 10^-6^, F_CDS_(2,23) = 65.94, *P* = 3.0×10^-10^. Saline: *n* = 4 mice per age group, CDS: *n* = 4 mice per age group. **G** Quantification of microglia process coverage. Two-way ANOVA with Tukey’s multiple comparisons test. F_age_(2,18) = 0.9492, *P* = 0.4056, F_CDS_(1,18) = 7.104, *P* = 0.0158, F_age x CDS_(2,18) = 18.50, *P* = 4.3×10^-5^. Saline: *n* = 4 mice per age group, CDS: *n* = 4 mice per age group. **H** Representative traces of mEPSCs recorded from MSNs in both saline and CDS treated mice at P20-22. Asterisks represent the single event shown for each trace. mEPSC frequency shown *at right*. Two-tailed unpaired t test (*P* = 0.0058). Saline: *n* = 6 cells from 3 mice, CDS: *n* = 8 cells from 3 mice. **I** Behavior of saline- and CDS-treated mice in the elevated zero maze. Two-way RM ANOVA with Sidak’s multiple comparisons test. F_Time_(1,9) = 7.982, *P* = 0.0199; F_CDS_(1,9) = 1.663, *P* = 0.2293; F_Time x CDS_(1,9) = 6.519, *P* = 0.0310. Saline: n = 6 mice, CDS: *n* = 5 mice. **J** Schematic of PMA behavioral paradigm. Mice received 9 tone-shock pairings on training day followed by 6 tones without shock on retrieval day. **K** PMA Training: number of successful trials (i.e., successful avoidance of a foot shock) across training. Two-way RM ANOVA. F_Tone #_(3,27) = 6.564, *P* = 0.0018; F_CDS_(1,9) = 5.094, *P* = 0.0504; F_Tone # x CDS_(3,27) = 1.769, *P* = 0.1768. Saline: *n* = 6 mice, CDS: *n* = 5 mice. **L** PMA training: fraction of time spent on the safety platform during tone periods. Two-way RM ANOVA. F_Tone #_(3,27) = 15.10, *P* = 5.8×10^-6^; F_CDS_(1,9) = 7.437, *P* = 0.0233; F_Tone # x CDS_(3,27) = 1.426, *P* = 0.2568. Saline: *n* = 6 mice, CDS: *n* = 5 mice. **M** Behavior during PMA retrieval. Successful trials: two-tailed Mann-Whitney test (*P* = 0.5130). Others: two-tailed unpaired student’s *t-*test (time on platform: *P* = 0.5536, latency: *P* = 0.1574, bout duration: *P* = 0.5045, freezing: *P* = 0.1780). Saline: *n* = 6 mice, CDS: *n* = 5 mice. *n* represents biological replicates. Source data are provided as a Source Data file on the Figshare repository..

To determine to what extent the behavior changes we observed arise from the absence of NAc microglia during development, we used clodronate disodium salt (CDS)^64–67^ to transiently deplete NAc microglia during the second postnatal week in WT mice (Fig. 6A) and assess the effects on adult behavior (Fig. 6B). We stereotactically injected CDS or saline into the NAc at P10, as microglial depletion is strongest 5 days following CDS injection^64–67^, and P15 aligns with the peak window of microglial overproduction in NAc^15^ (Fig. 1A). Immunostaining for Iba1 at P15, P21, and 3 mo timepoints revealed large microglia-depleted zones (DZ) in the NAc of CDS-treated mice at P15. Microglia were again fully tiling the NAc by P21 and we observed no DZs at P21 or 3 mo timepoints (Fig. 6C, D). Compared to microglia farther away from the DZ, those at the DZ border at P15 showed increased expression of Iba1 and simplified morphology (Fig. 6C). Microglia in these border zones (BZ) in CDS treated mice had similar density to that of NAc microglia in control mice (Fig. 6F). However, microglia in BZ had reduced tissue coverage (Fig. 6G), confirming a distinct microglial morphology compared to control mice. These changes in tissue coverage were no longer present at P21 or in adulthood (Fig. 6F, G), suggesting the primary effects on microglia were specific to the second postnatal week. Moreover, we observed no effects on the density of GFAP^+^ astrocytes or NeuN^+^ neurons in CDS-treated mice at P15 (Fig. S8A, B), indicating that this perturbation primarily impacts microglia, as previously described^64–66^.

To investigate the effects of this transient microglial depletion on synaptic function, we recorded mEPSCs from NAc in acute brain slices from CDS- or saline-injected mice at P20-22. In line with our observations in *Csf1r*^ΔFIRE/ΔFIRE^ KO mice, frequency of mEPSCs in CDS-treated mice was significantly reduced at this age (Fig. 6H). No significant changes were observed in mEPSC amplitude, rise time, or decay (Figure S8C-F). This supports our previous observation that depletion of microglia impairs NAc excitatory synaptogenesis.

We next examined the effects of transient developmental depletion of NAc microglia on adult threat avoidance behaviors. Similar to the *Csf1r*^ΔFIRE/ΔFIRE^ KO mice (Fig. 4D,E), CDS-treated mice exhibited anxiety-like behaviors in the elevated zero maze (Fig. 6I) and increased avoidance levels during PMA training (Fig. 6J-L). However, CDS-treated mice did not exhibit changes in PMA retrieval (Fig. 6M), suggesting that the memory deficits we observed in *Csf1r*^ΔFIRE/ΔFIRE^ KO mice (Fig. 4C) may reflect the acute absence of NAc microglia in the adult brain, their absence during a different developmental window, or microglial absence in another brain region. Together, these results provide strong support for a causal link between developmental microglial-neuron interactions in the NAc and the shaping of adult anxiety and avoidance phenotypes.

## Discussion

Here we used the *Csf1r*^ΔFIRE/ΔFIRE^ mouse line to study how the absence of microglia impacts the functional maturation of the NAc. Patch clamp studies provided insight into the normative developmental trajectory of NAc synapse development, and, together with histological analyses, revealed that KO mice had blunted excitatory synaptogenesis in juvenile stages of development (Figs. 1, 2). NAc tissue proteomic analysis supported the idea that changes to synapse status is a primary tissue-level effect of lacking microglia in the developing NAc. Proteomic and histological analyses also indicated that astrocyte morphology, detected astrocyte-derived synaptogenic cues, and adhesion proteins that promote synapse maturation, were unchanged in the absence of microglia (Fig. 3). Using a combination of behavioral assays and fiber photometry, we found that in adult KO mice, disruptions in threat avoidance behavior were associated with altered patterns of NAc activity (Figs. 4, 5). Transient depletion of NAc microglia during the second postnatal week produced similar blunted synaptogenesis during development as well as behavioral disruptions in adults (Fig. 6). Together, our findings revealed that the absence of microglia led to altered NAc synapse development and lasting changes in NAc circuit activity and related behaviors. Moreover, we highlight candidate proteins that may mediate microglial support of NAc synaptogenesis and that merit dedicated follow-up functional studies.

Although microglial removal of synapses via phagocytic engulfment has received the vast majority of attention when investigating synaptic interactions of these cells^68,69^, microglia also promote synapse formation and synapse maturation^7–9^. In the developing cortex (P8-10), microglia can induce dendritic spine formation by contacting neuronal dendrites and inducing local dendritic Ca^2+^ transients and actin remodeling^8^. Microglial depletion also reduces dendritic spines on immature, adult born granule cells in the olfactory bulb^70^, further supporting links between microglia and formation of post-synaptic structures at excitatory synapses. Microglia may play similar roles at inhibitory synapses; they promote formation of GABAergic axo-axonal synapses between chandelier cells and pyramidal neurons in cortex and these synapses are reduced by microglial depletion, microglial responses to LPS, or deletion of microglial GABA_B1_ receptors^7^. Perturbing microglial maturation via deletion of Cx3cr1 (fractalkine receptor) also indicates roles for microglia in synapse development, with Cx3cr1 KO mice displaying deficient AMPA receptor insertion and synapse maturation in early postnatal barrel cortex^20^ and reduced spine density in adult born granule cells in the olfactory bulb^70^. Despite multiple examples of microglial influence on synapse formation, microglial involvement in synaptogenesis has not been investigated in most brain regions and the molecular mechanisms underlying these microglial actions remain obscure.

Our results suggest that microglia influence multiple aspects of synaptic function in the developing NAc. In the absence of microglia, we observed blunted NAc excitatory synaptogenesis throughout the first three postnatal weeks, with effects being most prominent in the third postnatal week (Figs. 1, 2). We also observed a dendritic-like PSD95 staining pattern to varying degrees in KO animals that was not observed in WT controls, which may reflect changes in PSD95 trafficking or localization. The first three postnatal weeks are a highly active period of synaptogenesis in dorsal striatum^17,18^, and our results in WT mice similarly show increasing mEPSC frequency in the NAc throughout this period. Given our previous finding that NAc microglia are overproduced in the second and third postnatal week^15^, and the deficits in NAc synaptogenesis in KO mice, these findings raise the possibility that striatal microglia are specifically overproduced during this developmental window to support synapse formation.

Our NAc tissue proteomic analyses supported the idea that synapses are a subcellular compartment impacted by microglial absence. Pathway analyses highlighted impacts on both pre- and post-synaptic compartments (Fig. 3E, F). We observed upregulation of GluA1 in KO mice, which could lead to an increased number of GluA2-lacking AMPA receptors in KO mice. These receptors have faster rise times and greater inwardly rectifying currents^71,72^, which aligns with our observation of faster mEPSC rise times in both P22 and adult mice (Fig. S2B). Future studies should investigate the effects of microglial absence on AMPA receptor rectification to provide further insight into the impact on glutamate receptor subunit composition. We observed a significant reduction in expression of the GABA_A_ alpha-1 receptor subunit, Gabra1, for which changes in expression can occur independently of changes in inhibitory synapse number. GABA synthesis enzymes (GAD1, GAD2) and GABA transporters (e.g. the vesicular GABA transporter VGAT) were also down-regulated in KO mice, leading “GABA synthesis, release, reuptake and degradation” to be the top hit in pathway analysis of significantly down-regulated proteins. Decreases in these proteins could impact the capacity for sustained high frequency synaptic activity, but would not necessarily impact the *number* of functional GABA synapses. Hence, these findings are not inconsistent with our observation that mIPSC frequency was similar in WT and KO mice (Fig. 1I), but they point to the need for future study of GABAergic synapses under high activity demand in the absence of microglia.

Our proteomics data also indicated that the reduced number of glutamatergic NAc synapses in P22 KO mice was not accompanied by reductions in detected astrocyte derived synaptogenic cues or adhesive proteins that support synapse formation (Fig. 3C and S5C). This raises the possibility that microglia directly support NAc glutamatergic synaptogenesis and absence of this microglial support blunts synapse number. Further mining of our proteomic data pointed to the degradative enzymes Cathepsin B and Cathepsin Z as potential secreted microglial molecules that could regulate synapse formation. These cysteine proteases are highly expressed by microglia, including those in the dorsal striatum and NAc (Fig. 3G). Moreover, secreted cathepsins could support formation of additional synapses via degradation of extracellular matrix (ECM) and/or activation of growth factors such as BDNF^56,57^. Indeed, in other studies, we found that adult *Csf1r*^ΔFIRE/ΔFIRE^ KO mice have elevated ECM accumulation in the NAc compared to control mice^73^, supporting the idea of microglial-based ECM regulation in this brain region. Microglial proteins that could play a role in contact-based promotion of synapse formation include Moesin and Talin-1. These molecules are also highly expressed by microglia (Fig. 3G) and play key roles in formation of cell-cell focal adhesions and cell-surface localization of integrins^58,74^, which are highly implicated in synapse formation^75^. Cell-specific manipulation of these molecules in future studies can test whether they play a role in microglial support of synapse formation in the developing NAc.

One limitation of whole-tissue proteomics is that these data cannot reveal whether some synapse-relevant proteins with normal abundance nonetheless have perturbed protein trafficking. Our proteomics pathway analysis indicated multiple hits related to intracellular protein trafficking (Fig. 3), suggesting that approaches to investigate protein trafficking, particularly within neuronal dendrites and axons, should be a key future direction. This idea is further reinforced by the diffuse, dendritic-like PSD95 staining patterns we observed in KO mice (Fig. S4). Also, it is possible that decreased protein expression in one cell type could be masked by increased expression in another cell type. Future studies using cell-specific and/or cell compartment specific proteomics can be used to further explore the molecular mechanisms of how microglial absence impacts synapse development. Astrocytes also regulate synapse formation and function^5,31–33^ and we observed increases in multiple astrocyte proteins (Gfap, Htra1, S1pr1) in KO mice (Fig. 3 and S5), which may be driven by lack of microglia-derived regulatory cues or represent a compensatory mechanism. Although we did not observe changes in detected astrocyte-derived synaptogenic cues (Gpc4, Gpc6, Sparcl1), some astrocyte-derived synaptogenic cues were not detected via proteomics (e.g. Tsp4, Thbs1, Thbs2, Sparc, Chrdl1) and we cannot exclude that these factors are present in the developing NAc at low levels and are perturbed by microglial absence. Moreover, S1pr1, which we found to be upregulated in KO mice, is expressed in perisynaptic astrocyte processes and regulates astrocyte morphology^76,77^. In neuron-astrocyte co-cultures, activation of S1pr1 increased astrocyte expression of synaptogenic cues such as Sparcl1 and Tsp4^76^, an effect that required neuron-astrocyte contact. Hence, additional studies will be needed to fully define the status of astrocyte support for synaptogenesis in the absence of microglia.

Many of the effects we observed on functional synapse maturation in KO mice normalized to near-WT levels by adulthood, suggesting that compensatory synaptogenic mechanisms are engaged in other cell types between P22 and adulthood. Another possibility is that the juvenile-specific synaptic changes we observed may represent a developmental delay induced by chronic microglial absence, with ongoing synaptogenic mechanisms eventually able to reach near-WT adult levels. However, this eventual normalization of synapse number does not necessarily mean that specific patterns of circuit connectivity and circuit function are comparable across genotypes. Indeed, we observed lasting effects of microglial absence on NAc circuit function. Both adult *Csf1r*^ΔFIRE/ΔFIRE^ KO mice and adult wildtype mice that underwent transient NAc microglial depletion during the second postnatal week exhibited anxiety-like behaviors and enhanced avoidance learning (Figs. 4, 6). Combined with the synaptic and molecular changes observed at P22, these findings indicate that the synapse-promoting functions of NAc microglia during this developmental window, or changes in the maturation trajectory of NAc, are important for establishing circuits that underlie threat learning and anxiety-related behaviors in adulthood. Only *Csf1r*^ΔFIRE/ΔFIRE^ KO mice showed a memory retrieval impairment, which was accompanied by reduced behavior-associated NAc activity (Figs. 4, 5). Previous studies have found that adult-specific loss of microglia enhances remote fear memory without affecting recent fear memories^78–81^, suggesting that the impairment we observed may arise either from the absence of NAc microglia during a different development window than the one we targeted for transient depletion, or from microglia loss in other brain regions. Given that studies of microglia contributions to associative learning are limited and have yielded conflicting findings^79^, alternative explanations should be considered. One possibility is that acute microglial absence in adulthood disrupts normal mechanisms for synaptic plasticity and memory consolidation, consistent with evidence that microglia in the adult motor cortex contribute to motor learning by promoting synapse formation^9^. Finally, microglia also regulate neuronal excitability^82^ and our proteomics pathway analysis showed changes in membrane potential-regulating proteins (Fig. 2). Thus, lasting changes in neuronal excitability could also contribute to the observed changes in NAc activity in adult *Csf1r*^ΔFIRE/ΔFIRE^ KO mice (Fig. 5). Additional studies are needed to investigate these possibilities.

Our findings contrast with recent work showing normal synapse development in the hippocampus and barrel cortex of *Csf1r*^ΔFIRE/ΔFIRE^ KO mice^83^. O’Keefe and colleagues observed minimal differences in transcriptome of P14 cortical neurons and astrocytes in the absence of microglia, which aligns with our findings that the majority of astrocyte-enriched proteins detected via NAc tissue proteomics did not differ between P22 *Csf1r*^ΔFIRE/ΔFIRE^ KO mice and controls (Fig. 3, S5, Supplementary Data 1). However, O’Keefe and colleagues recorded normal mEPSC frequency in the hippocampus at P14 and P28, developmental periods when we observed the most significant decreases in NAc mEPSC frequency in *Csf1r*^ΔFIRE/ΔFIRE^ KO mice relative to controls (Fig. 1). O’Keefe and colleagues measured normal coherence of hippocampal and cortical oscillatory activity in adult *Csf1r*^ΔFIRE/ΔFIRE^ KO mice, whereas we found significant anxiety-like phenotypes, impaired avoidance learning, and perturbed behavior-associated activity patterns in the NAc of adult *Csf1r*^ΔFIRE/ΔFIRE^ KO mice (Figs. 4, 5). These divergent findings strongly support the theory that microglia play region-specific roles in synaptic development and refinement. Moreover, they may highlight critical regional differences in capacity of other CNS cells to compensate for perturbations in microglial function. Critically, microglia have been implicated in the etiology of psychiatric disorders, as they are sensitive to environmental risk factors such as chronic stress, severe early life infections, and pollution and toxin exposure^84^. There is also evidence that microglia mitigate the effects of chronic stress on neural circuit function and behavior^84,85^ and perturbed microglial function increases vulnerability to anxiety-like behaviors^86,87^. Our findings, together with those of O’Keefe et al., suggest that brain regions that are particularly relevant for psychiatric illness, such as the mesolimbic system, may be uniquely vulnerable to perturbed circuit development as a result of insults that impact microglial function.

Genetically-induced absence of microglia provides a valuable tool to easily study the effects of microglia on brain development, as microglia never form at any point throughout the lifespan. Moreover, this approach has advantages over drug-, toxin-, and CreER/tamoxifen-induced depletion, which could lead to off-target effects induced by administered substances as well as the death and clearance from the tissue of the existing microglia. However, the *Csf1r*^ΔFIRE/ΔFIRE^ microglial depletion model lacks the temporal specificity required to reveal how microglia may be changing their functional roles at different stages of development. In addition, both genetic and drug-induced approaches lack regional selectivity and eliminate microglia across the entire brain. We leveraged stereotaxic delivery of clodronate disodium salt to achieve transient and brain region specific depletion of microglia in the NAc. Although this approach carries its own caveats (invasive stereotaxic injection, potential effects of clodronate on vasculature and other glial cells), the combined results from these two approaches to microglial depletion strongly support a role for NAc microglia in shaping developmental NAc synaptogenesis and circuit maturation. As tools improve to induce focal elimination of select populations of microglia, it will be important to compare the roles of local microglia within the NAc with the roles of microglia in long-range input and output structures. These studies will be crucial as we continue to identify region and subregion-specific differences in microglial phenotype and function^15,53,88^.

## Methods

All procedures in this study followed animal care guidelines approved by the University of California, Los Angeles Chancellor’s Animal Research Committee under protocol ARC-2018-103 (De Biase) and ARC-2019-12 (DeNardo).

### Animals

Male and female mice were used for all experiments. *Csf1r*^ΔFIRE/WT^ mice^19^ were obtained from the Pridans lab at the University of Edinburgh and crossed with wild-type CBA mice (Jackson Laboratories #000656). Resulting *Csf1r*^ΔFIRE/WT^ mice were crossed to produce *Csf1r*^ΔFIRE/ΔFIRE^ (KO), *Csf1r*^ΔFIRE/WT^ (HET), and *Csf1r*^WT/WT^ (WT) littermates. For some experiments, HET mice were included in the microglia-positive control group, as these mice have no detectable difference in microglia number compared to WT controls^19^. For transient microglial depletion experiments, mice on a C57BL/6 background were used (Jackson Laboratories #000664). Mice were housed on a 12-hour light cycle (lights on 6am-6pm).

### Retro-orbital viral injections

To sparsely label astrocytes in mice, we performed intravenous administrations of PHPeB::GfaABC1D-Cre-4x6T, PHPeB::CAG-flex-lck-smV5-4x6T, and PHPeB::CAG-flex-GFP-4x6T via retroorbital injections, as previously described^37^. Briefly, the viruses were diluted in sterile saline (0.9% NaCl), obtaining a standard solution volume of 50 μl per mouse. This viral solution was loaded into an insulin syringe with a 27 G needle. Each mouse was anesthetized with isoflurane before the needle was inserted into the medial canthus of the animal’s right eye, at a 45-degree angle behind the eye. We then dispensed 50 μl of viral solution into the retroorbital sinus of the mouse, systemically delivering PHPeB::GfaABC1D-Cre-4x6T at a titer of 5*10^10^ vg/mouse, PHPeB::CAG-flex-lck-smV5-4x6T at 2.5*10^9^ vg/mouse, and PHPeB::CAG-flex-GFP-4x6T at 2.5*10^9^ vg/mouse.

### Clodronate sodium microglial depletion

For CDS experiments, 2 parts clodronate disodium salt (Millipore Sigma, dissolved in H_2_O at 100 mg/mL) was mixed with 1 part saline (0.9% NaCl) and 1 part AAV8-Ef1a-mCherry-IRES-Cre (Addgene #55632) to achieve a final CDS concentration of 50 mg/mL, consistent with previous reports^64,65,67^. For saline controls, 3 parts saline (0.9% NaCl) was mixed with 1 part AAV8-Ef1a-mCherry-IRES-Cre. mCherry virus was included in both groups to allow for permanent visualization of injection targeting at timepoints after microglial repopulation. 250 nL of either solution was infused bilaterally into NAc of P10 mice using previously described experimental procedures for surgery at P9-10^63^. Briefly, injections followed the same procedure as above, but pups were mounted on a NeuroStar stereotaxic device, and bregma and lambda coordinates were used to scale the injection coordinates to the size of the pup’s skull. The injection coordinate used for NAc was AP + 1.3 mm, ML + /-1 mm, DV -4.65 mm. Viral targeting for all mice was confirmed by visualizing mCherry fluorescence in NAc using a Leica Scanning Microscope.

### Immunohistochemistry

Mice were anesthetized with isoflurane and transcardially perfused with phosphate-buffered saline (PBS) followed by 4% paraformaldehyde (PFA). For c-Fos experiments, mice were perfused 60 min after the end of behavioral analyses. Brains were post-fixed in PFA for 4–24 h and transferred to PBS + 0.01% sodium azide (PBS/NaN_3_). 60 µm brain sections were prepared on a vibratome or cryostat. Sections were washed 3 x 10 min in PBS and transferred to a blocking solution (2% NDS, 3% BSA, 0.3% Triton-X in PBS) for 2 h. Sections were incubated overnight at 4 °C in blocking solution containing primary antibody (Guinea Pig anti-PSD95 (Synaptic Systems #124308, 1:800), Mouse anti-VGLUT2 (DSHB N29/29, 1:150), Rat anti-Gephyrin (Synaptic Systems #147208, 1:500), Mouse anti-GAD6 (DSHB GAD-6, 1:100), Rabbit Anti-GFAP (Agilent #Z0334, 1:500), Chicken Anti-GFP (Aves #GFP1010, 1:2000); Mouse Anti-S100β (Sigma-Aldrich #S2532, 1:500), Mouse Anti-NeuN (EMD Millipore #MAB377, 1:500), Chicken Anti-TH (Aves #TYH, 1:500), Rabbit Anti-V5 (Bethyl #A190-120A, 1:1000), Rabbit Anti-c-Fos (Synaptic Systems #226008, 1:1000)), or 3-4 nights for c-Fos and V5 staining experiments. After 3 x 10 min washes in PBS, sections were incubated in PBS containing secondary antibody (Alexa 488 Donkey Anti-Rabbit (Jackson ImmunoResearch #711-545-152), Anti-Chicken (#703-545-155), Anti-Rat (#712-545-150) or Anti-Guinea Pig (#706-545-148) Alexa 594 Donkey Anti-Chicken (#703-585-155), Alexa 647 Donkey Anti-Mouse (#715-605-151), all 1:500), 2% NDS, and 3% BSA for 2 h at room temperature. Sections were then washed in PBS, stained with DAPI (1:4000), and mounted using Fluoromount G.

### Image acquisition and analysis

For analysis of microglia, astrocyte, and neuron density, images containing NAc were obtained at 20x using a Zeiss Axio Imager 2 fitted with Apotome 2, or at 20x on a Leica Stellaris Confocal microscope. S100β, GFAP, or NeuN images were collected as z-stacks with a 0.75 μm interval, and 20x IBA1 images were collected as z-stacks with a 1.5 μm interval. IBA1+ microglia, S100B+ or GFAP+ astrocytes, or NeuN+ nuclei were manually counted in two ROIs per 20x image, and each data point represents the average cell density in NAc across 2-3 brain sections per animal. For analysis of c-Fos, images containing NAc were acquired at 5x on a Leica DM6 B scanning microscope. Fos+ neurons were detected using our deep-learning based cell-detection algorithm DeepCOUNT^89^, with minor modifications for use in 2D histological sections.

For analysis of astrocyte morphology, 63x confocal stacks were collected on a Leica Stellaris confocal microscope with a 1.5 μm z interval. Astrocyte morphologies were examined using 3DMorph, a MATLAB-based, open access, tool for semi-automated reconstruction and quantification of 3-dimensional cell morphology^38^. Confocal images containing V5- or GFP-labeled astrocytes underwent a background subtraction (rolling ball radius = 30 pixels), a top-hot filter (radius = 20 pixels), and a gamma correction prior to analysis. These preprocessed images were loaded into MATLAB and thresholded manually using the standard settings in 3DMorph. Astrocyte volumes, territories (convex hull), and skeletons were extracted and used for quantification. End point and branch length quantifications were performed using the ‘Only Major Processes’ setting due to difficulty in reliably reconstructing fine distal tips across all astrocytes. Example skeletons provided in Fig. S4 were derived using the ‘Keep Small Branches’ setting.

For analysis of pre- and post-synaptic markers, images were collected as z-stacks with a 0.3μm interval. 2 z-stacks from 2 brain sections were imaged per mouse (total of 4 z-stacks per mouse). Excitatory (PSD95 and VGLUT2) and inhibitory (GAD65 and Gephyrin) synaptic puncta were reconstructed using the surfaces tool within Imaris 3D (version 10.1) software. Within each confocal stack, images were cropped to generate two regions of interest (ROIs, one 45x45 μm and one 80x80 μm placed in non-adjacent regions of the stack) that exclude ‘voids’ containing neuron somas. Consistent thresholds for puncta intensity and size were applied to all images within Imaris for surface reconstruction. Colocalized VGLUT2 + PSD95+ puncta were identified by filtering the VGLUT2 surfaces based on signal intensity within the PSD95 channel and vice versa. Similar colocalization filtering was used for GAD65 and Gephyrin puncta. Surfaces underwent minimal manual editing to delete surfaces corresponding to dim and diffuse background signal.

For CDS experiments, we first calculated the percent of the NAc field of view, as defined by a TH stain, which had little or no microglia by drawing ROIs of this “depleted zone” as well as the total NAc within ImageJ. Microglial density within depleted and border zones adjacent to depleted zones was calculated as above. Microglial coverage was calculated as previously described^15^ by z-projecting ROIs that did not contain a depleted zone. The average minimum pixel value of the dimmest microglial processes was used to threshold the image, and the percentage of pixels with intensity above this threshold was used to calculate process coverage for each mouse.

### Acute brain slice preparation

Mice were anesthetized with isoflurane. Heads were removed and placed in ice-cold NMDG cutting solution, consisting of (in mM) 92 NMDG, 20 HEPES, 30 NaHCO_3_, 1.2 NaH_2_PO_4_, 2.5 KCl, 5 Na-Ascorbate, 3 Na-Pyruvate, 2 Thiourea, 10 MgSO_4_, 0.5 CaCl_2_, 25 Glucose (adjusted to pH 7.4 with HCl). Brains were rapidly dissected and 230 μm sections containing NAc were obtained using a Leica VT1200S Vibratome in NMDG solution. Brain sections were transferred to NMDG solution at 34 °C and incubated for approximately 5 minutes prior to transfer to room-temperature ACSF consisting of 125 NaCl, 2.5 KCl, 1.25 NaH_2_PO_4_, 1 MgCl_2_, 26 NaHCO_3_, 11 Glucose, 2.4 CaCl_2_. Sections were allowed to recover for at least 45 min prior to recording.

### Slice electrophysiology

NAc neurons were visualized under infrared-differential interference contrast optics. NAc was identified by the presence of the anterior commissure. All recordings were performed at room temperature. Data were collected in pClamp 10 or 11 using an Axon Instruments Multiclamp 700B amplifier and Digidata 1440 A or 1550B digitizer. Series resistance was regularly monitored, was left uncompensated, and did not change by more than 20% across a recording. For measurements of excitatory currents, whole-cell voltage clamp recordings were obtained using borosilicate glass pipettes (3–7mOhm) filled with internal solution consisting of 117 CsMS, 20 HEPES, 0.4 EGTA, 2.8 NaCl, 5 TEA-Cl, 4 Na_2_-ATP, and 0.4 Na-GTP, adjusted to pH 7.3 using CsOH (280-290 mOsm). Miniature excitatory postsynaptic currents (mEPSCs) were obtained at -70mV. Cells were patched in ACSF containing 100 μM picrotoxin, then 1 μM tetrodotoxin was washed in prior to mEPSC recordings. Data were analyzed off-line using Clampfit (Molecular Devices), Origin (OriginLab), Mini analysis (Synaptosoft), and PRISM (GraphPad) software. Input resistance and membrane capacitance were calculated from a 2 mV hyperpolarizing step from a holding potential of −70 mV. mEPSC frequency (>5 pA amplitude, <1 ms rise time) was quantified by monitoring activity during continuous recording for at least 8 min. For measurements of miniature inhibitory postsynaptic currents (mIPSC), a high-chloride internal solution was used consisting of 105 CsCl2, 10 EGTA (CsOH), 20 TEA-Cl, 20 Hepes, 1 MgCl_2_, 2 Na_2_-ATP and 0.2 Na-GTP, adjusted to a pH at 7.3 (290 mOsm). Cells were patched in ACSF, then 1 μM tetrodotoxin and 5 μM CNQX was washed in prior to mIPSC recordings. Data were analyzed off-line using Clampfit (Molecular Devices), Origin (OriginLab), Mini analysis (Synaptosoft), and PRISM (GraphPad) software. Input resistance and membrane capacitance were calculated from a 2 mV hyperpolarizing step from a holding potential of −70 mV. mIPSC frequency (>5 pA amplitude, <1 ms rise time) was quantified by monitoring activity during continuous recording for at least 8 min.

Recordings involving electrical stimulation were obtained by positioning a bipolar stimulating electrode in NAc 100-200 μm dorsal to the cell of interest. Recordings were performed in ACSF containing 100 μM picrotoxin. Traces represent the average of 10 sweeps per neuron, with 10 seconds separating each sweep. Paired-pulse ratio (PPR) recordings were obtained by delivering two 1 ms pulses (15-100 uA) through the stimulating electrode at intervals of 20, 50, 100, and 200 ms. AMPA/NMDA ratio recordings were obtained by delivering a 1 ms electrical stimulation while holding the neuron at -70mV to obtain the AMPA current, then slowly increasing the holding potential to 40 mV and performing the same stimulation to obtain the NMDA current. The value for the NMDA current was taken as the value 50 ms after the delivery of the stimulation, as the contribution of AMPA receptors to the current at this point is minimal due to differences in channel kinetics.

PPR and AMPA/NMDA recordings were quantified in python. All traces from a cell were averaged, and the peak current value from this trace was calculated after subtracting the holding current. PPR was calculated as the ratio between the peaks of the second and first stimulations. AMPA/NMDA ratio was calculated as the ratio between the peak of the AMPA current at -70mV and the current value 50 ms after the stimulation at 40 mV.

### Tissue proteomics sample preparation

*Csf1r*^ΔFIRE/ΔFIRE^ and *Csf1r*^WT/WT^ mice age P20-22 were anesthetized with isoflurane and perfused transcardially with 10 mL of oxygenated, ice-cold NMDG solution (described above for preparation of acute brain slices for electrophysiology recordings). Brains were then rapidly dissected and coronal forebrain sections (300 µm thick) were prepared using a vibratome in ice-cold NMDG solution bubbled continuously with 95% O_2_/5% CO_2_. After sectioning, slices remained in ice-cold solution, oxygenated NMDG solution and were transferred one at a time to a glass dissecting surface under a stereoscope maintained at 4 °C. NAc were rapidly microdissected, minced, and transferred to eppendorf tubes containing 1 mL of 1 M PBS. Samples were centrifuged at 1500 xg, supernatant was removed, and tissues were frozen and stored at -80 °C until further processing.

50-100ug protein per sample was processed for mass-spectrometry analysis. Samples were resuspended in equal volume of 8 M Urea and 100 mM Tris-Cl (pH 8). This was followed by reduction with TCEP and alkylation with IAA, followed by protein clean up with SP3 beads. Proteolytic digestion was performed overnight using trypsin and lysC enzymes. Peptides were subsequently cleaned using the P3 protocol^90^, followed by elution in 2% DMSO. Samples were dried using a speed vacuum, and the dried peptides were resuspended in 5% formic acid before being sent for liquid chromatography with tandem mass spectrometry (LC-MS/MS).

### LC-MS/MS parameters and database search

For quantitative proteomic analysis of P22 NAc from WT (*n* = 5 control mice) and FIRE KO mice (*n* = 5)(Fig. 3 and Figure S5), LC-MS/MS was carried out as detailed in Jami-Alahmadi et al. 2021^91^. Approximately 200–500 ng peptide amounts were subjected to LC-MS/MS analysis. Briefly, peptide separation was carried out using reversed-phase chromatography on a 75 μm inner diameter fritted fused silica capillary column, which was packed in-house to a length of 25 cm with 1.9 μm ReproSil-Pur C18-AQ beads (120 Å pore size). An increasing gradient of acetonitrile was delivered using a Dionex Ultimate 3000 nano-LC system (Thermo Scientific) at a constant flow rate of 200 nL/min. Tandem mass spectra (MS/MS) were acquired in data-dependent acquisition (DDA) mode on an Orbitrap Fusion Lumos Tribrid mass spectrometer (Thermo Fisher Scientific). Full MS1 scans were acquired at a resolution of 120,000, followed by MS2 scans at a resolution of 15,000. The raw LC-MS/MS data were analyzed using the MaxQuant computational platform^92^. The Andromeda search engine, integrated within Max Quant, was employed for peptide identification against the UniProt reference proteome for *Mus musculus* (UP000000589). Search parameters allowed a maximum of two missed tryptic cleavages and included cysteine carbamidomethylation as a fixed modification and N-termination acetylation and methionine oxidation as variable modifications. A false discovery rate (FDR) threshold of 1% was applied at both peptide and protein levels. Label-free quantification (LFQ) was enabled, with a minimum LFQ ratio count set to one. Precursor ion and fragment ion mass tolerances were set at 20 ppm and 4.5 ppm, respectively. The resulting MaxQuant output files were subsequently used for statistical analysis to identify differentially enriched proteins.

Raw intensity data from each sample was batch corrected using a quantile normalization followed by a median centering procedure. This procedure uses an iterative algorithm to find the point that minimizes Euclidean distance to all features in the dataset and adjusts the distribution from each batch to this point. The intensity data of each identified protein were log2 normalized for analysis. To be included in the analysis, a protein needed to be detected in 75% or more of the samples for each genotype.

Fold changes were calculated as log_2_ of the ratio of the KO signal relative to the WT signal. Statistical significance was assessed using unpaired t-tests with an alpha-level of 0.05. Process enrichment analysis of the proteins up-and down- regulated was conducted in Metascape^52^. Synaptic protein enrichment analysis was conducted using SynGo^51^. To examine astrocyte-enriched proteins in our dataset, we utilized protein lists from Soto et al. 2023^45^. In this study, viral approaches were used to express BioID2 in striatal astrocyte cytosol or to target BioID2 to specific striatal astrocyte subcellular compartments in young adult mice. The authors then treated mice with biotin and streptavidin purified biotinylated proteins from striatal tissues. We utilized lists of cytosolic astrocyte proteins enriched relative to neurons and GLT1 subcompartment proteins enriched relative to cytosol or other compartments, which were provided in supplementary spreadsheets, and we examined the expression levels of these proteins in KO vs WT samples in our NAc proteomic dataset.

### Platform-mediated avoidance

All behavior experiments were performed on adult mice between 2.5 and 4 months of age. Mice were handled for at least 10 minutes across 2-3 days prior to all behavioral experiments. For platform-mediated avoidance (PMA), mice were placed in an operant chamber with a shock floor. A quarter of the floor was covered by a plexiglass platform which prevented transmission of the shock. Two scented odor pods containing peanut butter or almond, coconut, or vanilla extract were placed beneath the shock floor to promote exploration. Following 80 s to explore the chamber, mice received 3 baseline tones (4 kHz, 75 dB, 30 s) separated by a randomized interval of 80–150 s. Mice then received 9 tone-shock pairings, with each tone co-terminating with a 0.13 mA, 2 s foot shock. The following day, mice were placed in the same chamber and received 6 tones with no shocks. Videos of behavior were obtained using PointGray Chameleon 3 cameras (Teledyne FLIR) and analyzed using DeepLabCut^93^ and BehaviorDEPOT^62^ as previously described.

### Open field test & elevated zero maze

For open field behavior, mice were placed in an open arena (50 x 50 cm) with 30 cm walls, and behavior was recorded for 10 min. The center was defined as a 30 x 30 cm square in the middle of the arena. For elevated zero maze, mice were placed on a custom built apparatus 24 inches in diameter, and behavior was recorded for 10 min. These assays were analyzed using Biobserve Viewer software.

### Shock sensitivity

Mice were placed in an operant chamber with a shock floor. Mice received a 2 s foot shock every 20 s, starting at a 0.02 mA intensity and increasing in increments of 0.02 mA until a vocalization response was elicited, defined as the mouse emitting an audible sound. A blinded experimenter documented the first instance of scurry, dart, and vocalization for each mouse. Scurry was defined as a slow, backward movement with shuffling of the paws. Dart was defined as a more rapid forward movement at some point during the shock period.

### Stereotaxic surgery

Mice were anesthetized in 5% isoflurane until loss of righting reflex, and the hair was cleared from the scalp using a small razor. Mice were transferred to a stereotaxic surgical apparatus and received 2% isoflurane to maintain anesthesia. Body temperature was maintained at 40 °C using a water-based heating system. The scalp was cleaned with 3 alternating swabs of betadine and 70% ethanol, and a small incision was made in the scalp over the region of interest. Carprofen (50 mg/kg) was delivered subcutaneously. A small hole was drilled over the injection site, and a hamilton syringe was used to deliver 400nL of AAV-syn-jGCaMP7f-WPRE (1.80*10^13^ vg/mL, diluted 1:1 in 0.9% NaCl solution, Addgene #104488) into the right NAc (AP + 1.3 mm, ML + 0.65 mm, DV -4.6 mm). A 400 μm optic fiber was then implanted at AP + 1.3 mm, ML + 0.65 mm, DV -4.55 mm and sealed in place using metabond. Mice were allowed to wake up under warmed conditions, and carprofen (50 mg/kg) was delivered subcutaneously each day for at least two days following the surgery. Some knockout mice did not survive post-surgery.

### Fiber photometry

Following at least 3 weeks for viral expression, mice underwent PMA as described above, but with 12 tone-shock pairings on training day and 9 tone deliveries on retrieval day to increase the number of trials over which average traces could be obtained from each mouse. Mice were handled and habituated to the fiber attachment for at least 3 days prior to recording. A TDT RZ10x processor in combination with the TDT Synapse software was used to record a 405 nm isosbestic channel and the 465 nm signal channel while mice performed the PMA task. TTL pulses marking the start and end of each tone were used to align behavior and photometry data. Data were analyzed as previously described by fitting a curve to the 405 nm isosbestic channel using the polyfit function in MATLAB and subtracting this from the 465 nm signal channel. Z scores were calculated based on the mean and SD of a baseline period of 10 seconds prior to event onset for shock responses, -20 to -15 seconds for platform entry responses, -5 to 0 s for platform exit responses, and -10 to -5 s for EZM open arm entries. Error bars in photometry traces are based on SEM of animal averages. For visualization, traces were smoothed by averaging values across each 0.5 s period.

### Reporting summary

Further information on research design is available in the Nature Portfolio Reporting Summary linked to this article.

## Supplementary information

Supplementary Information

Description of Additional Supplementary Files

Supplementary Data 1

Supplementary Data 2

Reporting Summary

Transparent Peer Review file

## Data Availability

The raw quantitative proteomics datasets generated in this study and the necessary metadata for replication and verification have been deposited in the MassIVE online database under accession code MSV000096918 (Gongwer-Etienne Proteomics Dataset Link). Minimal datasets and source data associated with this paper are available in Source Data files deposited to the Figshare repository (https://doi.org/10.6084/m9.figshare.33049709). Raw microscopy images are available upon request.
