# Supplementary material for: Microglia regulate nucleus accumbens synaptic development and circuit function underlying threat avoidance behaviors in mice
Source: Nat Commun. 2026 Sep 26;17:11156. doi: 10.1038/s41467-026-77487-z (PMC13710536; doi:10.1038/s41467-026-77487-z)
Supplement: Supplementary file 1 [file 41467_2026_77487_MOESM1_ESM.pdf]

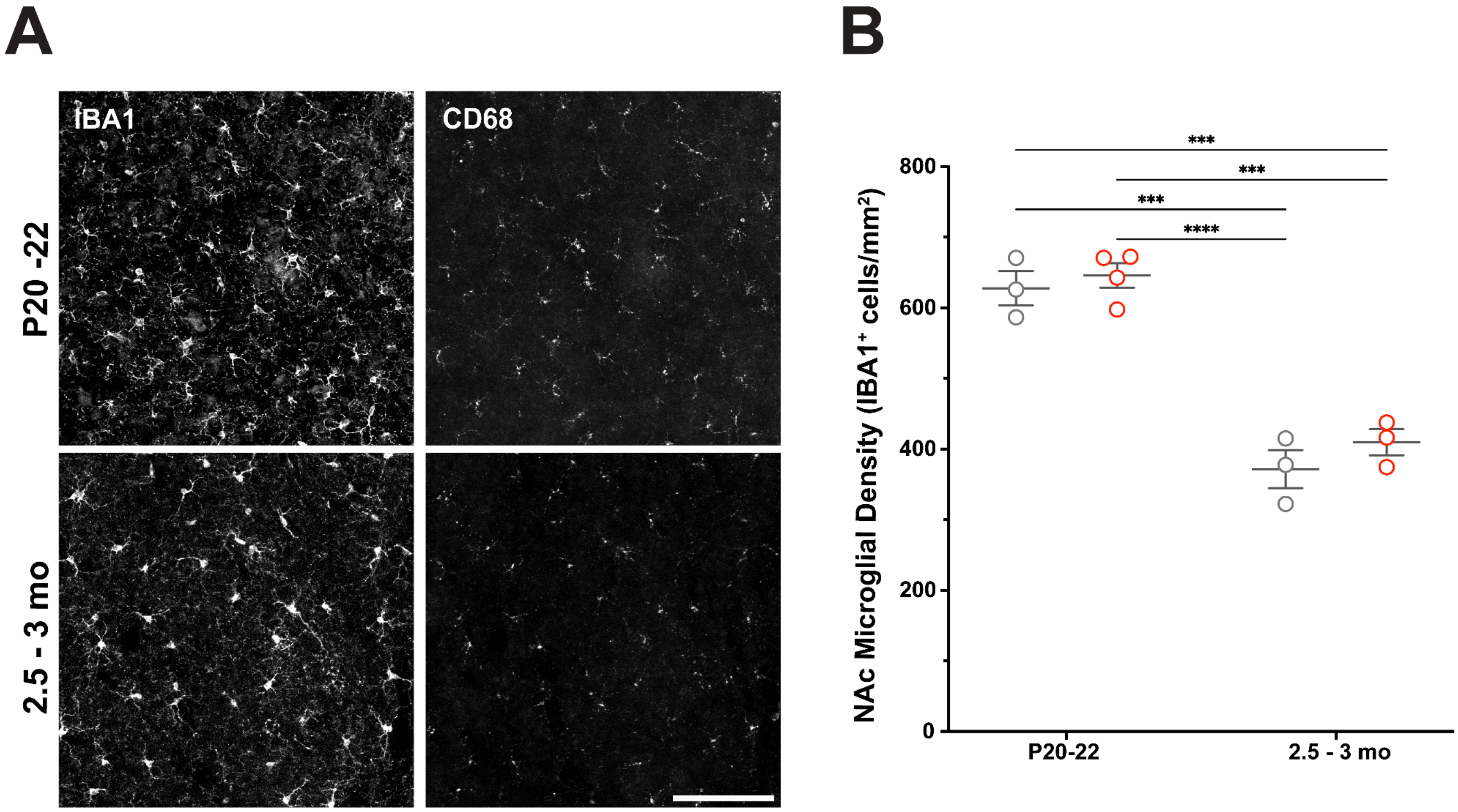

Figure S1: **Additional microglia density measurements in the Het mice. a** Representative images of NAc microglia in heterozygous (Het) mice at P20-22 and 2.5-3 months old. Microglia (IBA1) and their lysosomes (CD68) are both represented in gray. Scale bar: 100 µm. **b** NAc microglial density in WT (representative images and quantification) shown in *Figure 1A,B*) and Het mice. Two-way RM ANOVA with Šídák's multiple comparisons test. $F_{age}(1,9)=128.4$ p<0.0001, $F_{genotype}(1,9)=1.648$ *P*=0.2313, $F_{age\ x\ genotype}(1,9)=18.47$ *P*=0.2053. ****P*<0.001, *****P*<0.0001. Error bars represent mean ± SEM. Grey symbols, WT mice. Red symbols, Het mice. P20-22: WT *n*=3 mice; P20-22: KO *n*=4 mice; 2.5 - 3 mo: WT *n*=3 mice and 2.5 - 3 mo: KO *n*=3 mice. n represents biological replicates. Source data are provided as a Source Data file on the Figshare repository.

.

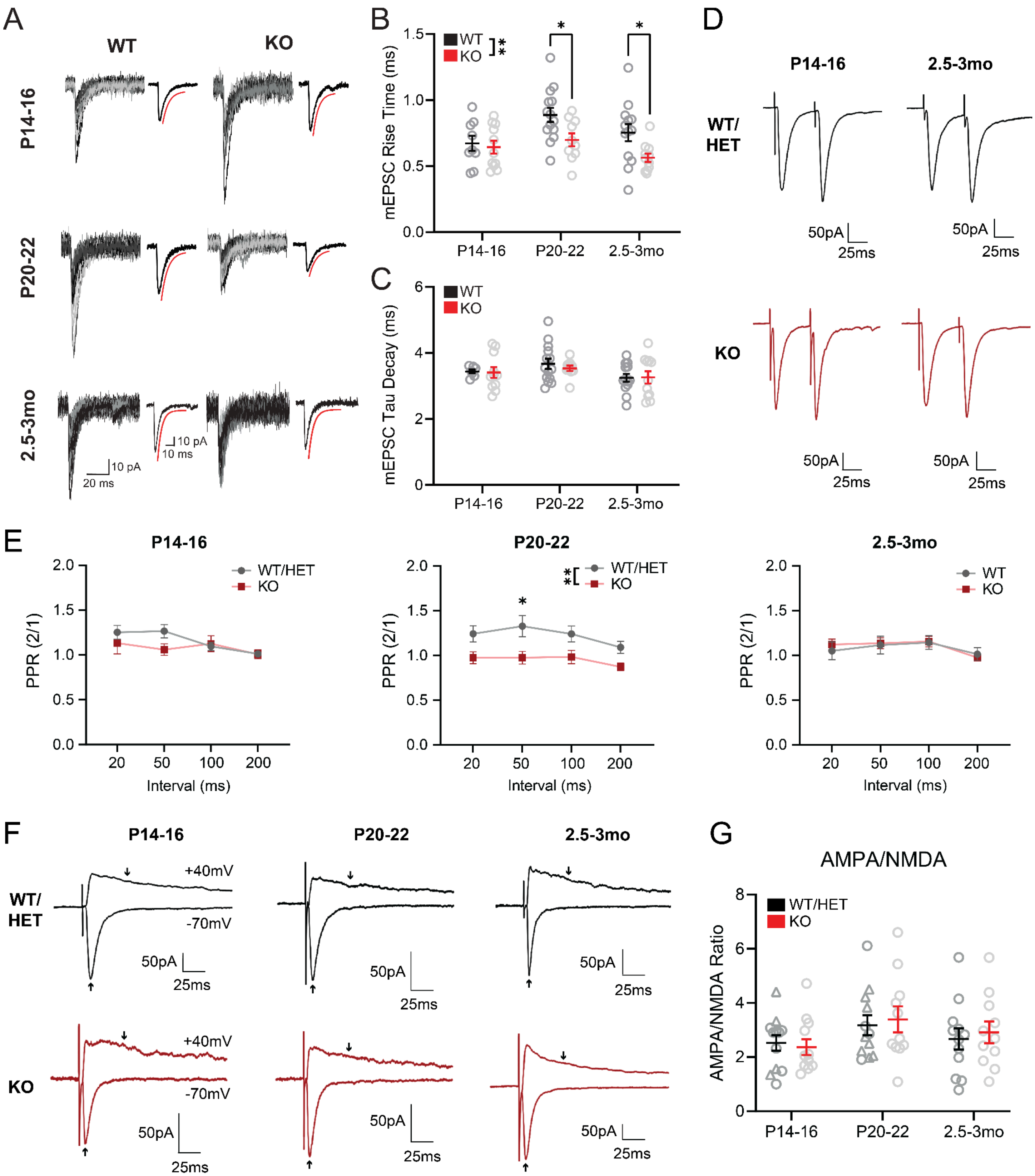

Figure S2: Additional electrophysiological measures of excitatory synaptic development in the presence or absence of microglia. a Overlay of 10 to 15 single mEPSC events in WT and KO mice at p14-16, p20-22 and 2.5-3mo. The average trace for each condition is represented on the right, and the fit exponential curve is shown in red. **b** mEPSC rise time in NAc of WT and KO mice at ages with a sufficient number of events for analysis of kinetics. $F_{age}(2,62)=4.268$,

P=0.0183; $F_{genotype}$(1,62)=9.472, P=0.0031; $F_{age\ x\ genotype}$(2,62)=1.390, P=0.2568. P14-16: WT n=9(6), KO n=11(5); P20-22: WT n=14(7), KO n=10(6); 2.5-3mo: WT n=13(7), KO n=11(6). **c** mEPSC Tau decay constant in NAc of WT and KO mice. F_{age}(2,60)=3.009, P=0.0569; $F_{genotype}$(1,60)=0.1783, P=0.6743; $F_{age\ x\ genotype}$(2,60)=0.1469, P=0.8637. Two-way ANOVA with Šídák's multiple comparisons test. P14-16: WT n=9(6), KO n=11 cells from 5 mice; P20-22: WT n=14 cells from 7 mice, KO n=10 cells from 6 mice; 2.5-3mo: WT n=13 cells from 7 mice, KO n=11 cells from 6 mice. **d** Representative traces of evoked paired-pulse ratio (PPR) recordings with a 50ms interstimulus interval in NAc knockout and control mice at P14-16 and 2.5-3mo. Cells were recorded while voltage clamping at -70mV in the presence of 100µM picrotoxin. **e** Paired-pulse ratio from interstimulus intervals ranging from 20 to 200ms. P14-16: $F_{Interval}$(3,69)=5.018, P=0.0033; $F_{Genotype}$(1,23)=0.8535, P=0.3652; $F_{Interval\ x\ Genotype}$(3,69)=2.294, P=0.0855. P20-22: $F_{Interval}$(3,60)=5.807, P=0.0240; $F_{Genotype}$(1,20)=5.962, P=0.0015; $F_{Interval\ x\ Genotype}$(3,60)=0.8502, P=0.4720. 2.5-3mo: $F_{Interval}$(3,69)=8.863, P<0.0001; $F_{Genotype}$(1,23)=0.02861, P=0.8672; $F_{Interval\ x\ Genotype}$(3,69)=1.001, P=0.3976. Two-way RM ANOVA with Šídák's multiple comparisons test. P14-16: WT/HET n=13(4), KO n=12(3); P20-22: WT/HET n=13 cells from 4 mice, KO n=12 cells from 4 mice; 2.5-3mo: WT n=12 cells from 4 mice, KO n=13 cells from 4 mice. **f** Representative traces of evoked AMPA/NMDA recordings in knockout and control mice. Cells were recorded while voltage clamping at -70mV or +40mV in the presence of 100µM picrotoxin. Arrows indicate timepoints used for current estimation: the NMDA current was taken as the current value from the +40mV stimulation 50 ms after stimulus onset, and the AMPA current was taken as the peak amplitude of the evoked current when held at -70mV. **g** AMPA/NMDA ratio in NAc of KO and control mice at P14-16, P20-22, and 2.5-3mo. F_{Age}(2,64)=2.562, P=0.0850; $F_{Genotype}$(1,64)=0.1112, P=0.7399; $F_{Age\ x\ Genotype}$(2,64)=0.1802, P=0.8355. Two-way ANOVA. P14-16: WT/HET n=12 cells from 4 mice, KO n=12 cells from 3 mice; P20-22: WT/HET n=12 cells from 5 mice, KO n=11 cells from 4 mice; 2.5-3mo: WT n=12 cells from 4 mice, KO n=11 cells from 4 mice. Error bars represent mean ± SEM. *P<0.05. **P<0.01. Data points shown as triangles indicate heterozygous (Csf1r$^{\Delta FIRE/WT}$) mice. Black symbols, WT mice. Red symbols, KO mice. n represents biological replicates. Source data are provided as a Source Data file on the Figshare repository.

.

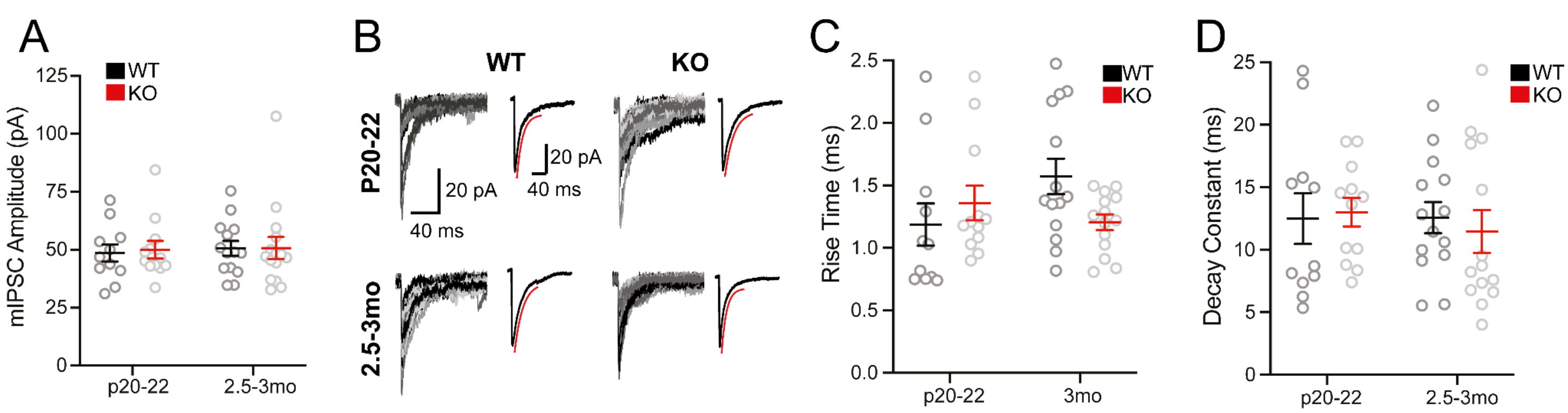

Figure S3: Additional electrophysiological measures of inhibitory synaptic development in the presence or absence of microglia. a mIPSC amplitude in NAc of WT and KO mice at P20-22 and 2.5-3mo. F_{age}(1,48)=0.1186, P=0.7321; $F_{genotype}$(1,48)=0.03294, P=0.8568; $F_{age\ x\ genotype}$(1,48)=0.02360, P=0.8785. P20-22: WT n=11 cells from 5 mice, KO n=13 cells from 5 mice; 2.5-3mo: WT n=14 cells from 6 mice, KO n=15 cells from 6 mice. **b** Overlay of 10 to 15 single mIPSC events in both WT and KO mice at P20-22 and 2.5-3mo. The average trace for each condition is represented on the right, and the fit exponential curve is shown in red. (**c-d**) mIPSC rise time **c** and Tau decay constant **d** in NAc of WT and KO mice at P20-22 and 2.5-3mo. Rise time: F_{age}(1,47)=0.7925, P=0.3779; $F_{genotype}$(1,47)=0.5576, P=0.4589; $F_{age\ x\ genotype}$(1,47)=4.296, P=0.0437; Tau Decay: F_{age}(1,47)=0.2174, P=0.6432; $F_{genotype}$(1,47)=0.03584, P=0.8507; $F_{age\ x\ genotype}$(1,47)=0.2694, P=0.6062. Two-way ANOVA with Šídák's multiple comparisons test. P20-22: WT n=11 cells from 5 mice, KO n=12 cells from 5 mice; 2.5-3mo: WT n=14 cells from 6 mice, KO n=15 cells from 6 mice. Error bars represent mean ± SEM. Black symbols, WT mice. Red symbols, KO mice. n represents biological replicates. Source data are provided as a Source Data file on the Figshare repository.

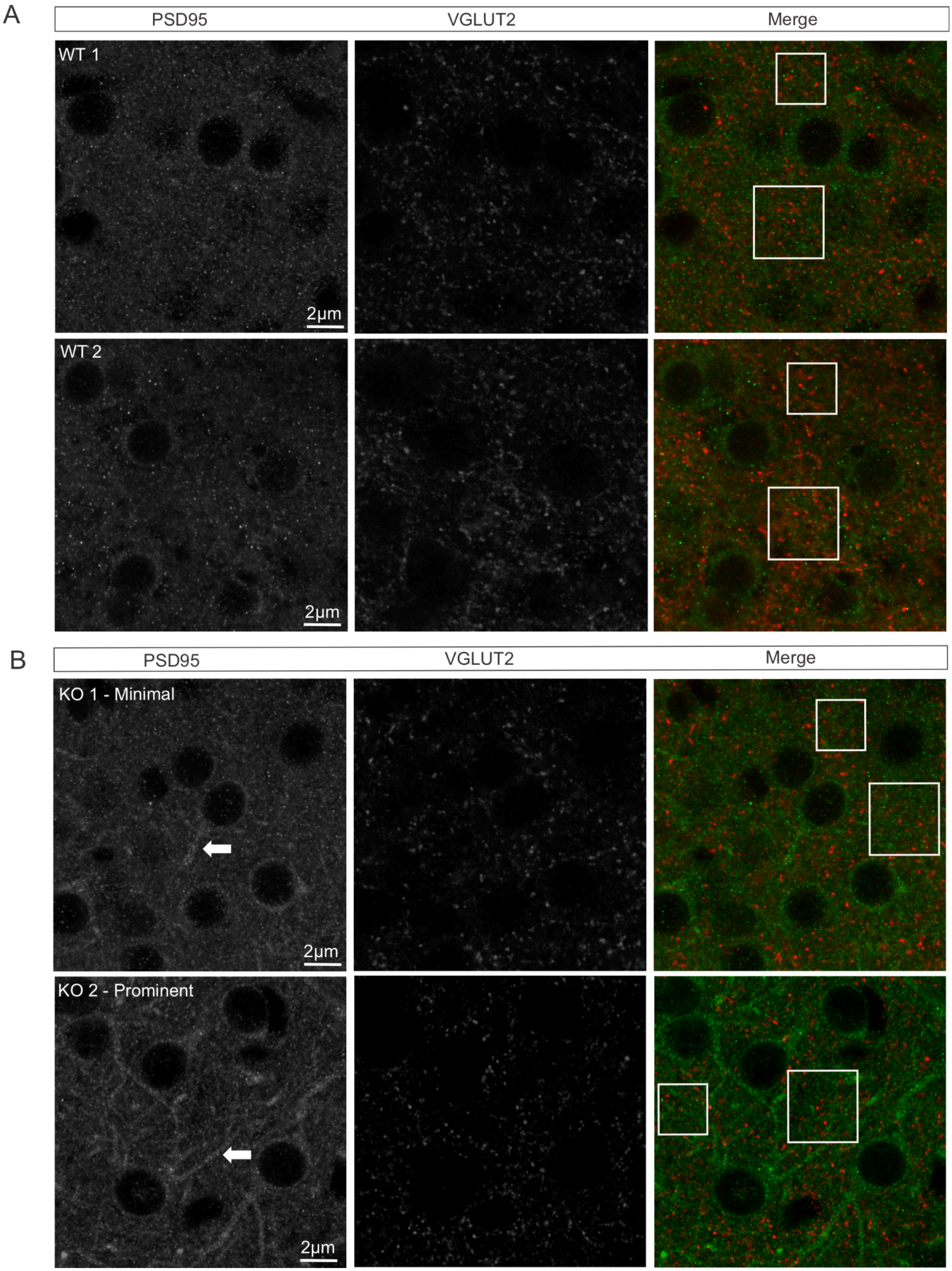
A
PSD95
VGLUT2
Merge
WT 1
2μm
WT 2
2μm
B
PSD95
VGLUT2
Merge
KO 1 - Minimal
2μm
KO 2 - Prominent
2μm

Figure S4. Additional examples of excitatory synapse staining in mice with and without microglia. a Representative images of PSD95 and VGLUT2 immunostaining from two WT animals. *White boxes* indicate non-adjacent ROIs selected for analyses; one 45x45 µm and one 80x80 µm ROI were analyzed per image, with location selected to avoid MSN somas (*dark circles*). **b** Representative images of PSD95 and VGLUT2 immunostaining from two KO animals. *White arrows* indicate examples of diffuse “dendritic-like” PSD95 staining that were minimal in some KO animals (*top panels*) and more prominent in other KO animals (*bottom panels*).

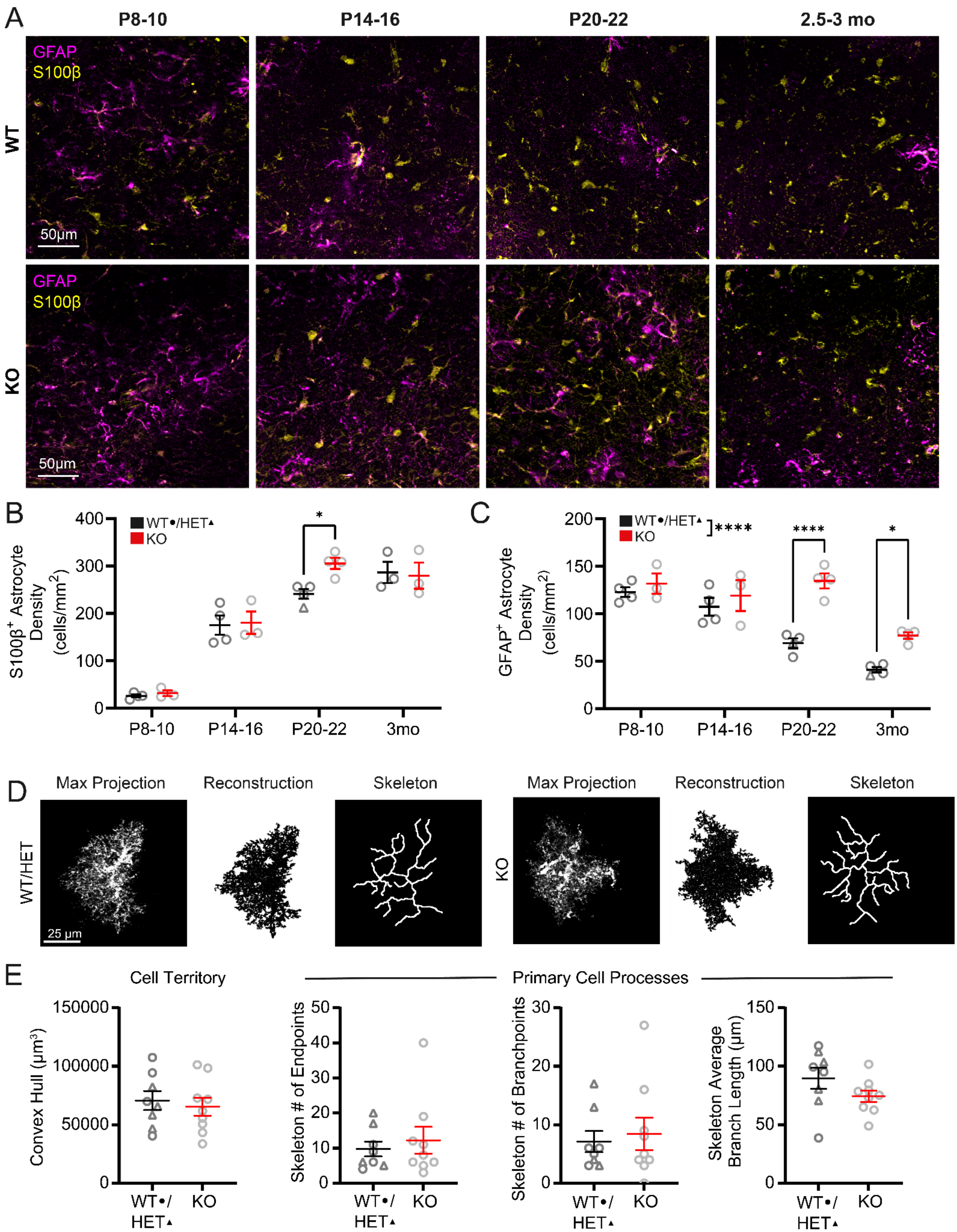

A
P8-10
P14-16
P20-22
2.5-3 mo
WT
KO
GFAP
S100β
50μm
B
S100β+ Astrocyte Density (cells/mm2)
WT•/HET▲
KO
P8-10
P14-16
P20-22
3mo
C
GFAP+ Astrocyte Density (cells/mm2)
WT•/HET▲
KO
D
Max Projection
Reconstruction
Skeleton
WT/HET
KO
25 μm
E
Cell Territory
Primary Cell Processes
Convex Hull (μm3)
Skeleton # of Endpoints
Skeleton # of Branchpoints
Skeleton Average Branch Length (μm)
WT•/HET▲
KO

Figure S5: Absence of microglia alters normal developmental trajectories of astrocyte markers in NAc. a Representative images of S100β$^+$ and GFAP$^+$ astrocytes in NAc of WT and KO mice at p8-10, p14-16, p20-22 and 2.5-3mo. Zoom panels represent the area with a dashed outline. **b** S100β$^+$ astrocyte density in NAc of WT and KO mice across postnatal development. $F_{age}(3,20)=99.74$, $P=3.3x10^{-12}$; $F_{genotype}(1,20)=2.092$, $P=0.1635$; $F_{age\ x\ genotype}(3,20)=2.004$, $P=0.1458$, Two-way ANOVA with Šídák's multiple comparisons test. P8-10: WT *n*=3, KO *n*=3; P14-16: WT *n*=4, KO *n*=3; P20-22: WT *n*=3, KO *n*=4; 2.5-3mo: WT *n*=3 mice, KO *n*=3 mice. **c** GFAP$^+$ astrocyte density in KO and control mice across postnatal development. $F_{age}(3,22)=29.29$, $P=7.3x10^{-8}$; $F_{genotype}(1,22)=30.90$, $P=1.4x10^{-5}$; $F_{age\ x\ genotype}(3,22)=5.798$, $P=0.0045$; Two-way ANOVA with Šídák's multiple comparisons test. P8-10: WT *n*=4 mice, KO *n*=3 mice; P14-16: WT *n*=4 mice, KO *n*=3 mice; P20-22: WT *n*=3 mice, KO *n*=4 mice; 2.5-3mo: WT/HET *n*=4 mice, KO *n*=4 mice. **d** Maximum intensity projections of high magnification confocal stacks of individual NAc astrocytes in WT/HET and KO mice. Panels *at right* illustrate volumetric reconstructions and analysis of cell territory and branching structure carried out using 3D Morph. **e** Comparison of NAc astrocyte territory (convex hull), number of primary process endpoints (from skeleton; does not capture fine leaflets), primary process branchpoints, and average primary process branch length across genotypes. Two-tailed unpaired t test (territory: *P*=0.6506, # endpoints: *P*=0.5920, # branchpoints: *P*=0.7039, branch length: *P*=0.1492). WT/HET: *n*=8 cells from 3 mice; KO: *n*=8 cells from 3 mice. Error bars represent mean ± SEM. *$P<0.05$, ****$P<0.0001$. Data points shown as triangles indicate heterozygous ($Csf1r^{\Delta FIRE/WT}$) mice. Black symbols, WT mice. Red symbols, KO mice. n represents biological replicates. Source data are provided as a Source Data file on the Figshare repository.

.

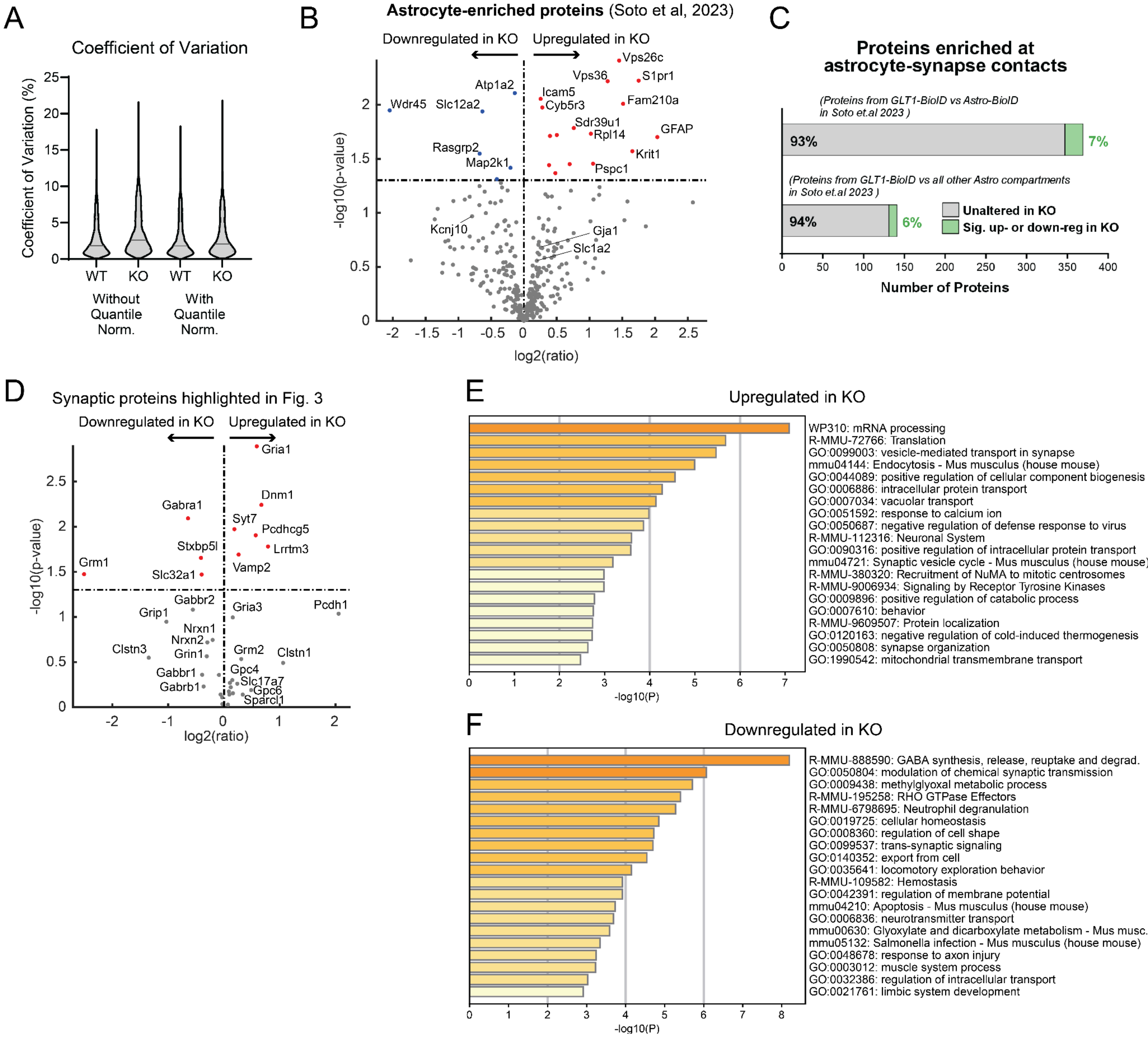

Figure S6. **Additional analysis of proteomic dataset. a** Coefficient of variation for individual proteins across biological replicates within each condition, calculated from Log2-transformed values with or without quantile normalization. **b** Volcano plot of protein expression in WT vs KO mice in 342 proteins shown to be enriched/unique for astrocytes compared to neurons in Soto et. al. 2023. **c** Percentage of proteins enriched at astrocyte-synapse contacts that are either unaltered (gray) or significantly up- or downregulated (green) in WT vs KO mice. **d** Volcano plot of protein expression in WT vs KO mice for the synaptic proteins highlighted in Figure 3. **e-f** Complete list of top 20 pathways upregulated **e** and downregulated **f** in KO mice upon Metascape analysis of differentially expressed proteins from proteomic data. Grey symbols, WT mice. Red symbols, KO mice. Source data are available in Supplementary Data 1 and Supplementary Data 2 associated with this manuscript.

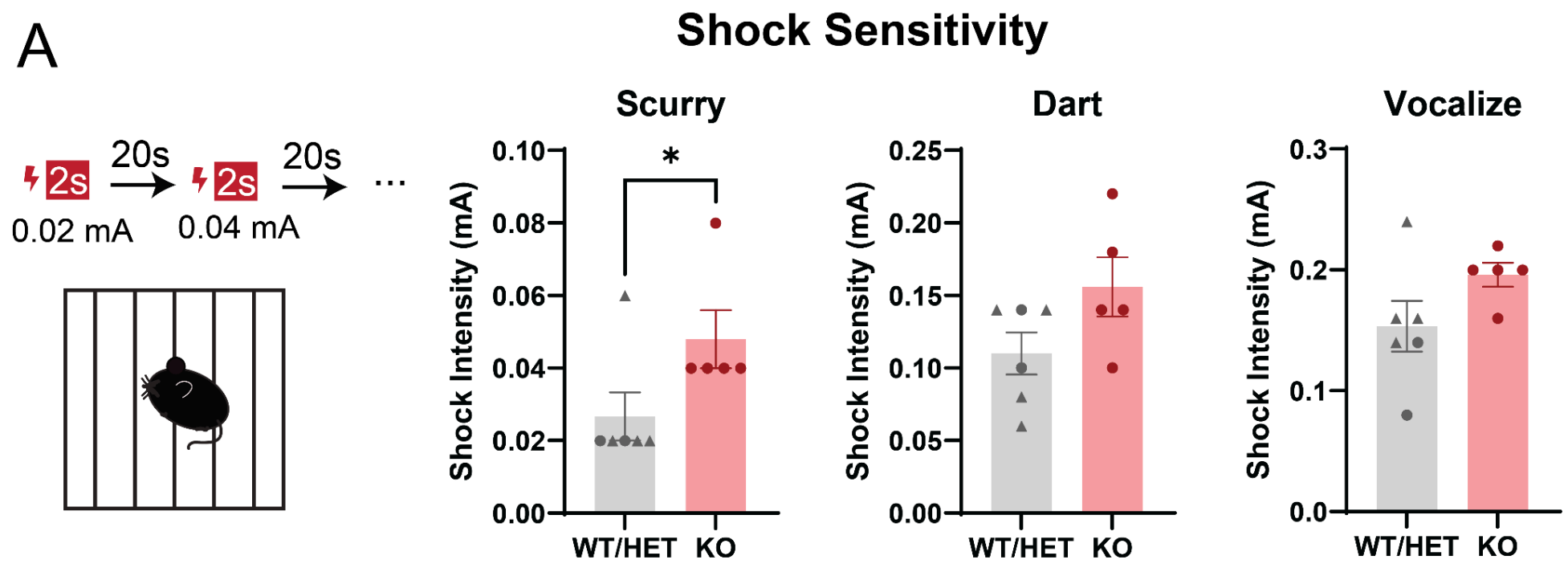

Figure S7. **Shock sensitivity of mice with and without microglia. a** Minimum shock intensity required to elicit scurry, dart, and vocalization responses in knockout and control mice. Mann-Whitney test (Scurry: *P*=0.0130, Dart: *P*=0.1342, Vocalize: *P*=0.1061), WT/HET: *n*=6 mice, KO: *n*=5 mice. **P*<0.05. Error bars represent mean ± SEM. Data points shown as triangles indicate heterozygous (Csf1r$^{\Delta FIRE/WT}$) mice. Grey symbols, WT mice. Red symbols, KO mice. n represents biological replicates. Source data are provided as a Source Data file on the Figshare repository.

.

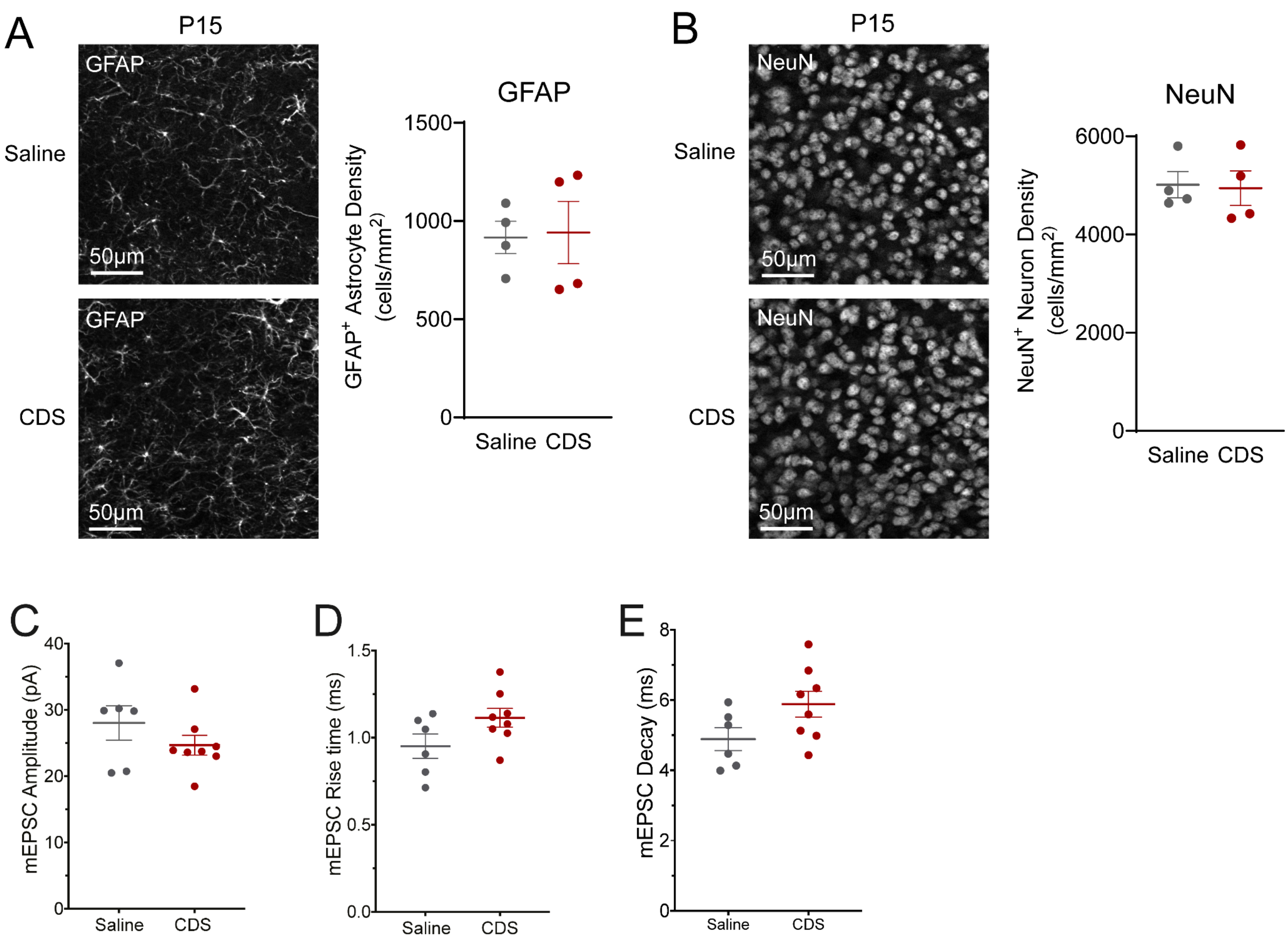

Figure S8. **Status of astrocytes and neurons in CDS treated animals. a** NAc $GFAP^{+}$ astrocyte density in CDS-injected mice at P15. Two-tailed unpaired t-test (*P*=0.8933), CDS: *n*=4 mice, Saline: *n*=4 mice. (B) NAc $NeuN^{+}$ neuron density in CDS-injected mice at P15. Two-tailed unpaired t-test (*P*=0.8766), CDS: *n*=4 mice, Saline: *n*=4 mice. **c-e** Average mEPSC

amplitude, rise time, and decay calculated from mEPSCs recorded from NAc MSNs of saline and CDS treated mice at p20-22. Two-tailed unpaired t-test (amplitude: P=0.2560, rise time: P=0.0845, decay: P=0.0772), Saline: n=6 cells from 3 mice, CDS: n=8 cells from 3 mice. Grey symbols, WT mice. Red symbols, KO mice. n represents biological replicates. Source data are provided as a Source Data file on the Figshare repository.

.
