# Supplementary material for: Microglia regulate nucleus accumbens synaptic development and circuit function underlying threat avoidance behaviors in mice
Source: Nat Commun. 2026 Sep 26;17:11156. doi: 10.1038/s41467-026-77487-z (PMC13710536; doi:10.1038/s41467-026-77487-z)
Supplement: Supplementary file 2 [file 41467_2026_77487_MOESM2_ESM.pdf]

Description of Additional Supplementary Files

File Name: Supplementary Data 1
Description: Log2 transformed protein intensity data for NAc tissue proteomics in P22 KO and CTR mice.

File Name: Supplementary Data 2
Description: Complete Metascape pathway analyses for proteins that were significantly upregulated and significantly downregulated in KO compared to CTR mice.
