# Supplementary material for: Microglia regulate nucleus accumbens synaptic development and circuit function underlying threat avoidance behaviors in mice
Source: Nat Commun. 2026 Sep 26;17:11156. doi: 10.1038/s41467-026-77487-z (PMC13710536; doi:10.1038/s41467-026-77487-z)
Supplement: Supplementary file 3 [file 41467_2026_77487_MOESM5_ESM.pdf]

nature portfolio

Corresponding author(s): Lindsay De Biase, Laura DeNardo

Last updated by author(s): Jul 17, 2026

Reporting Summary

Nature Portfolio wishes to improve the reproducibility of the work that we publish. This form provides structure for consistency and transparency in reporting. For further information on Nature Portfolio policies, see our Editorial Policies and the Editorial Policy Checklist.

Statistics

For all statistical analyses, confirm that the following items are present in the figure legend, table legend, main text, or Methods section.

n/a	Confirmed	
☐	☒	The exact sample size (*n*) for each experimental group/condition, given as a discrete number and unit of measurement
☐	☒	A statement on whether measurements were taken from distinct samples or whether the same sample was measured repeatedly
☐	☒	The statistical test(s) used AND whether they are one- or two-sided<br>*Only common tests should be described solely by name; describe more complex techniques in the Methods section.*
☒	☐	A description of all covariates tested
☐	☒	A description of any assumptions or corrections, such as tests of normality and adjustment for multiple comparisons
☐	☒	A full description of the statistical parameters including central tendency (e.g. means) or other basic estimates (e.g. regression coefficient) AND variation (e.g. standard deviation) or associated estimates of uncertainty (e.g. confidence intervals)
☐	☒	For null hypothesis testing, the test statistic (e.g. *F*, *t*, *r*) with confidence intervals, effect sizes, degrees of freedom and *P* value noted<br>*Give P values as exact values whenever suitable.*
☒	☐	For Bayesian analysis, information on the choice of priors and Markov chain Monte Carlo settings
☒	☐	For hierarchical and complex designs, identification of the appropriate level for tests and full reporting of outcomes
☒	☐	Estimates of effect sizes (e.g. Cohen's *d*, Pearson's *r*), indicating how they were calculated

Our web collection on statistics for biologists contains articles on many of the points above.

Software and code

Policy information about availability of computer code

Data collection	pClamp v10 + v11, BehaviorDEPOT v1.3b, TDT Synapse 95, Leica LASX, Zeiss Zen Blue (v2.6)
Data analysis	We used ImageJ v1.53t for image analysis. We used miniAnalysis v6.0.8 (Synaptosoft) and Python v3.7 for electrophysiology analysis. For proteomics, we used MaxQuant for peptide identification and MATLAB (version 2023b) for plotting and comparisons. We used Metascape and SynGo for pathway analysis. We used Fiji (opensource) and Imaris v10.1 for analysis of microscope images. We used 3D Morph (MATLAB based open-access image analysis tool) for reconstruction and morphological analysis of astrocytes. We used DeepLabCut v2.0 (opensource), a markerless pose estimation software, and a custom behavioral classification software BehaviorDEPOT v1.3b (opensource) with custom MATLAB (version 2023b) Scripts to generate behavioral measures in PMA. We used Viewer III (Biobserve) for analysis of open field and elevated zero maze. Custom MATLAB Scripts based on samples from TDT (https://www.tdt.com/docs/fiber-photometry-user-guide/data-analysis/) were used to analyze fiber photometry data. We used Prism10 (v10.3.1) (GraphPad), Origin 2019 (v9.60), and MATLAB (version 2023b) for statistical analysis.

For manuscripts utilizing custom algorithms or software that are central to the research but not yet described in published literature, software must be made available to editors and reviewers. We strongly encourage code deposition in a community repository (e.g. GitHub). See the Nature Portfolio guidelines for submitting code & software for further information.

Data

Policy information about availability of data

All manuscripts must include a data availability statement. This statement should provide the following information, where applicable:

- Accession codes, unique identifiers, or web links for publicly available datasets
- A description of any restrictions on data availability
- For clinical datasets or third party data, please ensure that the statement adheres to our policy

The raw quantitative proteomics datasets generated in this study and the necessary metadata for replication and verification have been deposited in the MassIVE online database under accession code MSV000096918 (FTP download link: ftp://MSV000096918@massive-ftp.ucsd.edu). Minimal datasets and source data are provided with this paper in Source Data files. Raw microscopy images are available upon request.

Research involving human participants, their data, or biological material

Policy information about studies with human participants or human data. See also policy information about sex, gender (identity/presentation), and sexual orientation and race, ethnicity and racism.

Reporting on sex and gender	N/A
Reporting on race, ethnicity, or other socially relevant groupings	N/A
Population characteristics	N/A
Recruitment	N/A
Ethics oversight	N/A

Note that full information on the approval of the study protocol must also be provided in the manuscript.

Field-specific reporting

Please select the one below that is the best fit for your research. If you are not sure, read the appropriate sections before making your selection.

- [x] Life sciences
- [ ] Behavioural & social sciences
- [ ] Ecological, evolutionary & environmental sciences

For a reference copy of the document with all sections, see nature.com/documents/nr-reporting-summary-flat.pdf

Life sciences study design

All studies must disclose on these points even when the disclosure is negative.

Sample size	Sample size was estimated based on existing published literature (Parkhurst et al 2013, Soto et al 2023, Moscarello et al., 2013, Gongwer et al., 2023), but not with predetermined statistical methods.
Data exclusions	For slice electrophysiology data, cells were excluded if series resistance changed by more than 20% across a recording session. Animals were excluded from the study due to mistargeting of the fiber implant in fiber photometry experiments, which resulted in a lack of fluorescent signal.
Replication	Each experiment represents multiple successfully replicated independent cohorts.
Randomization	Heterozygous breeders were crossed to produce a mix of wild-type and homozygous knockout littermates. Whenever possible, WT and KO littermates were used for the same experiment.
Blinding	Data collection and behavioral video and image analysis were performed by experimenters blind to the experimental groups. Behavioral data was analyzed blind to experimental conditions using automated analysis pipelines. For manual image analyses, researchers were blind to experimental manipulation

Reporting for specific materials, systems and methods

We require information from authors about some types of materials, experimental systems and methods used in many studies. Here, indicate whether each material, system or method listed is relevant to your study. If you are not sure if a list item applies to your research, read the appropriate section before selecting a response.

Materials & experimental systems

n/a	Involved in the study
☐	☒ Antibodies
☒	☐ Eukaryotic cell lines
☒	☐ Palaeontology and archaeology
☐	☒ Animals and other organisms
☒	☐ Clinical data
☒	☐ Dual use research of concern
☒	☐ Plants

Methods

n/a	Involved in the study
☒	☐ ChIP-seq
☒	☐ Flow cytometry
☒	☐ MRI-based neuroimaging

Antibodies

Antibodies used

Guinea Pig anti-PSD95 (Synaptic Systems #124308, 1:800), Mouse anti-VGLUT2 (DSHB N29/29, 1:150), Rat anti-Gephyrin (Synaptic Systems #147208, 1:500), Mouse anti-GAD6 (DSHB GAD-6, 1:100), Rat anti-CD68 (Serotec clone FA-11 #MCA1957, 1:200), rabbit anti-Iba1 (Wako #019-19741, 1:500), Rabbit Anti-GFAP (Dako, Agilent #Z0334, 1:500), Mouse Anti-S100β (Sigma-Aldrich #S2532, 1:500), Chicken Anti-TH (Aves #TYH, 1:500), Rabbit Anti-c-Fos (Synaptic Systems #226008, 1:1000), Chicken Anti-GFP (Aves #GFP1010, 1:2000), Rabbit Anti-V5 (Bethyl #A190-120A, 1:1000), Alexa 488 Donkey Anti-Rabbit (Jackson ImmunoResearch #711-545-152) or Anti-Chicken (#703-545-155), Alexa 594 Donkey Anti-Chicken (#703-585-155), Alexa 647 Donkey Anti-Mouse (#715-605-151), all 1:1000)

Validation

IBA1: validated through immunohistochemical staining of microglia-depleted mice (Csf1rΔFIRE/ΔFIRE)
TH Tyrosine hydroxilase: This antibody has been used for IHC in 50+ peer-reviewed publications.
CD68: This antibody has been used for IHC in at least 100+ peer-reviewed publications.
GFAP: This antibody has been used for IHC in at least 500+ peer-reviewed publications
S100β: This antibody has been used for IHC in at least 100+ peer-reviewed publications
Fos: This antibody has been used for IHC in at least 50+ peer-reviewed publications.
PSD95: This antibody has been used for IHC in at least 100+ peer-reviewed publications
VGLUT2: This antibody has been used for IHC in at least 100+ peer-reviewed publications
Gephyrin: This antibody has been used for IHC in at least 50+ peer-reviewed publications.
GAD6: This antibody has been used for IHC in at least 50+ peer-reviewed publications.

Animals and other research organisms

Policy information about studies involving animals; ARRIVE guidelines recommended for reporting animal research, and Sex and Gender in Research

Laboratory animals

Csf1rΔFIRE/WT mice were obtained from the Pridans lab at the University of Edinburgh and crossed with wild-type CBA mice (Jackson laboratories #000656). Resulting Csf1rΔFIRE/WT mice were crossed to produce Csf1rΔFIRE/ΔFIRE (KO), Csf1rΔFIRE/WT (HET), and Csf1rWT/WT (WT) littermates. KO, WT and some HET mice were used for experiments, aged postnatal day 8-120 as stated in the methods. Wildtype mice (C57Bl6) used for clodronate disodium salt experiments were obtained from Jackson Laboratories (Strain 000664).

Wild animals

None used.

Reporting on sex

All experiments include mice of both sexes in roughly even numbers. Studies were not powered to treat sex as a variable. No obvious sex differences were observed.

Field-collected samples

None used.

Ethics oversight

All procedures followed animal care guidelines approved by the University of California, Los Angeles Chancellor’s Animal Research Committee. (UCLA Animal Research Committee (ARC) and UCLA institutional animal care and use committee (IACUC)).

Note that full information on the approval of the study protocol must also be provided in the manuscript.

Plants

Seed stocks	N/A
Novel plant genotypes	N/A
Authentication	N/A
