# Supplementary material for: Microglia regulate nucleus accumbens synaptic development and circuit function underlying threat avoidance behaviors in mice
Source: Nat Commun. 2026 Sep 26;17:11156. doi: 10.1038/s41467-026-77487-z (PMC13710536; doi:10.1038/s41467-026-77487-z)
Supplement: Supplementary file 4 [file 41467_2026_77487_MOESM6_ESM.pdf]

nature portfolio

Peer Review File

Microglia regulate nucleus accumbens synaptic development and circuit function underlying threat avoidance behaviors

Corresponding Author: Dr Lindsay De Biase

This file contains all reviewer reports in order by version, followed by all author rebuttals in order by version.

Version 0:

Reviewer comments:

Reviewer #1

(Remarks to the Author)
The authors examined the impact of microglial deletion in the FIRE mice on NAc synapse dynamics and function, reporting decreases in the frequence of mEPSCs at P20-22 along with proteomic changes consistent with a change in synapse development. FIRE mice also show changes in threat avoidance behavior and anxiety, as well as altered NAc activity during the performance of these tasks. These data are interesting and add to the now large literature indicating microglia play important roles in normal brain development, especially during discrete developmental windows that are brain region and time point-dependent. There are some leaps in conclusions by the authors that are not necessarily supported by the data, along with some confusing aspects of the paper that should be addressed in revision.

Major:
1. The authors conclude that the lack of microglia leads to a reduction in excitatory synapse number based on electrophysiology, which is convincing, but should be supported with histological assessments of synapse number.
2. The authors further conclude that the observed effects on synapse function are not due to changes in astrocytes based on proteomics data in which secreted proteins are not significantly altered. This is a considerable leap, given that astrocytes perform many functions to support synapse function aside from secreted proteins, as the authors themselves point out, including structural and morphological impacts e.g. via adhesion proteins that may not be changed in total number, but in location/contact with synapses. Morphological assessments of astrocytes should be completed to investigate these possibilities.
3. “Thus, we identified key signaling proteins that may play previously unappreciated roles in microglia-mediated synapse formation in the NAc and that merit dedicated follow-up studies”. Given that this conclusion is entirely speculative, the authors should either include functional follow up studies, or omit this discussion.

Minor/clarification:
1. Lines 126-130 – Shouldn’t this read “suggesting that microglia do NOT significantly impact relative abundance of these receptors at NAc glutamatergic…”?
2. Were recordings done in core vs shell of NAc, or no distinction? Same for proteomic dissections?
3. For Fig. 2C-D. Please clarify the different columns. Are these technical replicates?

Reviewer #2

(Remarks to the Author)
This study by Gongwer, Etienne et al. investigates the consequences of a lack of microglia on the nucleus accumbens (NAc). Using microglia-lacking FIRE mice, the authors first investigate synapse function and find a decrease in mEPSC frequency in NAc neurons, accompanied by an increase in presynaptic release probability, which are most pronounced in the third postnatal week (P20-22) and normalize in adulthood. The authors then perform proteomics of microdissected NAc tissue from P20-22 FIRE mice and find changes in levels of synaptic proteins and pathways related to synaptic processes. Behavioral testing of adult FIRE mice shows defects in threat avoidance behavior and heightened anxiety. In vivo calcium imaging of NAc neurons in FIRE mice reveals changes in NAc neuron activity that can be linked to these behavioral changes. Together, the results show that microglia play a role in establishing NAc synaptic connectivity important for threat avoidance behavior. This is a timely study that is clearly written and presented. However, the results as they are currently

presented are too preliminary and do not support strong conclusions. It is difficult to link the data present in Figures 1 and 2 with the data presented Figures 3-4. Without further validation, the results from Figure 2 remain speculative. With additional experimental evidence this would be a much stronger study.

Major

1. Throughout the paper, the authors claim that excitatory synaptogenesis is impaired in FIRE mice (e.g. Page 4 '…the prominent reduction in excitatory synapse number…'). This claim is based on their observation that mEPSC frequency is decreased, but release probability is increased in P20-22 FIRE mice. However, there is no analysis of synapse density provided in the study. mEPSC frequency can be affected by other factors than synapse number. The authors should assess excitatory synapse density in the NAc. At minimum, this can be done using immunohistochemistry for pre- and postsynaptic markers in the NAc, but to be able to link synapse density more directly to their electrophysiological observations, the authors could for instance fill recorded neurons and reconstruct dendrites and dendritic spines.
2. The mIPSC measurements are based on a very low number of neurons and animals and do not allow for clear conclusions. This is important to address because the proteomics analysis in Figure 2E shows that the top downregulated term is related to inhibitory synapses. These recordings need to be repeated, and the number of mice/neurons needs to be increased. These observations then need to be linked to synapse density measurements as mentioned above.
3. There is a significantly faster mIPSC rise time in adult FIRE mice and increased mIPSC decay time in P20-22 FIRE mice (Figure S2C). The authors speculate that this could be due to a change in GABAAR subunit composition. This should be validated.
4. Proteomics data (Figure 2, S4) lacks a detailed description and quality controls: it is not clear from the methods how the samples were analyzed (e.g. which mass spectrometer was used), how many proteins were identified, how many proteins were significant and what the variability between samples was. Figure 2C and D suggest a surprising variability between samples within conditions; a PCA plot of samples and plot of sample coefficient of variation between conditions can be provided. The accompanying supplemental table lacks information on p-value and fold-change in protein levels, it is unclear what the values in columns D-M represent.
5. The interpretation of the authors of their proteomic data is unclear and remains speculative. They observe significant changes in some synaptic proteins (e.g. glutamate receptor subunit Gria1 and metabotropic glutamate receptor Grm1) yet conclude that 'protein components necessary to form functional synapses are still adequately produced in KO mice'. This is not supported by the data, most downregulated GO terms relate to the synapse. The authors seem to contradict this conclusion in the next paragraph where they mention that SynGO analysis 'further supported the observation that microglial absence impacts numerous synapse relevant proteins'. The relevant synaptic proteins should be indicated in the volcano plot to allow the reader to judge the biological significance of these changes; this is difficult to judge from the normalized plots in Figure 2C and D. It is also not clear how the highlighted proteins in Figure 2C and D were selected, is this an exhaustive list? For example, the GO analysis in Figure 2E shows multiple synaptic terms in the downregulated list related to synaptic structure and function: what proteins are in these terms and where do they fall on the volcano plot?
6. It remains difficult to interpret how the observations in Figure 1-2 are linked to the results in Figure 3-4. It appears that the authors suggest that defects in synaptic development in the absence of microglia result in lasting defects in behavior later in life. However, microglia are also absent in adult FIRE mice, and the changes in NAc neuronal activity observed at this stage may result from an effect of microglia on neuronal activity, independent of synaptic changes during postnatal development.

Minor

1. The statements in the Discussion 'suggestion that lack of microglial-derived cues may underlie observed deficits in synapse formation', and 'we identified key signaling proteins that may play previously unappreciated roles in microglia-mediated synapse formation in the NAc and that merit dedicated follow-up studies' are not supported by the evidence provided.
2. The number of animals/cells analyzed is not always clear from the figure legends (e.g. PPR experiments, Figure 1G). Please indicate these.
3. Figure 1G: please explain rationale for including heterozygotes in control group: were these validated to not show changes in microglia number at the timepoints investigated?
4. Figure S2: what was the reason not to include mIPSC data from P8-10 and P14-16 mice?
5. Figure 2G - pipeline to find interesting candidates of how microglia could contribute to synapse formation, please explain this pipeline in more detail. The +++ and ++ in the table are subjective. Please also select only NAc RNAseq data and not whole brain data in your pipeline. It is not clear why the expression must be higher compared to other brain regions, is this because of NAc region specificity?
6. Figure 2: some validation of the proteomics data would enhance the study (e.g. the changes in Gria1 or Grm1 levels).
7. Figure S4: please provide an explanation of how the astrocyte-specific proteins were identified, we could not find this in the Methods. The text on page 4 is misleading: 'the majority of astrocyte enriched proteins identified by astrocyte-specific proteomics were not significantly altered across genotype' – if understood correctly the authors cross reference their tissue-level NAc proteomics dataset with another study to identify putative astrocytic proteins in their dataset. This should be clarified.
8. Figure S4A: S1PR1 is upregulated in KO. This protein has been shown to regulate the secretion of astrocyte-derived cues (Singh et al. 2022 Glia). Please comment on this observation.
9. Figure 3E: the data seems to be ordinal (having fixed values). This should not be an unpaired t-test to compare the means, but a Mann Whitney U test.

Reviewer #3

(Remarks to the Author)
In this manuscript, Gongwer & Etienne et al take advantage of a genetically modified mouse that lacks microglia to elucidate the role of microglia in regulating the synaptic landscape of the developing NAc and adult behavioral repertoires. The authors showed that absence of microglia blunts NAc excitatory synaptogenesis, which was accompanied by many up- and down-regulated proteins as revealed by proteomic analysis. Although electrophysiological measures were largely normalized by adulthood in mice lacking microglia, some long-lasting effects in platform-mediated avoidance (PMA) behavior were observed. The manuscript provides evidences for the roles of microglia in synaptic development in the NAc and threat avoidance behaviors. However, most of the data remain observational, and the link between synaptic alterations and behavior impairment remains speculative.

1, Absence of microglia did not impair NAc synaptogenesis (mEPSC, PPR) in adulthood, but disrupted learned and innate avoidance behavior. It is likely that other brain regions, but not NAc contribute to this impaired behavior. It is also intriguing that KO mice exhibit larger shock responses, but lower responses during PMA retrieval (Figure 4B-D). However, the conclusion was drawn from an extremely small sample of animals (n = 3 for KO group and n = 4 for WT/HET group). The different expression levels of GCaMP could also contribute to variability. Did authors observe differences between HET and WT groups? BTW, does HET show changes in synaptogenesis and proteome?

2, Based on the changes in the mEPSC frequency and PPR, the authors concluded that there was a reduction in synapse number (line 118). If this is true, the dendritic spines – postsynaptic compartment of excitatory synapses, should be examined.

3, Line 196: upregulation of AMPA receptor subunit GluR1 in KO mice, could lead to more GluR2-lacking AMPA receptors with faster rise times. Another prominent feature of GluR2-lacking AMPA receptors is they exhibit greater inwardly rectifying currents. Measurement of rectification index might provide additional evidence.

Reviewer #4

(Remarks to the Author)
I co-reviewed this manuscript with one of the reviewers who provided the listed reports. This is part of the Nature Communications initiative to facilitate training in peer review and to provide appropriate recognition for Early Career Researchers who co-review manuscripts.

Version 1:

Reviewer comments:

Reviewer #1

(Remarks to the Author)
The authors have responded to all of my comments. No further concerns.

Reviewer #2

(Remarks to the Author)
The authors have added a considerable amount of experimental data in response to the reviewer comments. These strengthen some aspects of their study, but unfortunately not all data is of convincing quality. Our original comment, that it is difficult to link the data presented in Fig. 1-3 with the rest of the study, remains.

Major Points
Pt 1. Our comment on providing an analysis of synaptic density (shared by all 3 reviewers) was addressed with immunohistochemical analysis of synaptic puncta. The data quality of this experiment is unconvincing, however. The VGLUT2 image shown suggests no difference between genotypes (with VGLUT2+ puncta intensity appearing increased in the KO condition, in contrast to the quantifications), and the PSD95+ puncta appearing oddly dendritic in the KO image. It is unclear to what extent this signal is specific, especially the very bright puncta visible in the PSD95 images, which appear to be artefacts. The lack of pre- and postsynaptic puncta apposition in the representative WT images further exacerbates this concern. Finally, the graphs show major reductions in VGLUT2+ puncta and synapse density of around 50%. These reductions are not apparent in the images shown.

The puncta analysis focuses on excitatory synapses; no data is provided for inhibitory synapses.

Pt 2 and 3. The authors added cells to their mIPSC recordings. This has strengthened the analysis in the sense that some previously reported changes (decay time) are now no longer present, as the authors indicate in their rebuttal, but the number of cells remains low. Fig. 1I shows a trend at 2.5-3mo that could become significant with more observations.

Pt 4 and 5. The tissue-level proteomics data remains problematic in terms of interpretation (this point was also raised by reviewer #1). The authors appear to interpret this data to fit their narrative. Changes in GABA-related components, including downregulation of Gabra1, are interpreted as not necessarily affecting GABAergic synapse number (although no other data is provided to support this statement), whereas other downregulated proteins are presented as potential candidates that may

explain the reduction in synapse density observed with immunohistochemistry.

Another example of selective data interpretation is the sentence (line 205) “We detected more than 3,700 proteins, 229 of which differed significantly across genotype (~10%)”. The small percentage (more like 6%) of changed proteins is then analyzed further. The following statement (line 226) that: “greater than 90% of the proteins enriched at astrocyte synapse ensheathing processes did not differ significantly across genotypes in our NAc tissue dataset” to “suggest that astrocyte structure and capacity to regulate synapse function in the NAc are largely intact in the absence of microglia” is a bit of leap however (even more boldly stated in the rebuttal document: “support the idea that astrocyte ensheathment of synapses is largely preserved in KO mice”). Still 10% of proteins related to ensheathing procceses do change, which is then roughly the same percentage they see in their overall analysis.

A fundamental issue with the proteomic dataset is that this is tissue-level data: it is impossible to draw convincing conclusions from this data for specific cell types or cellular compartments, such as synapses or astrocyte-synapse contacts, as these are underrepresented in tissue extracts. Cross-referencing cannot solve this issue; cell type- or compartment-specific analysis or complementary approaches with higher resolution (such as immunohistochemistry for selected candidate proteins) can achieve this.

Pt 6. The acute, NAc-specific depletion of microglia using CDS is an interesting experiment. The behavioral analysis shows partial overlap with the observation in FIRE mice, though differences are also present. However, the CDS experiment also raises new questions as to what extent this acute, more local manipulation affects morphological and functional synaptic parameters in the NAc in early stages of development. To be able to compare the results of the CDS experiment with the previous dataset, a more systematic comparison of the different parameters analyzed is necessary.

Reviewer #3

(Remarks to the Author)
The authors have sufficiently addressed my questions and I have no further comments.

Version 2:

Reviewer comments:

Reviewer #2

(Remarks to the Author)
We thank the authors for carefully addressing all remaining concerns in their comprehensive rebuttal. The experiments that were added and the textual adjustments that were made have strongly improved the manuscript. We have no further comments, the revised manuscript is ready for publication.

Open Access This Peer Review File is licensed under a Creative Commons Attribution 4.0 International License, which permits use, sharing, adaptation, distribution and reproduction in any medium or format, as long as you give appropriate credit to the original author(s) and the source, provide a link to the Creative Commons license, and indicate if changes were

made.
In cases where reviewers are anonymous, credit should be given to 'Anonymous Referee' and the source.
The images or other third party material in this Peer Review File are included in the article's Creative Commons license, unless indicated otherwise in a credit line to the material. If material is not included in the article's Creative Commons license and your intended use is not permitted by statutory regulation or exceeds the permitted use, you will need to obtain permission directly from the copyright holder.
To view a copy of this license, visit https://creativecommons.org/licenses/by/4.0/

We thank the reviewers for their helpful suggestions and words of encouragement. Reviewers noted that our *“data are interesting and add to the now large literature indicating microglia play important roles in normal brain development, especially during discrete developmental windows that are brain region and time point-dependent.”* Also, they noted that ours *"is a timely study that is clearly written and presented.”* In this revised manuscript, we clarified points of confusion, added discussion points and carried out many of the suggested experiments. Briefly, for the revision:

- We performed histological analysis of excitatory synapse number at P22, when we observed functional changes in synapses in FIRE mice.
- We performed morphological assessments of astrocytes in KO and WT/HET control mice.
- We increased the sample size for the mIPSC recordings.
- We clarified the methods and other key details for the proteomics experiments. We also modified the plots showing key findings for clarity.
- We increased the sample size for the fiber photometry experiments, strengthening our conclusions.
- We transiently depleted NAc microglia in WT mice during the second postnatal week using clodronate disodium salt and observed alterations in adult threat avoidance behaviors.
- We quantified microglial density in heterozygous FIRE mice.

We believe these changes and our new results greatly enhance the study. Below, we provide a point-by-point response to the reviewer questions, comments and concerns.

Response to Reviewer #1

The authors examined the impact of microglial deletion in the FIRE mice on NAc synapse dynamics and function, reporting decreases in the frequency of mEPSCs at P20-22 along with proteomic changes consistent with a change in synapse development. FIRE mice also show changes in threat avoidance behavior and anxiety, as well as altered NAc activity during the performance of these tasks. These data are interesting and add to the now large literature indicating microglia play important roles in normal brain development, especially during discrete developmental windows that are brain region and time point-dependent. There are some leaps in conclusions by the authors that are not necessarily supported by the data, along with some confusing aspects of the paper that should be addressed in revision.

Major:

1. The authors conclude that the lack of microglia leads to a reduction in excitatory synapse number based on electrophysiology, which is convincing, but should be supported with histological assessments of synapse number.

We agree that analysis of synapse number using distinct methodology will strengthen overall conclusions about the impact of microglial absence on NAc synapse development. To address this reviewer concern, we performed immunohistochemistry for VGLUT2 and PSD95 and used confocal imaging and analysis in Imaris to analyze abundance of excitatory synapses in NAc at P22, the time point when we observed the largest reduction in mEPSC frequency in KO mice. We found reduced numbers of VGLUT2+, PSD95+, and VGLUT2/PSD95+ colocalized puncta in KO compared to WT mice (New Figure 2), supporting the conclusion that microglial absence is

associated with deficits in NAc synaptogenesis (see also response to reviewer 2, point 1 and reviewer 3, point 2).

2. The authors further conclude that the observed effects on synapse function are not due to changes in astrocytes based on proteomics data in which secreted proteins are not significantly altered. This is a considerable leap, given that astrocytes perform many functions to support synapse function aside from secreted proteins, as the authors themselves point out, including structural and morphological impacts e.g. via adhesion proteins that may not be changed in total number, but in location/contact with synapses. Morphological assessments of astrocytes should be completed to investigate these possibilities.

We fully agree that analysis of our proteomic data does not represent a comprehensive assessment of potential astrocytic effects on synapse function. It is worth noting that, in addition to astrocytic proteins that promote synaptogenesis, many other astrocyte proteins that would impact ongoing synapse function were also unchanged in KO mice. Glutamate and GABA transporters on astrocyte processes play a key role in terminating synaptic signals and perturbation of astrocyte synapse-ensheathing processes would be expected to alter decay kinetics of synaptic currents. We did not observe alterations in mEPSC or mIPSC kinetics (Figure S2C), and levels of Slc1a2 (GLT1, glutamate transporter), Slc1a3 (GLAST, glutamate transporter), and Kcnj10 (KIR4.1, potassium channel) in our proteomic dataset did not differ across genotypes.

To address the reviewer's concern and further analyze the status of astrocytes in KO mice, we used viral approaches to achieve sparse labeling of NAc astrocytes and carried out detailed morphological analysis. We performed retro-orbital injections of PHPeB::GfaABC1D-Cre-4x6T, PHPeB::CAG-flex-lck-smV5-4x6T, and PHPeB::CAG-flex-GFP-4x6T at P12 and analyzed astrocyte morphology at P22, when the most significant reductions in mEPSC frequency were observed. This analysis revealed that astrocyte territory size, number of primary branches and average length of primary branches did not differ between KO and WT/HET mice. Results of these experiments have been added in Figure S4 D,E. In the results section, we now highlight that this preserved astrocyte morphology aligns with our observation that mEPSC and mIPSC decay kinetics are comparable in KO and WT mice.

As a final approach for assessing status of astrocyte synapse-ensheathing processes, we examined the status of proteins found to be enriched at astrocyte-synapse contacts. In Soto et. al 2023, the authors used GLT1-targeted BioID2 proximity biotinylation to label and identify proteins found to be enriched at astrocyte synapse-ensheathing processes within the striatum. To refine these lists of proteins, the authors compared to proteins identified at other astrocytic subcellular compartments (Cx43 - gap junctions, Aqp4 - blood vessel end feet, Kir4.1 - sites for potassium homeostasis, Ezr - structural protein within fine processes) to identify 206 proteins unique to these GLT1+ synaptic processes. They also compared GLT1-targeted BioID2 identified proteins to proteins identified with the cytosolic astrocyte BioID2 to identify 528 proteins enriched at GLT1+ synapse ensheathing astrocyte processes. In our NAc tissue proteomics, we detected 143 / 206 and 370 / 528 proteins in these lists. In our dataset, greater than 90% of these proteins that are enriched at astrocyte synapse-ensheathing processes did not differ significantly between KO and WT mice. These analyses have been added to Figure S5C and support the idea that astrocyte ensheathment of synapses is largely preserved in KO mice.

3. "Thus, we identified key signaling proteins that may play previously unappreciated roles in microglia-mediated synapse formation in the NAc and that merit dedicated follow-up studies".

Given that this conclusion is entirely speculative, the authors should either include functional follow up studies, or omit this discussion.

We agree with the reviewer that the molecules we've highlighted require functional follow-up studies before any clear conclusions can be drawn regarding their role in supporting NAc synaptogenesis and/or synapse maturation. However, we respectfully disagree that, in the absence of functional follow-up studies, this component of our study should be removed. The vast majority of transcriptomic and proteomic studies highlight groups of molecules or signaling pathways that may merit follow-up studies without necessarily carrying out those studies. Robust functional interrogation of panels of candidate genes / proteins is a substantial undertaking that, in the case of microglia, frequently requires generation of novel transgenic mouse lines. In our view, the authors of -omics studies should play a role akin to that of literature review articles. You expect the author of a review article to sift through abundant literature and help you, as the reader, extract the key take home points and important future directions for the field. Similarly, authors of -omics studies are generally expected to sift through a large dataset and highlight key elements that, in their opinion, merit attention of the field moving forward.

We would also like to respectfully push back against the idea that the molecules we highlight are "entirely speculative." To identify the molecules of interest, we carried out functional annotation to identify secreted molecules and cell surface molecules that could have a signaling role. We then cross interrogated that list of molecules against 4 publicly available RNAseq datasets to provide information about levels of microglial expression, enrichment in microglia relative to other CNS cell types, and enrichment in NAc/striatal microglia compared to microglia in other brain regions.

To address the reviewer's concern, we have:

- In the final paragraph of the introduction we have modified the sentence "*To investigate potential molecules that drove these changes, we leveraged tissue proteomics and identified altered expression of microglial degradative enzymes and membrane proteins that may contribute to synapse formation*." It now reads "*We leveraged tissue proteomics to reveal how absence of microglia changes the landscape of synaptic proteins in the developing NAc and to show that the synaptic changes we observed are unlikely to be caused by perturbation of astrocyte synapse-ensheathing processes, astrocyte synaptogenic cues, or adhesive molecules that support synapse formation*."
- In the first paragraph of the discussion, we removed the phrase "*suggesting that lack of microglia-derived cues may underlie observed deficits in synapse formation*". We also modified the final sentence of the same paragraph to read "*Moreover, we highlight candidate proteins that may mediate microglial support of NAc synaptogenesis and that merit dedicated follow-up functional studies.*"
- In paragraph 3 of the discussion, we modified the concluding sentence from "*Thus, we identified key signaling proteins that may play previously unappreciated roles in microglia-mediated synapse formation in the NAc and that merit dedicated follow-up studies*" to read "*Cell-specific manipulation of these molecules in future studies can test whether they play a role in microglial support of synapse formation in the developing NAc*."
- We carried out additional cross-interrogation of our proteins of interest with 4 publicly available proteomic datasets, to provide further evidence that these molecules are expressed by microglia at the protein level and to provide additional insight into enrichment of these molecules in microglia relative to other CNS cell types. In the revised Figure 3G, we have elected to focus more tightly on the molecules for which

there was strong evidence of protein-level expression in microglia. Hence, we highlight 5 proteins that may merit dedicated follow-up studies. We have also focused only on the microglial transcriptomic studies that provide information regarding potential regional differences in microglial expression of these molecules.

Minor/clarification:
1. Lines 126-130 – Shouldn't this read "suggesting that microglia do NOT significantly impact relative abundance of these receptors at NAc glutamatergic…"?

Thank you for catching this mistake. We have corrected it.

2. Were recordings done in core vs shell of NAc, or no distinction? Same for proteomic dissections?

We used the anterior commissure to guide our recordings, targeting cells that were in the core or on the core/shell border, typically just medial and inferior to the commissure. Our proteomic study and histological analyses captured both core and shell to ensure we had enough material. We have clarified these points in the methods.

3. For Fig. 2C-D. Please clarify the different columns. Are these technical replicates?

In the revised manuscript, these data are now part of Figure 3. We have modified how these data are presented to more effectively represent the degree of sample-to-sample variation present (please see response to Reviewer 2, point 4). Data are now presented as bar graphs with data points for individual biological replicates (mice) overlaid on bars.

Response to Reviewer #2

This study by Gongwer, Etienne et al. investigates the consequences of a lack of microglia on the nucleus accumbens (NAc). Using microglia-lacking FIRE mice, the authors first investigate synapse function and find a decrease in mEPSC frequency in NAc neurons, accompanied by an increase in presynaptic release probability, which are most pronounced in the third postnatal week (P20-22) and normalize in adulthood. The authors then perform proteomics of microdissected NAc tissue from P20-22 FIRE mice and find changes in levels of synaptic proteins and pathways related to synaptic processes. Behavioral testing of adult FIRE mice shows defects in threat avoidance behavior and heightened anxiety. In vivo calcium imaging of NAc neurons in FIRE mice reveals changes in NAc neuron activity that can be linked to these behavioral changes. Together, the results show that microglia play a role in establishing NAc synaptic connectivity important for threat avoidance behavior. This is a timely study that is clearly written and presented. However, the results as they are currently presented are too preliminary and do not support strong conclusions. It is difficult to link the data present in Figures 1 and 2 with the data presented Figures 3-4. Without further validation, the results from Figure 2 remain speculative. With additional experimental evidence this would be a much stronger study.

We thank the reviewer for their constructive feedback and for recognizing the timeliness of our study. We have carried out additional experiments and analyses to address the reviewer's concerns.

Major

1. Throughout the paper, the authors claim that excitatory synaptogenesis is impaired in FIRE mice (e.g. Page 4 '…the prominent reduction in excitatory synapse number…'). This claim is based on their observation that mEPSC frequency is decreased, but release probability is increased in P20-22 FIRE mice. However, there is no analysis of synapse density provided in the study.

We performed immunohistochemistry, confocal imaging, and Imaris-based analysis to analyze synapse number in the NAc and have added these analyses as a new Figure 2 in the revised manuscript. Focusing on glutamatergic synapses at P22, when we observed the most prominent reductions in mEPSC frequency, we found a reduction in VGLUT2+, PSD95+, and colocalized VGLUT2/PSD95+ puncta in KO compared to WT mice (Figure 2). These data support the conclusion that microglial absence is associated with deficits in NAc synaptogenesis (see also response to reviewer 1, point 1 and reviewer 3, point 2).

2. The mIPSC measurements are based on a very low number of neurons and animals and do not allow for clear conclusions. This is important to address because the proteomics analysis in Figure 2E shows that the top downregulated term is related to inhibitory synapses. These recordings need to be repeated, and the number of mice/neurons needs to be increased. These observations then need to be linked to synapse density measurements as mentioned above.

To address these concerns, we increased the sample size for the mIPSC recordings (P22: WT: n=11 cells from 5 mice; KO: n=12 cells from 5 mice; 2.5-3 mo. WT: n=11 cells from 5 mice; KO: n=12 cells from 5 mice). We did not observe any differences in mIPSC frequency or amplitude (Figure 1), but observed a decrease in mIPSC rise time in adult KO mice. As we observed no changes in mIPSC frequency, we focused our immunohistochemical analyses on excitatory synaptic markers.

In our proteomics pathway analyses (carried out in Metascape) the pathway "GABA synthesis, release, reuptake and degradation" was flagged due to 13 proteins from our dataset that were significantly downregulated in KO mice (Reviewer Figure 1). A number of these protein hits were synaptic or metabolic proteins that are not unique to GABA synapse function. Proteins that *were* specific to GABA synapses included enzymes needed for GABA synthesis, GABA transporters, and one GABA receptor subunit. Decreases in these proteins would not necessarily be expected to impact the <u>number</u> of functional GABA synapses, and, hence, are not inconsistent with our electrophysiological observation of similar mIPSC frequency in WT and KO mice. In support of this interpretation, the majority of detected GABA receptor subunits as well as the GABA scaffolding protein gephyrin were unaltered in the FIRE KO mice (Reviewer Figure 2 and Figure 3D). The observed decreases in GABA synthetic enzymes and the vesicular GABA transporter (Slc32a1) do raise the possibility that GABA synapses in KO mice may have reduced capacity for repetitive / sustained activation, an idea that would be worth exploring in future studies.

In this revision, we have incorporated the above points into the discussion and have added Supplementary Table 2, which contains the complete metascape annotations and lists of specific proteins from our dataset that caused individual pathways to be flagged as significant. This will enable readers to easily view the specific proteins underlying individual pathways.

Proteins from our dataset that are found in "GABA synthesis, release, re-uptake and degradation"

Protein	t-test	FC	Expr. level	location	notes
Gad1	0.04	-0.44		GABA synapse	glutamic acid decarboxylase; produces gamma-aminogutyric acid from L-glutamic acid
Gad2	0.00	-0.62		GABA synapse	glutamic acid decarboxylase; produces gamma-aminogutyric acid from L-glutamic acid
Slc32a1	0.03	-0.40		GABA synapse	VGAT - transporter for loading GABA into synaptic vesicles
Slc6a11	0.01	-0.85		GABA synapse	Astrocytic sodium dependent re-uptake transporter for GABA (GAT3 / VIAAT)
Gabra1	0.01	-0.64		GABA synapse	gamma-aminobutyric acid type A receptor subunit alpha 1
Pnkd	0.04	-2.03		pre-synaptic;	interacts with proteins that regulate neurotransmitter release; expressed by variety of cell types
Grm1	0.03	-2.51		post- & extra-synaptic	glutamate metabotropic receptor 1
Glul	0.01	-0.25		astrocyte cytosol	glutamine synthetase; important for glutamate and GABA metabolism and synthesis
Aldh5a1	0.04	-0.20		mitochondria	mitochondrial NAD(+)-dependent succinic semialdehyde dehydrogenase
Hagh	0.02	-0.39		mitochondrial	Hydroxyacylglutathione Hydrolase
Ndufs6	0.00	-0.87		mitochondria	NADH:Ubiquinone Oxidoreductase Subunit S6
Aldoa	0.05	-0.12		cytosolic	aldolase A; fructose-bisphosphate aldolase - enzyme that plays critical role in glycolysis
Hexb	0.00	-4.25		microglial lysosome	included by metascape due to roles in small molecule catabolic processes

Reviewer Figure 1. NAc tissue proteins that were significantly down-regulated in KO compared to WT mice and that are members of the pathway "GABA synthesis, release, re-uptake and degradation." P-value (t-test) and fold change (FC) are shown. Expression level heatmap indicates abundance of the protein relative to all detected proteins in NAc tissue. Synaptic proteins that are not necessarily specific to GABAergic synapses are noted in blue font. Metabolic proteins that are important for synaptic function, but also other aspects of cell metabolism, are noted in red font.

GABA synapse proteins that were not differentially expressed across genotype

Protein	t-test	FC	Expr. level	location	notes
Gabra2	0.70	1.01		GABA synapse	gamma-aminobutyric acid type A receptor subunit alpha 2
Gabra3	0.40	1.02		GABA synapse	gamma-aminobutyric acid type A receptor subunit alpha 3
Gabrb1	0.59	0.37		GABA synapse	gamma-aminobutyric acid type A receptor subunit beta 1
Gabrb2	0.51	0.21		GABA synapse	gamma-aminobutyric acid type A receptor subunit beta 2
Gabrb3	0.30	-0.45		GABA synapse	gamma-aminobutyric acid type A receptor subunit beta 3
Gphn	0.67	-0.09		GABA synapse	gephyrin

Reviewer Figure 2. GABA receptor subunits and scaffolding proteins from NAc tissue proteomics that did not differ in abundance between KO and WT mice. P-value (t-test) and fold change (FC) are shown. Expression level heatmap indicates abundance of the protein relative to all detected proteins in NAc tissue.

3. There is a significantly faster mIPSC rise time in adult FIRE mice and increased mIPSC decay time in P20-22 FIRE mice (Figure S2C). The authors speculate that this could be due to a change in GABAAR subunit composition. This should be validated.

When we increased the sample size in electrophysiological experiments targeting GABAergic synapses, as the reviewer suggested in point 2, we found that the only difference across genotypes was that mIPSCs in KO adults have a faster rise time. There were no longer significant differences in decay time in P20-22 KO mice. We have removed speculation of GABAAR playing a role in altering the decay constant from the text.

4. Proteomics data (Figure 2, S4) lacks a detailed description and quality controls: it is not clear from the methods how the samples were analyzed (e.g. which mass spectrometer was used), how many proteins were identified, how many proteins were significant and what the variability between samples was. Figure 2C and D suggest a surprising variability between samples within conditions; a PCA plot of samples and plot of sample coefficient of variation between conditions can be provided. The accompanying supplemental table lacks information on p-value and fold-change in protein levels.

We thank the reviewer for pointing out that additional methodological information was needed. We made the following changes:

- We have provided a more detailed description of proteomics data acquisition, including use of an Orbitrap Fusion Lumos Tribrid mass spectrometer (Thermo Fisher Scientific), quality control, and analysis in the methods.
- We added a sentence to the results section describing that “We detected more than 3,700 proteins, 229 (~10%) of which differed significantly across genotype.”
- We apologize for the confusion in Figure 2C,D (now Figure 3C,D). We have modified the presentation of this data to use bar graphs rather than heatmaps. This allows us to directly display $\log_2$ intensity, rather than z-scores of intensity, which indicates more directly the degree of sample-to-sample variability.
- We have added a violin plot to Figure S5A showing coefficient of variation across samples for all proteins to clarify that WT and KO mice exhibit similar degrees of sample-to-sample variation.
- We added two columns to Table S1 reporting p-value and fold change across genotype for each protein.

5. The interpretation of the authors of their proteomic data is unclear and remains speculative. They observe significant changes in some synaptic proteins (e.g. glutamate receptor subunit Gria1 and metabotropic glutamate receptor Grm1) yet conclude that ‘protein components necessary to form functional synapses are still adequately produced in KO mice’. This is not supported by the data, most downregulated GO terms relate to the synapse. The authors seem to contradict this conclusion in the next paragraph where they mention that SynGO analysis ‘further supported the observation that microglial absence impacts numerous synapse relevant proteins’. The relevant synaptic proteins should be indicated in the volcano plot to allow the reader to judge the biological significance of these changes; this is difficult to judge from the normalized plots in Figure 2C and D. It is also not clear how the highlighted proteins in Figure 2C and D were selected, is this an exhaustive list? For example, the GO analysis in Figure 2E shows multiple synaptic terms in the downregulated list related to synaptic structure and function: what proteins are in these terms and where do they fall on the volcano plot?

The proteomics data are now in Figure 3. Thank you for pointing out how presentation of these data could cause confusion. To address this reviewer concern, we have made the following changes:

- We labeled synaptic proteins on a separate volcano plot so readers can clearly read the labels (Figure S5D).
- We have re-written several paragraphs of the results section to better describe the rationale for the molecules highlighted in Figures 3C,D and to clarify our interpretations of these data.
- We have modified the figure legend for Figure 3 to further clarify rationale for proteins highlighted in 3C and 3D.

- We have added Supplementary Table 2, which contains the complete metascape annotations and lists of specific proteins from our dataset that caused individual pathways to be flagged as significant.
- We have modified the discussion of how our proteomic, histological, and electrophysiological findings relate to one another.

6. It remains difficult to interpret how the observations in Figure 1-2 are linked to the results in Figure 3-4. It appears that the authors suggest that defects in synaptic development in the absence of microglia result in lasting defects in behavior later in life. However, microglia are also absent in adult FIRE mice, and the changes in NAc neuronal activity observed at this stage may result from an effect of microglia on neuronal activity, independent of synaptic changes during postnatal development.

This is an important point. To better link these two sets of findings, we performed new experiments in which we depleted NAc microglia around P15 in WT mice, when microglial density peaks (Figure 1A), and then performed behavioral analyses in adulthood (Figure 6). To achieve transient microglial depletion, we injected clodronate disodium salt (CDS) or a saline control into the NAc of WT mice at P10. We observed a dramatic reduction in NAc microglia at P15 and a return to normal microglial density by P21. In adulthood, CDS-treated mice exhibited anxiety-like behaviors in the elevated zero maze (Figure 6H), but enhanced learning during PMA (Figure 5I,J), similar to what we observed in the FIRE mice. However, CDS-treated mice did not exhibit changes in PMA retrieval, suggesting that the changes in threat memory-guided behaviors we observed in FIRE mice (Figure 6I,J) arise from changes occurring outside of the third postnatal week. Based on these findings, we also added more discussion of how changes in synapse development may lead to later changes in behavior and circuit activity.

Minor

1. The statements in the Discussion 'suggestion that lack of microglial-derived cues may underlie observed deficits in synapse formation', and 'we identified key signaling proteins that may play previously unappreciated roles in microglia-mediated synapse formation in the NAc and that merit dedicated follow-up studies' are not supported by the evidence provided.

We have revised the introduction and discussion sections to tone down these claims and emphasize future studies that are needed. Please see also the response to Reviewer 1, point 3.

2. The number of animals/cells analyzed is not always clear from the figure legends (e.g. PPR experiments, Figure 1G). Please indicate these.

We updated the figure legends so all now have this information.

3. Figure 1G: please explain rationale for including heterozygotes in control group: were these validated to not show changes in microglia number at the timepoints investigated?

The reviewer is correct. Heterozygous mice were included in the control group because they did not show changes in microglia density. We have added a new supplemental figure (Figure S1) to show this.

4. Figure S2: what was the reason not to include mIPSC data from P8-10 and P14-16 mice?

To manage the scope of experiments, we focused on P20-22, the period of development when we had observed changes in excitatory synapse number and function, and adulthood, when we

observed behavioral changes in FIRE mice. Additionally, mIPSC frequency was much lower than mEPSC frequency at P20-22 across genotypes, suggesting inhibitory synapse formation prior to this point was limited and we therefore did not pursue mIPSC recordings at earlier timepoints.

5. Figure 2G - pipeline to find interesting candidates of how microglia could contribute to synapse formation, please explain this pipeline in more detail. The +++ and ++ in the table are subjective. Please also select only NAc RNAseq data and not whole brain data in your pipeline. It is not clear why the expression must be higher compared to other brain regions, is this because of NAc region specificity?

In the revised manuscript, these data are now in panel 3G. We removed the columns with subjective representations of expression (+++, etc.). We also carried out additional cross-interrogation of our proteins of interest with 4 publicly available proteomic datasets, to provide further evidence that these molecules are expressed by microglia at the protein level and to provide additional insight into enrichment of these molecules in microglia relative to other CNS cell types. Based on these data, we have elected to focus more tightly on the molecules for which there was strong evidence of protein-level expression in microglia. Hence, we highlight 5 proteins that may merit dedicated follow-up studies. For the RNAseq data in this panel, we have also focused only on the microglial transcriptomic studies that provide information regarding potential regional differences in microglial expression of these molecules. We did not consider elevated expression in striatum / NAc relative to other brain regions as a *necessary* criteria for being considered as a candidate microglial synaptogenic molecule, but felt that this information provided useful insight into potential regional specificity of the action of these molecules. (see also response to reviewer 1, point 3).

6. Figure 2: some validation of the proteomics data would enhance the study (e.g. the changes in Gria1 or Grm1 levels).

We agree that validation of proteomics data is important, particularly for any follow-up studies targeting specific proteins found to be altered via tissue proteomics. Our original manuscript included histological validation of the proteomics finding that GFAP expression is elevated in KO mice compared to control mice at P20-22 (Figures 3 and S4). For this manuscript revision, to manage the scope of the study, we focused our efforts on histological validation of a decreased number of synapses in KO mice at this timepoint. We found decreased number of $VGLUT2^+$ and $PSD95^+$ puncta in KO mice compared to controls (New Figure 2), consistent with our electrophysiological findings (see also response to reviewer 1, point 1, reviewer 2, point 1, and reviewer 3, point 2).

7. Figure S4: please provide an explanation of how the astrocyte-specific proteins were identified, we could not find this in the Methods. The text on page 4 is misleading: ‘the majority of astrocyte enriched proteins identified by astrocyte-specific proteomics were not significantly altered across genotype’ – if understood correctly the authors cross reference their tissue-level NAc proteomics dataset with another study to identify putative astrocytic proteins in their dataset. This should be clarified.

These data are now Figure S5. We have modified the text of the results to better describe the Soto et. al 2023 study from which we drew these astrocyte-enriched proteins. This section of the results now reads:

"Well-characterized astrocyte proteins that shape glutamate clearance and potassium buffering at the synapse, including Slc1a2 (GLT1, glutamate transporter), Slc1a3 (GLAST, glutamate transporter), and Kcnj10 (KIR4.1, potassium channel), were also unaltered in KO mice, supporting electrophysiological observations that mEPSC and mIPSC decay kinetics did not differ across genotypes. For a more unbiased evaluation of astrocyte-relevant proteins in our dataset, we also examined proteins highly expressed by striatal astrocytes that were identified by cytosolic astrocyte TurboID, as well as proteins that are highly expressed at striatal astrocyte processes that ensheath synapses (GLT1-targeted-BioID2)[40]. The majority of proteins enriched in striatal astrocyte cytosol did not differ in abundance between KO and WT mice (Table S1, Figure S5B). Similarly, greater than 90% of the proteins enriched at astrocyte synapse ensheathing processes did not differ significantly across genotype (Figure 5SC)."

We have also added a description of the source and utilization of these protein lists to the methods.

8. Figure S4A: S1PR1 is upregulated in KO. This protein has been shown to regulate the secretion of astrocyte-derived cues (Singh et al. 2022 Glia). Please comment on this observation.

We included discussion of this point, now related to Figure S5B.

"*Although we did not observe changes in detected astrocyte-derived synaptogenic cues (Gpc4, Gpc6, Sparcl1), some astrocyte-derived synaptogenic cues were not detected via proteomics (e.g. Tsp4, Thbs1, Thbs2, Sparc, Chrdl1) and we cannot exclude that these factors are present in the developing NAc at low levels and are perturbed by microglial absence. Moreover, S1pr1, which we found to be upregulated in KO mice, is expressed in perisynaptic astrocyte processes and regulates astrocyte morphology. In neuron-astrocyte co-cultures, activation of S1pr1 increased astrocyte expression of synaptogenic cues such as Sparcl1 and Tsp4, an effect that required neuron-astrocyte contact. Hence, additional studies will be needed to fully define the status of astrocyte support for synaptogenesis in the absence of microglia.*"

9. Figure 3E: the data seems to be ordinal (having fixed values). This should not be an unpaired t-test to compare the means, but a Mann Whitney U test.

Thank you for pointing this out. We have updated the analysis to use a Mann-Whitney U test instead of an unpaired t-test.

Response to Reviewer #3:

In this manuscript, Gongwer & Etienne et al take advantage of a genetically modified mouse that lacks microglia to elucidate the role of microglia in regulating the synaptic landscape of the developing NAc and adult behavioral repertoires. The authors showed that absence of microglia blunts NAc excitatory synaptogenesis, which was accompanied by many up- and down-regulated proteins as revealed by proteomic analysis. Although electrophysiological measures were largely normalized by adulthood in mice lacking microglia, some long-lasting effects in platform-mediated avoidance (PMA) behavior were observed. The manuscript provides evidences for the roles of microglia in synaptic development in the NAc and threat avoidance behaviors. However, most of the data remain observational, and the link between synaptic alterations and behavior impairment remains speculative.

We thank reviewer 3 for their helpful comments. To address concerns about the link between synaptic alterations and behavior impairment, we used clodronate disodium salt to perform temporary depletion of NAc microglia during the window thought to produce the synaptic alterations (Figure 6). We then performed behavioral analyses in adulthood, when microglia had repopulated the tissue. We found that developmental depletion of NAc microglia reproduced some of the behavioral effects we had observed in KO mice (Figure 6). From this, we can more strongly conclude that NAc microglia function in that particular window of development is critical for shaping adult behavior (see also response to reviewer 2, point 6).

1, Absence of microglia did not impair NAc synaptogenesis (mEPSC, PPR) in adulthood, but disrupted learned and innate avoidance behavior. It is likely that other brain regions, but not NAc contribute to this impaired behavior. It is also intriguing that KO mice exhibit larger shock responses, but lower responses during PMA retrieval (Figure 4B-D). However, the conclusion was drawn from an extremely small sample of animals (n = 3 for KO group and n = 4 for WT/HET group). The different expression levels of GCaMP could also contribute to variability. Did authors observe differences between HET and WT groups? BTW, does HET show changes in synaptogenesis and proteome?

To address this concern, we increased the sample size for the fiber photometry experiments to n = 8 per group. The additional recordings strengthened our conclusions that KO mice exhibit larger calcium elevations in response to shock but lower calcium responses during PMA retrieval. We also added new fiber photometry recordings during the EZM, showing increased NAc activity in KO mice following entry into the open arms, which aligns with the increased anxiety levels we had observed in KO mice. Because some measurements showed an increase in activity in KO mice and others showed a decrease, we believe it is highly unlikely that the effects we observed were due to differences in GCaMP expression.

We did not observe differences in NAc activity between HET and WT groups. To further validate the grouping of WT and HET mice in the control group, we performed new analysis of microglia density, showing that HET and WT are indistinguishable (Figure S1). Additionally, we have indicated HET mice with triangles in all plots in which HET mice were included in the control group. All plots, including electrophysiological, histological, behavioral, and photometry experiments, showed overlapping distributions between WT and HET mice. We therefore believe that plotting these two groups together was justified for our analyses.

2, Based on the changes in the mEPSC frequency and PPR, the authors concluded that there was a reduction in synapse number (line 118). If this is true, the dendritic spines – postsynaptic compartment of excitatory synapses, should be examined.

We agree with the reviewer that an independent approach for examining synapse number was needed to strengthen this conclusion. To histologically investigate changes in synapse number, we performed immunohistochemistry for pre- and postsynaptic markers in the NAc at P22, confocal imaging, and Imaris-based analysis. We found decreased density of VGLUT2+, PSD95+, and VGLUT2/PSD95+ colocalized puncta in KO compared to WT mice (New Figure 2), which supports our finding that miniature EPSC frequency is significantly reduced in KO mice at the same age (see also responses to reviewer 1, point 1 and reviewer 2, point 1).

3, Line 196: upregulation of AMPA receptor subunit GluR1 in KO mice, could lead to more GluR2-lacking AMPA receptors with faster rise times. Another prominent feature of GluR2-lacking AMPA receptors is they exhibit greater inwardly rectifying currents. Measurement of rectification index might provide additional evidence.

We agree that assessing rectification index would be an excellent approach to further probing whether there are differences in AMPAR subunit composition between KO and WT mice. For this revision, we chose to focus on confirming changes in synapse number in KO mice and determining whether transient NAc microglial depletion during development resulted in behavioral changes in adulthood. With this focus on developmental trajectories - revealing initial synaptic changes and later circuit and behavioral changes, we believe further kinetics analysis is beyond the scope of this study. However, we added the recommendation that "*Future studies should investigate the effects of microglial absence on AMPA receptor rectification to provide further insight into the impact on glutamate receptor subunit composition*" to the discussion (see also response to reviewer 2, point 6).

We thank the reviewers for their helpful suggestions. Reviewers 1 and 3 had no additional comments. Below we provide point by point responses to Reviewer 2.

Pt 1. The VGLUT2 image shown suggests no difference between genotypes (with VGLUT2+ puncta intensity appearing increased in the KO condition, in contrast to the quantifications). The PSD95+ puncta appearing oddly dendritic in the KO image. It is unclear to what extent this signal is specific, especially the very bright puncta visible in the PSD95 images, which appear to be artefacts.

We thank the reviewer for this observation. In the original manuscript, we quantified the abundance of VGLUT2 puncta, but not puncta intensity or size. To clarify this point, we have revised **Figure 2** to better indicate how we identified and tagged VGlut2 and PSD95 puncta for quantification of puncta abundance. We also added new analyses of VGLUT2 and PSD95 puncta intensity and size, showing that VGlut2 puncta trend toward higher intensity in KO mice, as the reviewer perceived via the fluorescence images.

To address the concern regarding the appearance of PSD95 staining, we added a new supplementary figure (**Figure S4**) that includes multiple representative fields of view from mice of each genotype. The dendritic-like PSD95 staining pattern was observed to varying degrees in all KO animals and was not observed in CTR animals. Importantly, our quantification is based on puncta number and VGLUT2/PSD95 colocalization, which are not affected by the presence of this diffuse staining pattern. In both the text of the results and the discussion, we now note that we observed altered PSD95 distribution in KO animals that may reflect changes in PSD95 trafficking or localization. We emphasize that this interpretation is speculative and does not affect the quantitative analyses or the conclusions of the study.

The lack of pre- and postsynaptic puncta apposition in the representative WT images further exacerbates this concern.

Thank you for pointing this out. Because the nucleus accumbens receives glutamatergic inputs from both subcortical VGLUT2-positive axons and cortical VGLUT1-positive axons, we do not expect all PSD95 puncta to be apposed to VGLUT2 puncta. Consistent with this, our quantitative analysis found that approximately 45–60% of PSD95 puncta were associated with VGLUT2-positive terminals, which is in line with previous reports showing comparable densities of VGLUT1- and VGLUT2-expressing inputs in the rat NAc (10.1016/j.neuroscience.2017.03.014). We have added text to the results section to better contextualize the % colocalization we are observing.

In addition, our puncta detection criteria were intentionally stringent to minimize false-positive assignments. As a result, some true synaptic contacts may not meet our colocalization threshold. However, as the same analysis parameters were applied uniformly across all samples and experimental groups, this will not impact our findings or interpretations. To clarify this point, we have revised **Figure 2** to better illustrate representative VGLUT2–PSD95 appositions.

Finally, the graphs show major reductions in VGLUT2+ puncta and synapse density of around 50%. These reductions are not apparent in the images shown.

Thank you for this observation. To address this concern, we have added annotated ("tagged") versions of the images in **Figure 2** that identify the puncta and synaptic contacts included in the

analysis. These annotated images more clearly illustrate the reduction in VGLUT2-positive puncta and synapse density observed in the experimental group.

The puncta analysis focuses on excitatory synapses; no data is provided for inhibitory synapses.

This is an important point. To address this concern, carried out additional experiments to quantify GABAergic synaptic puncta using similar strategies as those employed for glutamatergic synapses. We found no significant differences in puncta number between genotypes, consistent with our electrophysiological recordings. These data have now been added to the manuscript (**Figure 2** and corresponding Results section) to provide a more complete assessment of synaptic changes across both excitatory and inhibitory synapses.

Pt 2 and 3. The authors added cells to their mIPSC recordings. This has strengthened the analysis in the sense that some previously reported changes (decay time) are now no longer present, as the authors indicate in their rebuttal, but the number of cells remains low. Fig. 1I shows a trend at 2.5-3mo that could become significant with more observations.

The reviewer raises an important point regarding the possibility that the trend observed at 2.5–3 months could become significant with additional observations. To address this concern, we performed additional mIPSC recordings in this age group. The revised data set now includes 14 cells recorded from 6 WT mice and 15 cells recorded from 6 KO mice (**Figure 1I**). With the increased sample size, we still detected no significant group differences in mIPSC frequency. These data therefore support our conclusion that the absence of microglia does not produce a measurable effect on inhibitory synaptic transmission in the NAc at the ages examined.

Pt 4 and 5. The tissue-level proteomics data remains problematic in terms of interpretation (this point was also raised by reviewer #1). The authors appear to interpret this data to fit their narrative.

Changes in GABA-related components, including downregulation of Gabra1, are interpreted as not necessarily affecting GABAergic synapse number (although no other data is provided to support this statement), whereas other downregulated proteins are presented as potential candidates that may explain the reduction in synapse density observed with immunohistochemistry.

We appreciate the reviewer's point and want to ensure that our presentation of proteomics data is balanced and does not have selective "over-focus" on specific portions of the dataset. For Gabra 1, changes in receptor subunit expression can occur independently of changes in inhibitory synapse number. Consistent with this interpretation, our revised manuscript now includes both inhibitory puncta analysis and an expanded electrophysiological dataset, neither of which provide evidence for altered GABAergic synapse number. We have revised the discussion to more clearly distinguish between protein expression changes and synaptic density measures, and to point to additional experiments that would be needed to link proteomic data to features of synaptic function.

We also added the following sentence to the results section describing pathway analyses: "GABA synthesis, release, reuptake, and degradation" was a top hit on Metascape analysis of downregulated proteins, suggesting that, although GABAergic synapse number was normal via electrophysiological and histological experiments in KO mice (Figures 1,2), these synapses may exhibit functional deficits under conditions of heightened activity."

Another example of selective data interpretation is the sentence (line 205) "We detected more than 3,700 proteins, 229 of which differed significantly across genotype (~10%)". The small percentage (more like 6%) of changed proteins is then analyzed further. The following statement (line 226) that: "greater than 90% of the proteins enriched at astrocyte synapse ensheathing processes did not differ significantly across genotypes in our NAc tissue dataset" to "suggest that astrocyte structure and capacity to regulate synapse function in the NAc are largely intact in the absence of microglia" is a bit of leap however (even more boldly stated in the rebuttal document: "support the idea that astrocyte ensheathment of synapses is largely preserved in KO mice"). Still 10% of proteins related to ensheathing procceses do change, which is then roughly the same percentage they see in their overall analysis.

We appreciate the reviewer's point and absolutely want to highlight the limitations of our data and point to additional studies that would be needed to further clarify findings.

To address this concern, we:

- Revised the text to highlight that our interpretation is based on convergent observations from histology, electrophysiology, and proteomics. We also add explicit suggestions about key experimental approaches that could further probe the status of astrocyte-synapse interaction in these mice. This portion of the results section now reads:

  "For a more unbiased evaluation of astrocyte-relevant proteins in our dataset, we also examined proteins identified by recent proteomic studies that profiled cytosolic proteins of striatal astrocytes, as well as proteins enriched in their synapse-ensheathing processes[44]. The majority (>90%) of these proteins did not differ in abundance between KO and WT mice in our NAc tissue proteomics dataset (Table S1, Figure S6B,C)[44]. Together with preserved astrocyte morphology (Figure S5) and mEPSC/mIPSC kinetics (Figures S2,S3), these data suggest that astrocyte structure and capacity to regulate basal synapse function in the NAc are largely intact in the absence of microglia. Nonetheless, it will be important for future studies to examine astrocyte synapse ensheathment at the ultrastructural level, to use cell-specific (rather than tissue level) proteomic approaches to examine astrocytes in KO mice, and to probe synaptic responses during high frequency stimulation and plasticity induction protocols."

- Revised text in the first paragraph of the discussion from "Proteomic and histological analyses also indicated that observed deficits in synaptogenesis are unlikely to be driven by perturbation of astrocyte-derived synaptogenic cues or adhesion proteins that promote synapse maturation, as these were unchanged in our dataset (Figure 3)."

  This now reads: "Proteomic and histological analyses also indicated that astrocyte morphology, astrocyte-derived synaptogenic cues, and adhesion proteins that promote synapse maturation, were unchanged in the absence of microglia (Figure 3)."

- Revised text in paragraph 5 of the discussion from "Our proteomics data also indicated that the reduced number of glutamatergic NAc synapses in P22 KO mice was unlikely to be due to perturbation of astrocyte derived synaptogenic cues or adhesive proteins that support synapse formation (Figure 3C and S5C). An alternative possibility is that microglia directly support NAc glutamatergic synaptogenesis and absence of this microglial support blunts synapse number."

  This now reads: "Our proteomics data also indicated that the reduced number of glutamatergic NAc synapses in P22 KO mice was not accompanied by reductions in

> detected astrocyte derived synaptogenic cues or adhesive proteins that support synapse formation (Figure 3C and S5C). This raises the possibility that microglia directly support NAc glutamatergic synaptogenesis and absence of this microglial support blunts synapse number."

We would also like to draw the reviewer's attention to paragraph 6 of the discussion where we added the following text as part of the initial manuscript revision: "Although we did not observe changes in detected astrocyte-derived synaptogenic cues (Gpc4, Gpc6, Sparcl1), some astrocyte-derived synaptogenic cues were not detected via proteomics (e.g. Tsp4, Thbs1, Thbs2, Sparc, Chrdl1) and we cannot exclude that these factors are present in the developing NAc at low levels and are perturbed by microglial absence. Moreover, S1pr1, which we found to be upregulated in KO mice, is expressed in perisynaptic astrocyte processes and regulates astrocyte morphology[75,76]. In neuron-astrocyte co-cultures, activation of S1pr1 increased astrocyte expression of synaptogenic cues such as Sparcl1 and Tsp4[75], an effect that required neuron-astrocyte contact. Hence, additional studies will be needed to fully define the status of astrocyte support for synaptogenesis in the absence of microglia."

A fundamental issue with the proteomic dataset is that this is tissue-level data: it is impossible to draw convincing conclusions from this data for specific cell types or cellular compartments, such as synapses or astrocyte-synapse contacts, as these are underrepresented in tissue extracts. Cross-referencing cannot solve this issue; cell type- or compartment-specific analysis or complementary approaches with higher resolution (such as immunohistochemistry for selected candidate proteins) can achieve this.

We completely agree that tissue-level proteomics has important limitations and we want to be transparent about highlighting those limitations.

As described above, we revised the text of the results to indicate that cell-specific proteomics and ultrastructural analysis in future studies are warranted for a more complete assessment of the status of astrocyte ensheathing processes.

We would also like to draw the reviewer's attention to paragraph 6 of the discussion (text from original submission) where we explicitly discuss key limitations of tissue-level proteomics and advocate for future cell- and compartment-specific approaches. "One limitation of whole-tissue proteomics is that these data cannot reveal whether some synapse-relevant proteins with normal abundance nonetheless have perturbed protein trafficking. Our proteomics pathway analysis indicated multiple hits related to intracellular protein trafficking (Figure 3), suggesting that approaches to investigate protein trafficking, particularly within neuronal dendrites and axons, should be a key future direction. Also, it is possible that decreased protein expression in one cell type could be masked by increased expression in another cell type. Future studies using cell-specific and/or cell compartment specific proteomics can be used to further explore the molecular mechanisms of how microglial absence impacts synapse development."

As the focus of this manuscript is identifying the specific synapse types, developmental periods, and behaviors that are impacted by microglial absence, we feel that deeper investigation of effects on astrocytes is beyond the scope of the current study.

Pt 6. The acute, NAc-specific depletion of microglia using CDS is an interesting experiment. The behavioral analysis shows partial overlap with the observation in FIRE mice, though differences are also present. However, the CDS experiment also raises new questions as to what extent this acute, more local manipulation affects morphological and functional synaptic parameters in the

NAc in early stages of development. To be able to compare the results of the CDS experiment with the previous dataset, a more systematic comparison of the different parameters analyzed is necessary.

We agree with the reviewer on this important point. To address this concern, we felt that the most valuable assessment of the impact of transient NAc microglial depletion on synapses would be direct functional assessment of synapses. To this end, we recorded miniature EPSCs from NAc medium spiny neurons in CDS-treated mice at P22. Consistent with our observations in KO mice, we observed a significant reduction in mEPSC frequency in CDS-treated mice. These findings suggest that local, transient depletion of NAc microglia is sufficient to recapitulate a key synaptic phenotype observed in mice that lacked microglia from birth. We also acknowledge that we did not systematically assess all morphological and functional features of synapses in CDS mice. Future studies will be necessary to determine the extent to which acute, local microglia depletion and constitutive, global microglia depletion produce overlapping effects on NAc synapse and circuit function.
